# Implementation and effectiveness of the web‑based skills training START NOW in adolescents in residential youth care: a cluster randomized controlled trial

**DOI:** 10.1186/s13034-026-01127-z

**Published:** 2026-06-29

**Authors:** Donja Brunner, Valentine Savary, David Bürgin, Linda Kersten, Janine Alfano, Nikki Rommers, Lelia Lanz, Chiara Chillà, Andreas Papageorgiou, Noortje Vriends, Christina Stadler, the START NOW Study Group, Madlaina Kapoor, Beryll von Planta, Nathalie Borel, Tabea Rocco, Arzie Bajrami, Jana Hurschler, Alex Traut, Catarina Fernandes De Brito, Mélanie Mayor, Stefan Weiss

**Affiliations:** 1 Research Department of Child and Adolescent Psychiatry, University Psychiatric Clinics Basel, University of Basel, Wilhelm Klein-Strasse 27, Basel, 4002 Switzerland; 2 Department of Clinical Research, University of Basel and University Hospital Basel, Basel, Switzerland

**Keywords:** Randomized controlled trial, Intervention, Adolescents, Residential youth care, Emotion regulation, Psychological flexibility, Resilience

## Abstract

**Background:**

Adolescents and young adults in residential youth care (RYC) face cumulated adversity and high emotional and behavioral problems yet face structural barriers that limit access to evidence-based mental health care. While digital tools promise scalability and flexibility, real-world implementation and effectiveness in vulnerable youth in institutional care remain understudied.

**Objective:**

This study aimed to evaluate the implementation and effectiveness of a newly developed web-based self-help or group-facilitated version of the START NOW intervention in a three-arm cluster-randomized control trial for youth in RYC in Switzerland.

**Methods:**

164 adolescents and young adults (M = 16.6 years, SD = 1.5; 43.8% female) from 27 RYC institutions were enrolled in a 9-week self-help or group-facilitated START NOW intervention tested against treatment-as-usual (TAU). Psychological inflexibility was the primary outcome; secondary outcomes included trait resilience, well-being, self-efficacy, functional impairment, and distress. Surveys were administered at baseline, post-intervention, 3-, and 6-month follow-up. Primary analyses employed linear mixed models in an intention-to-treat approach imputing missing data to estimate treatment effects at 3-month follow up. The trial was registered: ClinicalTrials.gov Identifier: NCT05313581; 6th April 2022.

**Results:**

The group-facilitated condition showed higher intervention compliance than the self-help help condition, over 50% of youth in the self-help group did not conduct a single session. Longitudinal follow‑up completion rates declined substantially, with only 37.8% providing data at T3 with the self-help group displaying the lowest levels (TAU 47.8%, 20.7% self-help, 48.3% facilitated group). No significant differences in psychological inflexibility or any secondary outcomes were found between study arms.

**Conclusions:**

In contrast to prior evidence from group-based START NOW interventions with more intensive facilitator support, the web-based START NOW adaptation evaluated in this found no evidence for additional benefits over TAU in RYC settings. Findings highlight critical implementation barriers to high attrition and missing data. Successful adoption of digital interventions in RYC require tailored, blended approaches with active facilitation and adaptive support structures.

**Supplementary Information:**

The online version contains supplementary material available at https://doi.org/10.1186/s13034-026-01127-z.

## Introduction

Adolescents and young adults in residential youth care (RYC) experience disproportionately high rates of mental health issues compared to peers in the general population, often linked to early-life adversity including trauma, abuse, and neglect [3, 8, 27, 46]. Risk factors such as limited social or family support, poor emotion regulation, and impulsivity further increase vulnerability and complicate the delivery of effective interventions in institutional settings [6, 12, 45]. Despite complex needs, access to evidence-based, trauma-informed interventions in these settings commonly remains limited [20], leaving staff to manage externalizing behaviors, such as aggression and opposition with often limited resources [36, 39].

START NOW is a structured, transdiagnostic group intervention originally developed by Trestman and colleagues for use in correctional environments [42]. START NOW trainers complete a training course that covers key elements of cognitive behavioral therapy, motivational interviewing, dialectical behavior therapy, acceptance and commitment therapy, and trauma-informed care [31]. The program targets emotion regulation, interpersonal skills, and psychological inflexibility (PIF), which are considered key components of mental health and resilience [19, 28]. Evidence supports its efficacy in reducing behavioral problems and improving psychological outcomes in particular in forensic populations [18, 30, 44].

START NOW was adapted for adolescents by Christina Stadler and her team at the University of Basel and subsequently evaluated in an international, multicenter randomized controlled trial (RCT) conducted in youth welfare institutions across Switzerland, Germany, and the Netherlands [38, 43]. Participants in the START NOW group demonstrated greater symptom reductions from baseline to follow-up, with a medium effect size indicative of a clinically meaningful delayed treatment response.

However, rigorously testing and sustainably implementing such interventions in RYC remains challenging due to high staff turnover, limited training capacity, and participant mobility [26, 48]. These structural constraints often undermine key prerequisites for successful implementation, such as continuity of facilitators and stable group composition. As a result, interventions with demonstrated efficacy under optimal conditions are rarely implemented at scale or evaluated in RYC. Thus, web‑based interventions have been proposed as one potential way to increase scalability and flexibility, enabling low‑threshold access, reducing stigma, and supporting continuity of care across placement changes [1, 15, 24]. Guided digital interventions have demonstrated comparable or superior outcomes to unguided approaches in other youth populations [4, 10, 16]. Such approaches have rarely been studied in vulnerable adolescents in institutional care [2, 34].

Thus, this study aimed to evaluate the implementation and effectiveness of a newly developed web-based version of the START NOW intervention, delivered both with and without guided facilitation, using a three-arm cluster RCT. A separate qualitative implementation study using a RE-AIM–based framework explores contextual and process factors of START NOW implementation in these institutions (Stadler et al., under review). In the RCT, primary and secondary outcomes were assessed at baseline (T1), post-intervention (T2), and at 3- and 6-month follow-ups (T3 and T4). We hypothesized that young people receiving the web-based group training guided by a facilitator would exhibit greater reductions in psychological inflexibility (PIF, primary outcome) at follow-up (T3) compared to those receiving treatment as usual (TAU). We further expected the web-based self-help training to outperform TAU, though to a lesser extent than the web-based facilitator guided group training. Secondary hypotheses for the RCT included improvements in resilience and reductions in symptoms of depression, anxiety, and irritability across both intervention arms. All hypothesizes were preregistered and the study protocol was published prior to data access (ClinicalTrials.gov NCT05313581, registered on 6 April 2022, study protocol: Kersten et al. [29, 37].

## Methods

### Study design and setting

This study evaluated the implementation and effectiveness of the START NOW WebApp in RYC across the German- and French-speaking regions of Switzerland. The chosen research design was a prospective, confirmatory RCT with two experimental and a control condition: (1) START NOW as web-based group training guided by a facilitator (trained START NOW facilitator, providing guidance through sessions and motivating change), (2) START NOW as web-based pure self-help training, and (3) TAU. The study was conducted as monocentric study by the Department of Child and Adolescent Psychiatry, University Psychiatric Clinics Basel and approved by the Ethics Committee of Northwestern and Central Switzerland (EKNZ; Protocol ID: 2022 − 00108). Funding was provided by the Swiss Federal Office of Justice. For full methodological details, including a list of major amendments following the initial trial registration, please refer to the published study protocol [29].

### Procedures

Participants were recruited from RYC institutions recognized by the Swiss Federal Office of Justice [7]. Institutions were informed about the study through mail, phone calls, and direct contact by the study team in Basel. Interested and eligible institutions received recruitment materials and provided institutional approval to participate. Out of 200 institutions contacted, 27 participated in the study (13.5%, 15 from the German-speaking and 12 from the French-speaking Switzerland). The most frequently cited reasons for non-participation were lack of staff or team resources (*n* = 32), not enough eligible adolescents (*n* = 21), and competing priorities or ongoing restructuring (*n* = 18). Additional barriers included low motivation among youth or staff (*n* = 14), structural or technical constraints (*n* = 11), and poor fit or timing of the program (*n* = 9). Potential participants were identified at these institutions and connected with the study team, which then provided full information, and written informed consent was obtained from all participants prior to inclusion. Once four eligible participants were recruited, the institution was cluster randomized at the institutional level into one of the three conditions using a pre-generated allocation list provided by the Department of Clinical Research, University of Basel and University Hospital Basel. Twenty‑seven residential youth care institutions (8 facilitator‑guided START NOW, 10 self‑help START NOW, 9 TAU) were cluster‑randomized at the institutional level. Cluster sizes varied between 4 and 12 included participants (mean cluster sizes per study arm were: 5.1 TAU, 5.8 self‑help, and 7.3 group‑facilitated, modus *n* = 4). Institutions typically contributed one group to the study, but larger sites could include consecutive or parallel groups in the same trial condition.

Data collection for the RCT was conducted digitally via secure, personalized REDCap [23] survey links at standardized study time points, with participant adherence supported by regular reminders and financial incentives for completed timepoints (CHF 20 per assessment, up to CHF 100 in total). Additional scaffolding included structured monitoring by the research team, active involvement of facilitators for group participants, technical support for survey access on mobile and desktop devices, and ongoing follow-up to ensure high data quality and participant comprehension. Following RCT completion, all participating institutions were granted unrestricted access to the WebApp for the remainder of the project phase.

### Intervention

The newly developed START NOW WebApp was based on the manualized START NOW skills training, aiming to enhance emotion and stress regulation, social competencies, coping strategies, and overall functioning. The development was closely supervised by the Department of Clinical Research at the University Hospital Basel and the University of Basel, ensuring compliance with data protection standards, user management protocols, and Good Clinical Practice guidelines. The WebApp consisted of 12 sessions (eight basic and four advanced) and conveyed the same core content as the original paper-based version for adolescents, with minor adjustments in the structure and combination of elements. Each session included mindfulness exercises, emotional and behavioural analysis, and thematic content on emotion acceptance, respectful relationships, and goal setting. Content was available in German and French. Participants could track their progress and earn trophies for completing exercises.

**Fig. 1:**
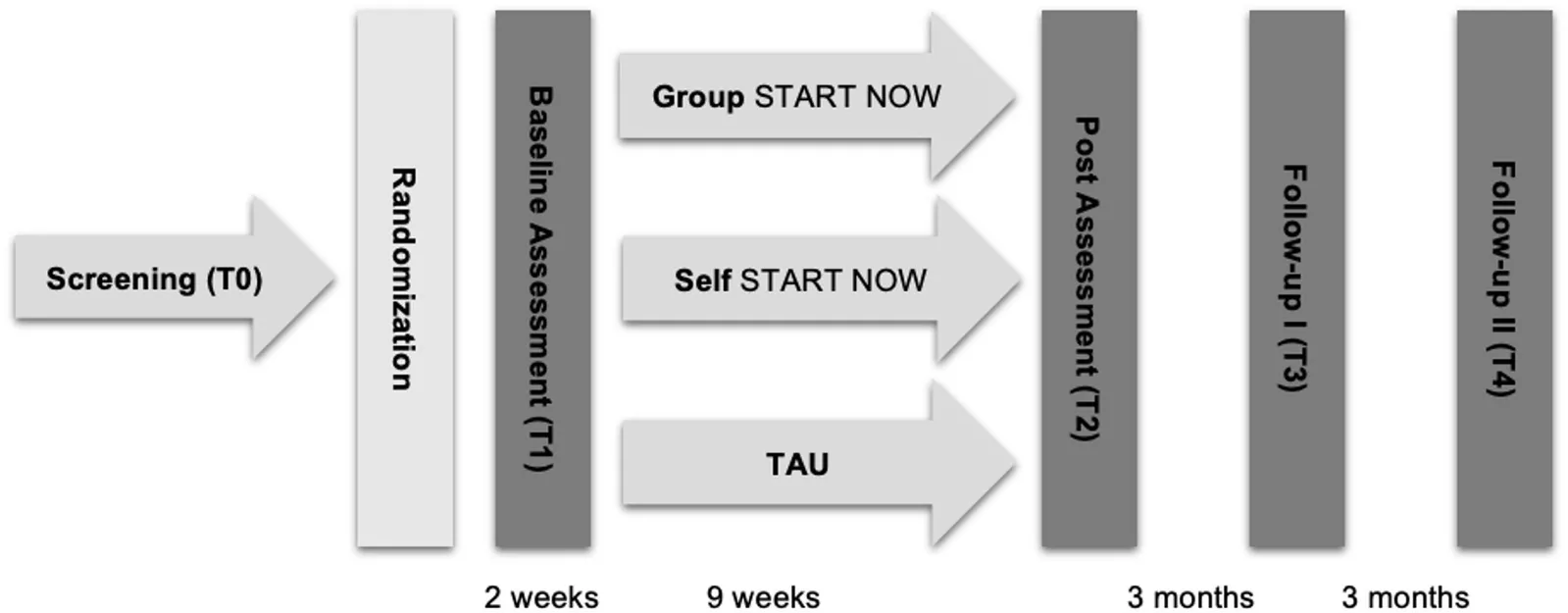
Overview of the START NOW WebApp trial design and procedures. The figure illustrates the screening and randomization process (T0), baseline assessment (T1), allocation to the three trial conditions (guided group START NOW WebApp, self‑help START NOW WebApp, and treatment as usual [TAU]), the 9‑week intervention period, post‑intervention assessment (T2), and the two follow‑up assessments at 3 months (T3) and 6 months (T4). Radomization was performed on the institutional level

Over a 9-week intervention phase, START NOW sessions unlocked weekly, with sessions 1–2, 9–10, and 11–12 delivered as double sessions (120 min). Participants completed the program either through 12 web-based group sessions (60–120 min) led by a facilitator or as self-help training (45–90 min). TAU participants received access to the self-help version after the study. An overview of the design and procedures are provided in Fig. 1.

### START NOW facilitator training and supervision

Interested staff members from participating institutions, as well as those appointed as facilitators, took part in a 12-hour pre-intervention training following a train-the-trainer model. The training covered theoretical foundations, practical use of the START NOW WebApp, techniques for promoting individual practice (in-vivo coaching), and motivational interviewing strategies to support behavior change. It was divided into three distinct blocks: Block 1: Theoretical background and introduction to START NOW (webinar format); Block 2: Orientation to the WebApp and associated training materials; Block 3: Planning and conducting group sessions using the WebApp, including supervision strategies. Institutions allocated to the group training guided by a facilitator received the full 12-hour training prior to the beginning of the intervention phase (Blocks 1, 2, & 3).

Each institution assigned to the group-facilitated arm was required to have at least two trained START NOW trainers. Supervision was provided to trained facilitators twice during the intervention phase: once following the second group session, and again during the first half of the intervention (~ 2 h). Additionally, facilitators and supervisors were encouraged to contact the designated START NOW study coordinator via email for ongoing support. The supervision aimed to guide implementation, address challenges, and enhance facilitators’ skills. In institutions randomized to the self‑help condition, START NOW was implemented as a purely web‑based, self‑guided program. Staff completed only the introductory blocks 1–2 to understand the WebApp and study procedures and were explicitly instructed not to facilitate or deliver therapeutic content. Their role was limited to informing adolescents about allocation at consent and reminding them to complete the online questionnaires, with only occasional ad‑hoc support for technical issues (for example, log‑in problems). Institutions in the TAU arm did not receive START NOW training until all intervention and survey phases had concluded, at which point interested staff from TAU and self‑help sites were offered the full training. Across all intervention institutions, 131 staff were trained in START NOW, and 26 of these acted as in‑session facilitators in the eight group‑facilitated institutions (on average about three facilitators per group‑facilitated institution, with a minimum requirement of two).

### Measures

*The primary outcome* was psychological inflexibility (PIF), assessed using the *Avoidance and Fusion Questionnaire for Youth *[21], a validated 17- item self-report measure. Items are rated on a 5-point Likert scale (0 = not at all true to 4 = very true), with higher total scores indicating greater PIF. The primary endpoint was the change in total AFQ-Y score from baseline (T_1_, within two weeks prior to intervention) to 12 weeks post-intervention (T_3_ +/- 2 weeks after end of intervention). Scores range from 0 to 68. The internal consistency of the AFQ-Y was high across all measurement points (Cronbach’s α = 0.88–0.89) and was within the range reported in previous validation studies.

*Secondary outcomes* included changes in the following self-reported psychological health indicators, assessed at T1 (baseline), T2 (post-intervention), T3 (12 weeks post), and T4 (24 weeks post): Resilience: *Connor-Davidson Resilience Scale* [9]; Psychological well-being: *World Health Organization - Five Well-Being Index* [41]; Self-efficacy: *General Self-Efficacy Scale* [35]; General impairment: *Columbia Impairment Scale* [5], both self- and caretaker-rated; Depression and anxiety: *Patient Health Questionnaire-4* [32, 33]; Anger-irritability: *Affective Reactivity Scale* [40], self- and caretaker-rated; Substance use: *Alcohol/Drug Use Subscale* of the *Massachusetts Youth Screening Instrument-2* [22].

*Additional variables* such as age, diagnosed mental disorder, previous institutionalization, and duration of institutionalization were tested as moderators. Youth and caregiver satisfaction were also assessed at T2 [29].

### Statistical analyses

A detailed description of all analyses conducted by the Clinical Trials Unit of the Department of Clinical Research at the University of Basel is available in the supplementary materials, including *Statistical aspects of the study protocol* (Supplementary Material 1), the *Report and analysis plan* (Supplementary Material 2), *Methods & Statistics* (Supplementary Material 3), and *Methods & Statistics II* (Supplementary Material 4). The following section provides an overview of the key procedures, along with the primary and secondary endpoints.

Sample size estimation was simulation-based, assuming normal distribution of AFQ-Y baseline scores (M = 21.04, SD = 13.01; [21]), equal variances across groups, and a within-subject correlation of *r* ≈ .80. Expected effect sizes were d = -0.50 (TAU vs. self-help) and d = -0.80 (TAU vs. facilitated group). Adjusting for clustering and an observed average group size of 4, along with an anticipated 20% dropout rate [38], a total of 150 participants (50 per group) were required (see Supplementary Material 1). The planned effect sizes (d = − 0.50 for TAU vs. self‑help, d = − 0.80 for TAU vs. group) were pre‑specified in the protocol to power the two TAU contrasts and were informed by CBT/ACT research [29], but in hindsight were optimistic relative to the previous START NOW youth study [38]. The self‑help versus group comparison was defined as exploratory and not used for sample size planning [29].

To analyze the outcomes, linear mixed-effects models (LMMs) were applied, accounting for the fact that data from adolescents within the same group or institution are not fully independent. The primary endpoint was the *AFQ-Y* score at follow-up. Group membership (e.g., TAU or treatment) was included as a fixed effect, while the institution was modeled as a random intercept to account for clustering and differences in baseline values. This approach allowed for precise estimation by accounting for both individual variability and group-level effects. The intervention (two conditions, with TAU as the reference group) was modeled as a fixed effect, and the baseline AFQ-Y score (T1) was included as a covariate. Model assumptions were tested by examining residuals. Secondary outcomes were tested using similar LMMs. None of the analyses were controlled for multiple testing. Primary and secondary outcomes were evaluated using an intention-to-treat (ITT) approach, which included all randomized participants. Per protocol sensitivity analyzes including only compliers (completed at least half of the sessions) were conducted for the primary and secondary outcomes (see Supplementary Material 2–3).

Multiple imputations were conducted at the item level using parcel‑summary methods, as outlined in detail in see Supplementary Material 2–3. For the primary outcome (AFQ‑Y), all missing items at T1 and T3 were imputed, while for secondary outcomes imputation was restricted to scales with limited item nonresponse (no imputation if < 4 items; if 4–9 items, ≤ 1 missing; 10–15 items, ≤ 2 missing; 16–25 items, ≤ 3 missing), with questionnaires exceeding these thresholds excluded from imputation. Overall, *n* = 50 imputed data sets were generated under a missing‑at‑random (MAR) assumption and analyzed using LMMs, with estimates combined according to Rubin’s rules (Rubin, 1987). As a sensitivity analyses complete case analyses were conducted for all models. All statistical analyses were conducted using R software, version 4.4.1 (R Core Team, 2023).

## Results

### Participants

**Fig. 2:**
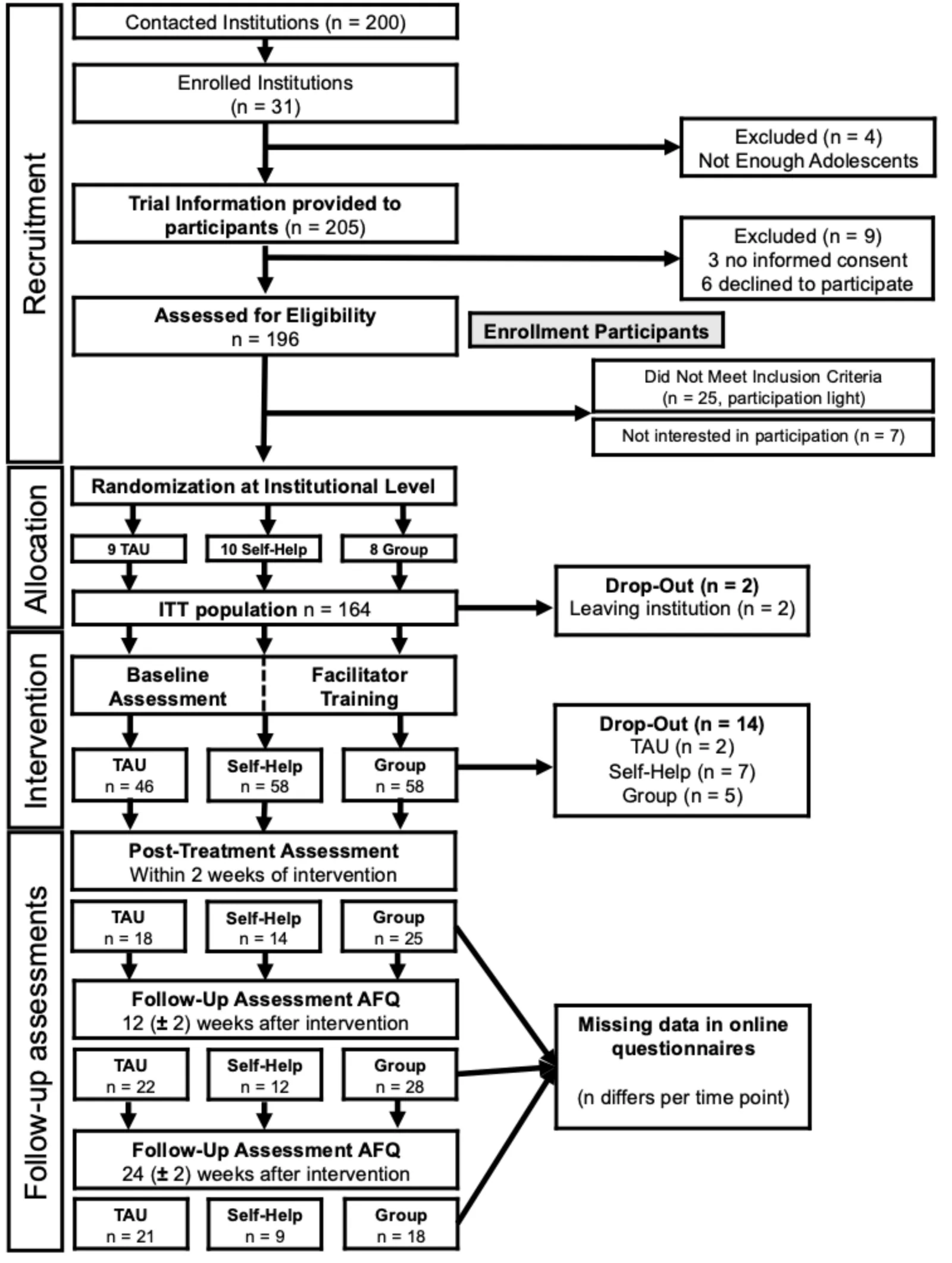
CONSORT flow chart of the START NOW WebApp trial. The figure shows the flow of institutions and adolescents through recruitment, randomization at the institutional level, intervention, and follow‑up assessments in the three trial arms: treatment as usual (TAU), START NOW WebApp self‑help, and START NOW WebApp guided group training. RCT = randomized controlled trial; ITT = intention‑to‑treat; AFQ‑Y = Avoidance and Fusion Questionnaire for Youth

Participants were 164 adolescents and young adults (M = 16.6 years, SD = 1.5; 43.8% female, 0.6% non-binary). After screening (T0), 164 adolescents were enrolled and completed the baseline assessment (T1). The sociodemographic characteristics are presented in Table 1. An overview of the recruitment and attrition processes provided in the CONSORT flowchart (Fig. 2). A significant group difference was observed with respect to living arrangements, largely driven by the TAU group, in which all participants resided within an institution more frequently. The TAU group also had a higher proportion of participants with criminal offenses and differed notably in school type compared to the other groups. These differences are likely due to the cluster randomization, which assigned specific centers to the TAU condition.

**Table 1:** Differences in sociodemographic characteristics between study arms

Age (years) M (SD)	Group (*n* = 58)	Self-help (*n* = 58)	TAU (*n* = 46)	*p*-value
Gender (%)	16.6 (1.4)	16.3 (1.2)	16.9 (1.6)	0.090
Male	32 (55.2)	34 (58.6)	24 (52.2)	0.584
Female	26 (44.8)	24 (41.4)	21 (45.7)	
Non-binary	0 (0.0)	0 (0.0)	1 (2.2)	
Country of birth (%)				0.651
Switzerland	36 (78.3)	35 (79.5)	30 (75.0)	
Other	10 (21.7)	8 (18.2)	10 (25.0)	
Unknown	0 (0.0)	1 (2.3)	0 (0.0)	
Mother tongue = other (%)	31 (67.4)	32 (72.7)	26 (65.0)	0.734
Lives in an institution (%)				**0.048**
Yes	37 (80.4)	37 (84.1)	40 (100)	
No	8 (17.4)	7 (15.9)	0 (0.0)	
Other	1 (2.2)	0 (0.0)	0 (0.0)	
Length of stay in this institution (%)				0.376
6 months or less	17 (37.0)	16 (36.4)	8 (20.0)	
6 months to 1 year	10 (21.7)	8 (18.2)	14 (35.0)	
1 to 2 years	11 (23.9)	8 (18.2)	9 (22.5)	
more than 2 years	8 (17.4)	12 (27.3)	9 (22.5)	
Previous placements in other institutions/foster families (%)				0.138
0	16 (34.8)	14 (31.8)	17 (42.5)	
1	12 (26.1)	16 (36.4)	5 (12.5)	
2	9 (19.6)	8 (18.2)	5 (12.5)	
3 or more	9 (19.6)	6 (13.6)	13 (32.5)	
Convicted of a criminal offence = No (%)	42 (91.3)	36 (81.8)	25 (62.5)	**0.004**
End date of placement within the next 6 months = No (%)”	36 (78.3)	33 (75.0)	29 (72.5)	0.824
School or vocational training (%)				**0.033**
Institution internal school	21 (45.7)	26 (59.1)	9 (22.5)	
Institution external school	6 (13.0)	8 (18.2)	13 (32.5)	
No school attendance	6 (13.0)	3 ( 6.8)	4 (10.0)	
Finished school, currently in vocational training	9 (19.6)	3 ( 6.8)	11 (27.5)	
Attending university	0 ( 0.0)	0 ( 0.0)	1 ( 2.5)	
unemployed	4 ( 8.7)	4 ( 9.1)	2 ( 5.0)	

### Treatment and protocol compliance and satisfaction with the intervention

Treatment compliance, operationalized dimensionally by the number of completed WebApp sessions, was significantly higher in the facilitated group condition compared to the self-help condition (group condition: median 10 sessions, IQR 8–12 vs. self-help median 0 sessions, IQR 0–6; Mann–Whitney U test (two‑sided), *p*<.001). While 37.0% of participants in the group condition completed all 12 sessions, only 15.1% did so in the self-help condition. Notably, 50.9% of adolescents in the self-help condition did not complete a single session. In contrast, 87.0% of those in the group condition completed at least half of the sessions. ‘Compliers’ (participants adhering to their allocated START NOW treatment protocol, that is, completing at least 50% of sessions in the intervention arms or not receiving the START NOW in the TAU condition; *n* = 109) did not differ from ‘non-compliers’ (*n* = 55) considering any of the demographic or baseline screening variables (Supplementary Material 3, Tables 23–25).

Satisfaction data was available from 23 adolescents from the group-facilitated (39.7%) and 15 adolescents from the self-help condition (24.1%). Among adolescents in the facilitated group condition, many valued the illustrations, mindfulness practices, and relationship support, while some suggested shorter videos and greater variety of exercises. In the self‑help condition, adolescents who engaged with the program mostly reported positive experiences with stress‑ management and the exercises, though a few expressed dissatisfactions without specific reasons. Trainers generally appreciated the clear language, integration of mindfulness exercises, and use of visual elements, and emphasized the training’s practical relevance and perceived positive effects on participating adolescents. Several trainers also reported challenges related to adolescents’ limited engagement and institutional barriers to scheduling and maintaining group formats.

### Survey retention across study conditions

**Fig. 3:**
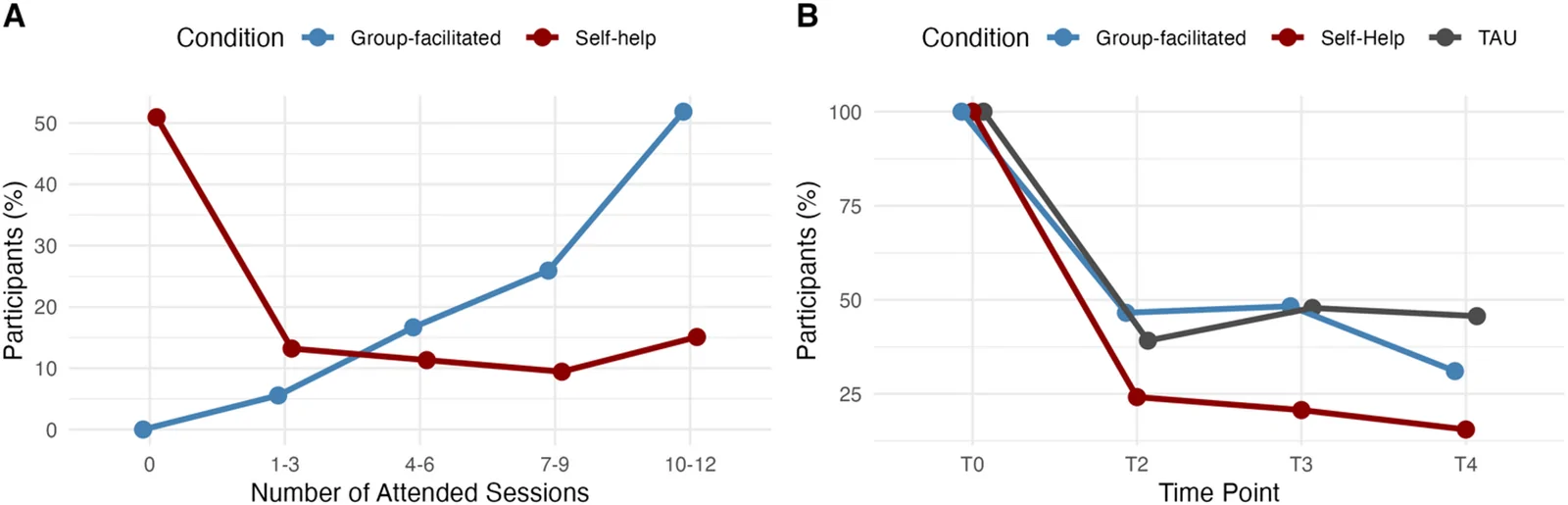
Treatment compliance and survey retention across study conditions. **A** Distribution of participants (%) by number of attended sessions for the group-facilitated and self-help intervention conditions. Percentages are calculated within each intervention arm using the total number of randomized participants in that arm as the denominator (group‑facilitated *n* = 58; self‑help *n* = 58). **B** Retention rates (%) over time at baseline (T0), post-intervention (T2), and follow-ups (T3, T4) for the group-facilitated, self-help, and treatment-as-usual (TAU) arms. For each time point, the percentage represents the proportion of participants with available AFQ‑Y data out of all randomized participants in the corresponding arm (TAU *n* = 46; self‑help *n* = 58; group‑facilitated *n* = 58)

Follow‑up completion rates declined substantially, with only 37.8% providing data at T3 (TAU 47.8%, 20.7% self-help, 48.3% facilitated group) and 29.3% at T4 (TAU 45.7%, 15.5% self-help, 31.0% facilitated group). Retention rates differed significantly between trial arms, with the self-help group displaying the lowest levels (T3: $\:{\chi\:}^{2}$(2) = 11.82, *p*=.003, and T4 ($\:{\chi\:}^{2}$(2) = 11.26, *p*=.004; see Fig. 3B).

### Primary outcome based on intention-to-treat (ITT), per protocol, and complete-case analyses

**Fig. 4:**
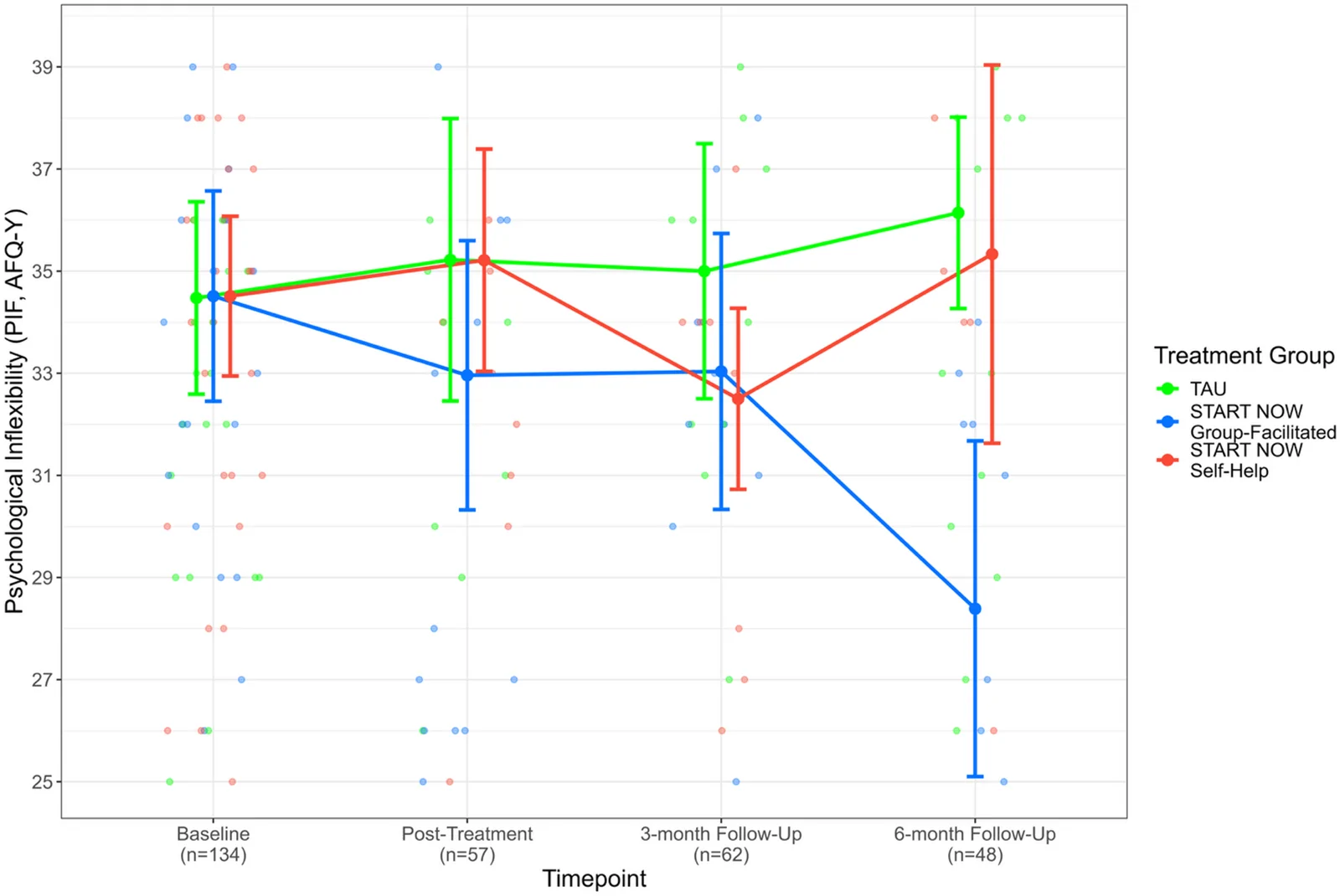
Main outcome of the RCT across all study arms and timepoints. Jittered points include individual datapoints. x-axis displays each timepoints including number of available data for main endpoint. y-axis shows per group mean values and standard errors of Psychological Inflexibility (PIF, AFQ-Y Total). Jittered points display available raw data across all timepoints

The statistical analysis of the primary outcome examined the effect of the intervention conditions (web-based group training guided by a facilitator, web-based self-help training, TAU) on the total score of the AFQ-Y at follow-up (T3). The results are summarized in Table 2, including estimated coefficients, 95% confidence intervals (CIs), and *p*-values for the intervention conditions as well as all covariates. Figure 4 displays mean AFQ‑Y scores across all four time points by study arm. Descriptively, AFQ‑Y trajectories appear relatively flat across all arms, with no clear indication of systematic improvement in psychological inflexibility in TAU or the self‑help condition, and only a modest downward trend at T4 in the facilitator‑guided group (see Fig. 4). Primary analyses from ITT-analyses indicate no significant differences in AFQ-Y total scores at T3 between the TAU group and either of the two intervention conditions. Thus, results indicate that the web-based skills training with group-based guidance did not lead to a greater reduction in PIF compared to TAU (Hypothesis 1). Similarly, the web-based self-help intervention showed no greater reduction in PIF compared to TAU (Hypothesis 2). Further, there was no significant difference in PIF reduction between the group-based and self-help START NOW conditions (Hypothesis 3).

**Table 2:** Results of the primary analysis (hypothesis 1 & 2) in an intention-to-treat (ITT) analyses 12-weeks post-intervention (T3)

Predictor	Estimate	CI lower	CI upper	*p*-value
Group over TAU (Hyp. 1)	-1.222	-7.057	4.613	0.678
Self-help over TAU (Hyp. 2)	-1.124	-7.083	4.834	0.708
Group over self-help (Hyp. 3)*	-0.097	-5.558	5.363	0.972
AFQ total at T1	0.296	0.090	0.503	**0.005**
Age (years)	0.359	-1.281	2.000	0.664
Gender (female over male)	1.528	-3.480	6.536	0.545
Gender (diverse over male)	1.243	-32.176	34.662	0.941
MAYSI II AI	-1.073	-2.293	0.147	0.084
MAYSI II DA	-0.025	-1.246	1.196	0.968

*Effect from a second model in which the reference category was from TAU to self-help. All other estimates in the model are exactly the same. TAU=treatment-as-usual. AFQ-Y= Avoidance and Fusion Questionnaire for Youth; MAYSI-II= Massachusetts Youth Screening Instrument-2; AI=anger/irritability; DA=drug/alcohol use

Per protocol analyses including compliers only (*n* = 109) also showed no significant effects (Supplementary Material 3, Table 16). Complete case sensitivity analyses (*n* = 59) similarly showed no significant difference between study arms (see Supplementary Material 3, Table 19). At the second follow-up (T4), the results also showed no significant differences in the change in PIF between the intervention conditions (see Supplementary Material 3, Table 15). Additional moderation analyses showed no significant interaction between participant age, diagnosed mental disorder, previous institutionalization, and duration of institutionalization with any treatments effect (see Supplementary Material 3, Tables 84, 85, 86 and 87).

### Secondary outcome analyses

Changes in general resilience, self-efficacy, depressive and anxiety symptoms, irritability, general functional impairment, and the social climate within the setting were analyzed in secondary analyses. Secondary outcome imputation differed (did not impute entirely missing scales), thus, many participants with entirely missing scales were dropped from secondary analyses. As a result, the number of participants included in secondary outcome variables varied by questionnaire (*n* = 40–164). No statistically significant differences were found between the treatment conditions for any secondary outcome variables (see Supplementary Material 3, Table 14). Furthermore, per protocol analyses including compliers only also showed no significant results regarding any of the secondary endpoints (Supplementary Material 3, Table 16).

### Further supplementary analyses

In further supplementary analyses, group differences between treatment group compliers and all other participants were tested across all outcome measures and post-intervention timepoints on complete data (*n* = 29–88). In these analyses, no consistent patterns of treatment effects were found (Supplementary Material 4, Tables 1, 2 and 3). Dose-response analyses testing linear interactions of number of sessions with all outcomes indicated that a higher number of attended sessions might be associated with improved scores in some social environment subdomains and lower caretaker-rated problem behaviors, although these effects were inconsistent, based on small samples and not controlled for multiple testing (Supplementary Material 4, Tables 4, 5 and 6).

## Discussion

This cluster RCT found no significant benefit of the e-version of START NOW, either as facilitator-guided group training or as a self-help web-based intervention, against TAU for reducing psychological inflexibility (PIF) or secondary mental health outcomes among youth in Swiss RYC. Treatment compliance was substantially higher among participants in the group-facilitated condition, yet even with facilitation, overall treatment compliance and study retention was challenging and no consistent positive effects of the intervention was detected. Attrition rates were very high across all study arms and were especially pronounced in the self-help intervention. These findings highlight major implementation barriers to digital mental health programs in real-world youth care environments. An initial intention to treat is not equivalent to a genuine motivation and capacity to comply with an intervention or adhere to a research protocol. Displaying no real-world effectiveness under implementation constraints, reflects the limits of digital programs when professional support or participant engagement are insufficient, and does not contradict the general efficacy of an intervention. Overall, the results temper optimism for broad, unguided digital rollout and underscore the necessity of active institutional and facilitator support for meaningful digital intervention reach and impact in high-need populations.

### Real-world effectiveness of the e-version of START-NOW in youth residential care

Contrary to our hypotheses, neither the web-based group-based nor self-help versions of the START NOW intervention resulted in greater reductions in PIF compared to TAU at 3- and 6-month follow-up, and secondary outcomes likewise showed no significant intervention effect. In contrast to earlier START NOW trials that reported improvements relative to usual care [30, 38, 44], and other digital youth interventions [18, 34], the present study found no evidence for additional benefits of the WebApp over TAU, and the descriptive trends in AFQ‑Y scores do not suggest meaningful improvement in TAU alone. A range of factors may explain these divergent results, including differences in study design (ITT vs. per-protocol), diverse outcome measures (narrower focus in delinquent behaviors vs. broader functioning and resilience measures), different observation windows and inclusion criteria, different levels of facilitator support, insufficient power, too optimistic effect size estimates, and substantial attrition and missing data; many of which are common real-world implementation barriers in RYC [13, 26, 47]. Compared with prior START NOW implementations that relied on more intensive, in‑person facilitator training, in-person supervision and close study‑team contact with youth [38], the present WebApp trial deliberately reduced facilitation intensity and personal scaffolding, which may have limited the development and use of therapeutic coaching skills in everyday interactions. The lack of statistically significant between‑group differences, together with the reduced effective sample size and extensive missing data, indicates that the trial was likely underpowered to detect modest effects under real‑world conditions. Recruiting substantially more participants in closed residential institutions was not feasible within the funding and time frame of the project. Accordingly, the null findings should be interpreted cautiously, as they may reflect limited power and substantial implementation barriers rather than clear ineffectiveness of the START NOW WebApp. These results challenge assumptions about broad digital intervention rollout in RYC settings, highlighting the critical need for active facilitator engagement and organizational support to realize intended benefits of digital interventions, and urge for more direct youth involvement in co-designing and co-implementing interventions for underserved youth [2].

### Implementation challenges and retention in longitudinal research in vulnerable populations

This study highlights multifaceted implementation challenges encountered when delivering and studying digital mental health interventions in RYC, a population already marked by high rates of trauma exposure, family disruption, and complex mental health problems [3, 8, 27, 46]. Recruitment and implementation were hampered by institutional reforms, understaffing, and high turnover among staff and residents, necessitating multiple protocol adjustments [29, 37]. Moreover, the trial suffered from lower-than-anticipated compliance and higher longitudinal attrition, particularly due to high youth mobility and competing institutional demands, being most pronounced in the digital self-help arm. These patterns are consistent with the study design of the self‑help arm, in which START NOW was offered as an unguided WebApp and institutional staff were instructed to provide only minimal, non‑therapeutic support, rather than active facilitation. These findings reinforce that facilitative scaffolding remains critical for both treatment and research engagement in this setting. Importantly, such real-world barriers are better conceived as key determinants of intervention access and effectiveness in high-need populations than as study limitations. They align with widespread concerns about accessibility, implementation, and sustained engagement in evidence-based interventions in youth residential care [13, 14, 26] and about scalability, low engagement, and high attrition in digital interventions in real-world settings [17, 25]. To increase the real-world impact of digital and group-based interventions in RYC, future work should emphasize flexible, individualized engagement and hybrid delivery models that combine group and one-on-one support [11]. Training multiple facilitators per institution and supporting ongoing skill development can embed interventions organization-wide, not just in single groups [26]. Addressing institutional priorities, piloting program phases, and providing targeted incentives or supportive policy will help bridge the persistent gap between research and routine practice [13]. More mixed-methods implementation research is needed in institutional settings to assess not only the reach and effectiveness, but also the adoption, compliance of implementation, and sustainability of interventions under real-world conditions [2, 25].

### Limitations and strengths

This study has several important limitations. First, substantial barriers to implementation resulted in both lower compliance to treatment and longitudinal attrition, leading to high amounts of missing data and requiring substantial statistical imputation. Because imputations were needed for 50–70% of outcome data at some time points, this introduces considerable uncertainty about the robustness of findings; however, sensitivity analyses based on the smaller sample of completed cases, while underpowered, produced the same overall conclusions. Second, the study was powered based on relatively optimistic effect size assumptions (medium to large effects for the contrasts with TAU). In practice, high attrition and substantial missing data substantially reduced the effective sample size, so that the trial was likely underpowered to detect small to moderate effects, particularly for comparisons between the two active interventions. Consequently, the null findings should not be interpreted as strong evidence for the absence of effects. Third, cluster randomization and institutional recruitment led to unbalanced group allocation regarding baseline characteristics (e.g., more high-risk youths or certain institutions in TAU), which may confound group comparisons. Moreover, findings are specific to the unique institutional landscape of Swiss RYC and may not generalize to other national contexts or care models. Fourth, primary and secondary outcomes focused on short-term (3–6 month) trajectories, thus, the study may have missed delayed or longer-term intervention effects observable only after extended follow-up. Fifth, we did not conduct formal fidelity monitoring of session facilitation in the guided group arm (e.g., independent rating of sessions). Consequently, conclusions about the effectiveness of the intervention cannot be clearly separated from potential variability in how closely facilitators followed the START NOW protocol. Last, important contextual or individual moderators (e.g., history of trauma, pedagogical attitudes, etc.) were not systematically measured, limiting the ability to identify for whom and under what conditions the interventions might be effective. Last, a key limitation of this study is the high proportion of missing questionnaire data at T3 (and other visits), resulting in a large fraction of imputed values for the primary endpoint. Multiple imputation can reduce bias under the missing at random (MAR) assumption, but with extensive missingness and likely links to unobserved factors (e.g. motivation, symptom burden, adherence), estimates become heavily model-dependent and potentially biased if data are missing not at random. Therefore, the results should be viewed as tentative, and any apparent treatment effects or null findings interpreted with particular caution.

Several important strengths are noteworthy. First, this study is the first cluster randomized controlled trials assessing a digital mental health intervention in Swiss residential youth care, providing direct insight into both implementation and effectiveness in a high-need context. Second, the inclusion of a diverse range of institutions from both French- and German-speaking regions of Switzerland enhances generalizability and reflects real-world heterogeneity in organizational culture and resources. Third, the comprehensive collection of process and implementation data, alongside outcome measures, enabled a nuanced understanding of institutional, staff, and participant factors affecting engagement, feasibility, and sustainability. As such, the study integrated youth and caregiver perspectives, capturing satisfaction and perceived usefulness. Finally, transparent documentation of adaptations, deviations, and missing data, and the use of sensitivity analyses, increase the credibility and practical value of findings for both researchers and practitioners seeking scalable solutions in complex youth care settings.

## Conclusion

This study highlights both the potential and the limitations of implementing web‑based interventions such as the START NOW WebApp in RYC settings. Although the RCT did not demonstrate significant effects on resilience or other targeted outcomes, this may be partly explained by implementation challenges, low adherence, and substantial missing outcome data. The findings suggest that purely self‑guided digital interventions may not be suitable for this population. Instead, blended approaches that combine digital tools with personalized guidance from well‑trained staff, ideally in individual or small‑group formats, appear more promising. Meaningful implementation of such approaches within stepped‑care models is needed, combining blended digital formats with more specific, evidence‑based therapeutic interventions. Future efforts should focus on tailoring digital interventions to the specific needs of both youth and institutions, promoting flexibility, personal relevance, and motivation. With appropriate support structures, digital tools such as the START NOW WebApp can still contribute meaningfully to the promotion of self‑regulation and resilience.

## Supplementary Information

Below is the link to the electronic supplementary material.

Supplementary Material 1.

Supplementary Material 2.

Supplementary Material 3.

Supplementary Material 4.

## Data Availability

Data is available upon reasonable written request to the Prof. Christina Stadler. Any sharing of data will be based on a data sharing agreement under Swiss law and local jurisdiction.
