# Supplementary material for: Implementation and effectiveness of the web‑based skills training START NOW in adolescents in residential youth care: a cluster randomized controlled trial
Source: Child Adolesc Psychiatry Ment Health. 2026 Jun 29;20:131. doi: 10.1186/s13034-026-01127-z (PMC13591857; doi:10.1186/s13034-026-01127-z)
Supplement: Supplementary file 1 [file 13034_2026_1127_MOESM1_ESM.pdf]

Universität Basel
Departement
Klinische Forschung

Universitätsspital Basel

Universität Basel, Departement Klinische Forschung, c/o Universitätsspital Basel, Spitalstrasse 12, 4031 Basel

Prof. Dr. Dr. Christina Stadler
Klinik für Kinder und Jugendliche der
Universitären Psychiatrischen Kliniken
Schanzenstrasse 13
4056 Basel

Basel, March 30, 2023

Statistical aspects of the study protocol

Study Title	Implementierung einer e-Version des Skillstrainings START NOW zur Förderung der Emotionsregulation und Resilienz in Institutionen des Massnahmenvollzugs
Internal DKF ID	pk19Stadler
Document Status	Final Report
Note	*The following sections are building blocks for the corresponding sections of the study protocol and need to be adapted to the context. Please send a version of the study protocol to the author for review before you submit or publish the protocol or refer to the Department of Clinical Research or the author.* *The numbering of the sections is based on the template for a study protocol at* *swissethics.ch*

Final Report

Background

Resilience is the ability to positively adapt to different life-situations. Resilient people are solution-oriented and show a high level of adaptability when faced with stress. They remain more confident about their future and find it easier to accept crises. Such competencies are valuable resources for psychological well-being, that come with lower levels of anxiety and depression and are linked to successful life outcomes. Resilience can also mitigate the effects of early trauma on depressive symptoms.

A variety of resilience-oriented training programs have been developed to enhance resilience. One such approach is START NOW. START NOW is an evidence-based, integrative skills training program, which includes a manual with detailed instructions for group facilitation. The

Universität Basel
Departement Klinische Forschung
c/o Universitätsspital Basel
Spitalstrasse 12
4031 Basel, Switzerland

Nikki Rommers, PhD
Statistician, Department of Clinical Research
Telefon +41 328 77 14
nikki.rommers@usb.ch
dkf.unibas.ch

program combines aspects of cognitive behavioral therapy (CBT), motivational interviewing (MI), dialectical behavior therapy (DBT), acceptance and commitment Therapy (ACT) and trauma-sensitive care to improve general resilience by enhancing emotion and stress regulation skills.

Although START NOW is highly suited for youth displaying low levels of resilience placed in restrictive settings, some obstacles arise when implementing the program in residential youth care. Such obstacles are hard to overcome and limit a program's effectiveness.

A promising solution to the aforementioned implementation difficulties are web-based e-mental health approaches. E-mental health approaches are defined as "a primarily self-guided intervention programme that is executed by means of a prescriptive online programme operated through a website and used by consumers seeking health- and mental-health related assistance".

Pure web-based self-help training has been shown to be moderately effective. Web-based trainings including social support from an e-coach (guided e-learning) show greater effects and better adherence. With regard to dropout, dropout rates for e-mental health programs are lower when the intervention is provided with support as compared to when it is provided without support, suggesting that e-mental health programs with support are superior to those without.

This project aims to develop and evaluate a guided e-mental health intervention that could bridge the gap between the high prevalence of mental health problems in adolescents at risk and limited access to mental health programs and/or limited personal resources in youth welfare institutions. The study will investigate the efficacy of an e-START NOW approach combining a web-based application of START NOW and support by a trainer (online or face-to-face) on psychological flexibility.

The study is a prospective multi-center 3-arm randomized controlled trial, where START NOW in the format of (1) guided by a facilitator (either face-to-face or through videoconferencing) web-based group training with (e-)coach will be compared to (2) web-based pure self-help training and (3) a waitlist treatment as usual (TAU) control group.

The treatment (guided web-based vs. self-help training vs. TAU) will be applied at the level of the center, the reason being that staff members (caretakers) of the institutions/centers subjected to condition (1) will receive a 12-hour training in START NOW, with potential "spillover" effects since such training may modify their every-day practice. Additionally, participants in condition (1) will be required to attend weekly sessions in groups including 4 to 12 participants.

After **baseline assessment (T1)** done 2 weeks before the start of the intervention, as soon as 4 eligible participants are found within one institution, the respective institution will be randomly assigned (n = 4-10 per group/institution) to one of the three conditions. The **intervention phase** will last for 9 weeks. A **post-treatment assessment (T2)** will be done within 2 weeks after the end of the intervention. A **first follow-up assessment (T3)** will be done 12 weeks ($\pm$ 2 weeks) after the end of the intervention. A **second follow-up assessment (T4)** will be done 24 weeks after the end of the intervention.

11 STATISTICAL METHODS

Detailed methodology for summaries and statistical analyses of the data collected in this study will be documented in a report and statistical analysis plan (RAP). The RAP will be finalized

Final Report

before database closure and will be under version control at the Clinical Trial Unit, University Hospital Basel.

11.1 Hypothesis

11.1.1 Primary hypothesis

The primary objective of this study is to investigate, using a randomized controlled trial, whether in a population of youth and young adults (aged 14-24) placed in residential youth institutions, web-based START NOW guided group training and web-based START NOW self-help training are more efficient than treatment as usual (TAU) in decreasing psychological *in*flexibility (primary outcome) as measured by scores on the AFQ-Y questionnaire (primary endpoint) 12 weeks $\pm$ 2 weeks (T3) after the end of the intervention.

This leads to formulating the following null and alternative hypotheses.

TAU vs. web-based self-helped training

H_0: Mean inflexibility, as assessed by the Avoidance and Fusion Questionnaire for Youth (AFQ-Y), is the **same** in participants receiving treatment as usual (TAU) and in participants receiving a web-based START NOW self-helped training.

H_1: Mean inflexibility, as assessed by the AFQ-Y, is **not the same** in participants receiving treatment as usual (TAU) and in in participants receiving a web-based START NOW self-helped training.

TAU vs. web-based group training

H_0: Mean inflexibility, as assessed by the AFQ-Y, is the **same** in participants receiving treatment as usual (TAU) and in participants receiving a web-based START NOW group training .

H_1: Mean inflexibility, as assessed by the AFQ-Y, is **not the same** in participants receiving treatment as usual (TAU) and in participants receiving a web-based START NOW group training.

11.1.2 Secondary hypotheses

The secondary hypotheses are the following:

1. We hypothesize that the web-based START NOW guided group training performs better than the web-based START NOW self-helped training in reducing psychological inflexibility as assessed by the AFQ-Y score.

2. We hypothesize that web-based START NOW guided group training and web-based START NOW self-helped training will result in enhanced general resilience, self-efficacy and well-being and in reduced general impairment, perceived stress, irritability, psychopathology, conduct and emotional problems compared to TAU.

Final Report

3. We hypothesize that web-based START NOW guided group training will result in a greater enhancement of general resilience, self-efficacy and well-being, and in a greater reduction of general impairment, perceived stress, anger and irritability, psychopathology, conduct and emotional problems compared to web-based START NOW self-helped training.

4. We hypothesize that START NOW training courses for caretakers will lead to enhanced resilience in caretakers compared to the TAU.

5. We hypothesize that the effects of web-based START NOW group training and web-based START NOW self-helped training will be moderated by the number of sessions that have been conducted and by the work strain of the caretakers. We expect greater work strain to weaken the training courses' effect on caretakers' resilience.

6. We expect that a negative atmosphere in residential youth care (moderator) will weaken the intervention's positive effect on resilience.

7. We expect that greater severity/comorbidity in terms of alcohol/drug use, anger-irritability and depressive-anxious symptoms (moderator) will buffer the intervention's positive effect on resilience.

Final Report

11.1.3 Primary endpoint

The primary endpoint is psychological *in*flexibility as assessed by the score on the AFQ-Y questionnaire at follow-up T3, i.e. 12 weeks $\pm$ 2 weeks after the end of the intervention. The AFQ-Y is a validated self-rating questionnaire for assessing psychological flexibility in youth and young adults. Participants answer 17 items on a 5-point Likert scale. Scores range from 0 to 68. *Higher* total scores indicate *lower* psychological flexibility.

11.1.4 Secondary endpoints

All secondary endpoints will be assessed at baseline (T1) 2 weeks before the start of the intervention, 2 weeks after the end of the intervention (T2), 12 weeks after the end of intervention (T3) and 24 weeks after the end of intervention (T4).

1. General resilience: Total score on the German version of the Connor-Davidson Resilience Scale for both participant and caretaker (CD-RISC). 10-item version with 5-point Likert-scales (0-4); total score ranges from 0 to 40. *Higher* scores indicate *greater* resilience.

2. Psychological well-being: Total score on the World Health Organization Well-Being Index (WHO-5). 5 items on 6-point Likert-scales (0-5); total score ranges from 0 to 25. *Higher* scores indicate *higher* well-being.

3. Self-efficacy: Total score on the German "General Self-Efficacy Scale" (Skala zur Allgemeinen Selbstwirksamkeitserwartung, SWE). 10 items on 4-point Likert-scales (1-4). Total score ranges from 10 to 40. *Higher* scores indicate *higher* convictions on subjective controllability or competence expectations.

4. General impairment: All sub scores and total score on Columbia Impairment Scale (CIS), both in self-rating and external rating. 13 items on 5-point Likert-scales (0-4). Score ranges from 0 to 52. *Higher* scores indicate *greater* impairment.

5. Perceived stress: Total score on Perceived Stress Scale (PSS-4). 4 items on 5-point Likert-scales. Score ranges from 0 to 16. *Higher* scores indicate *higher* stress.

6. Anger-irritability: Total score on Affective Reactivity Scale. 7 items on 3-point Likert scales (0-2). Only the 6 first items are summed up into a total score that ranges from 0 to 12. The 7th item is analyzed separately. *Higher* scores indicate *higher* irritability.

7. Emotional and behavioral symptoms: All sub scores and total score on the Strength and Difficulties Questionnaire (SDQ). 25 items on 3-point Likert-scales (0-2). Total score ranges from 0 to 50. *Higher* scores indicate *greater* emotional and behavioural difficulties.

8. Participants within the two intervention conditions will rate their satisfaction with the training using the Client Satisfaction Questionnaire (CSQ), specifically developed to assess a participant's experience and satisfaction with START NOW. 8 items on 4-point Likert-Scales (0-3); response options are provided with smileys; two open questions about what participants liked or would want to be changed within the training.

Final Report

11.1.5 Covariables/Moderators

The following covariates will be used as "moderators" in secondary analyses of the effect of the treatment, i.e. they will be included in the model together with their interaction term with the treatment.

1. Psychopathology: Participants psychopathological state will be assessed by the Massachusetts Youth Screening Instrument-II (MAYSI-II). 42 items in six subscales: Alcohol/Drug Use, Anger-Irritability, Depression-Anxiety, Somatic Complaints, Suicide Ideation and Thought Disturbance. The MAYSI will be used as a screening instrument regarding the exclusion criteria. A reduced version based on the subscales Alcohol/Drug Use, Anger-Irritability and Depression-Anxiety will be used as a moderator.

2. Social Atmosphere: It will be assessed in youth by the German version Essen Climate Evaluation Schema (Essen CES-D). 17 items on 5-point Likert Scales. Total score ranges from 0 to 68 with higher scores indicating a better social climate.

3. control-Q: access to web application, unauthorized leaves of participants, caretakers as external rates.

4. BSQ: burden in staff.

5. Caretaker-Q: prior experience with START NOW, work experience, etc.

11.2 Determination of sample size

11.2.1 Choice of a model

Psychological *in*flexibility, as assessed by the AFQ-Y score, will be measured at baseline (T1) and 12 (+/- 2) weeks after the end of the intervention (T3). **The primary endpoint is Psychological inflexibility at T3**, as measured AFQ-Y scores. We are interested in comparing scores obtained by participants in the three conditions (TAU vs. web-based self-help training START NOW vs. web-based group training START NOW) 12 weeks after the end of the intervention (T3), accounting for their baseline score.

As a general rule, the intervention condition will be applied at the institution level, i.e. a given institution will be assigned only one condition. In large institutions with several independent living groups and staff per units, more than one experimental conditions will be applied. Knowledge about social dynamics and professional practice in institutions indicates that groups can be considered independent within institutions. We will thus and hereafter assume that the institution level does not need to be accounted for in the analysis. In contrast, in the group-training condition participants are not fully independent, thus generating pseudo-replication and intraclass correlation, i.e. a resemblance in the scores of participants within the same group. Such intraclass correlation could be accounted for using a mixed linear model including the identity of the group as a random factor. However, this "group" effect concerns only the intervention arm, because the web-based group training is always provided in a group, while the other conditions (TAU and self-helped) are not. Notably, no information is available about the possible extent of this intraclass correlation.

Consequently, it was decided to estimate sample size using a linear model (ANCOVA) including the score at T3, i.e. 12 weeks after end of intervention, as the dependent variable and the intervention (TAU, self-helped training, group training) and the baseline score (T1) as explanatory factor and covariate respectively.

The estimated sample size is then adjusted for the "group" effect by correcting for the "design effect" using the formula provided by Kish (1965) and for a range of credible intraclass correlation coefficients.

Final Report

11.2.2 Assumptions

The sample size estimation is based on the following assumptions:

- AFQ-Y scores are drawn from a rectified normal distribution, so that scores range from 0 to 68.
- Information for the sample size estimation is derived from Table 3 in Greco et al. (2008). It is assumed that numbers provided for 9th & 10th graders reflect the expected distribution of AFQ-Y scores in the target population.
- At baseline, the mean AFQ-Y score is 21.04 and the standard deviation is 13.01.
- We assume equal standard deviation for all samples/groups/arms.
- The correlation between AFQ-Y scores at baseline and AFQ-Y scores at T3 within patients is assumed to be large and to be the same across all arms. It is set to r = 0.80.

- All patients experience a decrease in their AFQ-Y score between baseline and T3 corresponding to a standardized effect size (Cohen's d) of -0.22 on average, as described in Van der Gucht et al. (2017).
- The difference in mean AFQ-Y score between TAU participants and web-based self-helped (WBSH) training participants at T3 corresponds to a standardized effect size (Cohen's d) of -0.50 (moderate effect).
- The difference in mean AFQ-Y score between TAU participants and web-based group (WBG) training participants at T3 corresponds to a standardized effect size (Cohen's d) of -0.80 (large effect).
- All groups are equal in size.
- The desired power is 80 % and the family-wise error rate is 5 %.

11.2.3 Method

The sample size is estimated based on simulations. In brief, the procedure went as follows:

1. we generated 9999 synthetic data sets for a range of potential samples sizes (from $n = 9$ to $n = 180$, in increments of 9) and effect sizes associated with the web-based self-helped training (0.4; 0.45; 0.5; 0.55) and the web-based group training (0.7; 0.75; 0.8; 0.85);
2. for each of the synthetic data sets, we computed a linear model examining the effects of the treatment conditions (TAU vs. pure self-help training; TAU vs. group training guided by a facilitator) on AFQ-Y scores at follow-up, accounting for baseline AFQ-Y scores;
3. the α level for significance of each null-hypothesis test is adjusted so as to keep the familiy-wise error rate $\leq 5\,\%$;
4. finally, the statistical power associated with a given sample size and a given pair of treatment effects on average AFQ-Y score is estimated as the proportion of tests with p-value $<$ p-adjusted across the simulated analyses.

Final Report

11.2.4 Alpha-correction for multiple testing

In the case when the trial is considered successful if both null hypotheses are rejected, no adjustment of the p-value for multiple testing is required ($\alpha = 0.05$). However, in the case when the trial is considered successful if at least one null hypothesis is rejected, the p-value for significance needs to be adjusted for multiple testing. We used the Bonferroni correction and the adjusted p-values was set to $\alpha = 0.025$.

11.2.5 Sample size estimates

Figures 1, 2, 3 and 4 show how the sample size depends on the power to be achieved and the expected effect sizes for the web-based self-helped training vs. TAU and the web-based group training vs. TAU. It is worth noting that all figures show almost vertical curves, illustrating, as expected, how sample size is sensitive to changes in the smaller effect (Self-help training

vs. TAU; range 0.40 - 0.55) and insensitive to changes in the larger effect (Group training vs. TAU; range: 0.70 - 0.85).

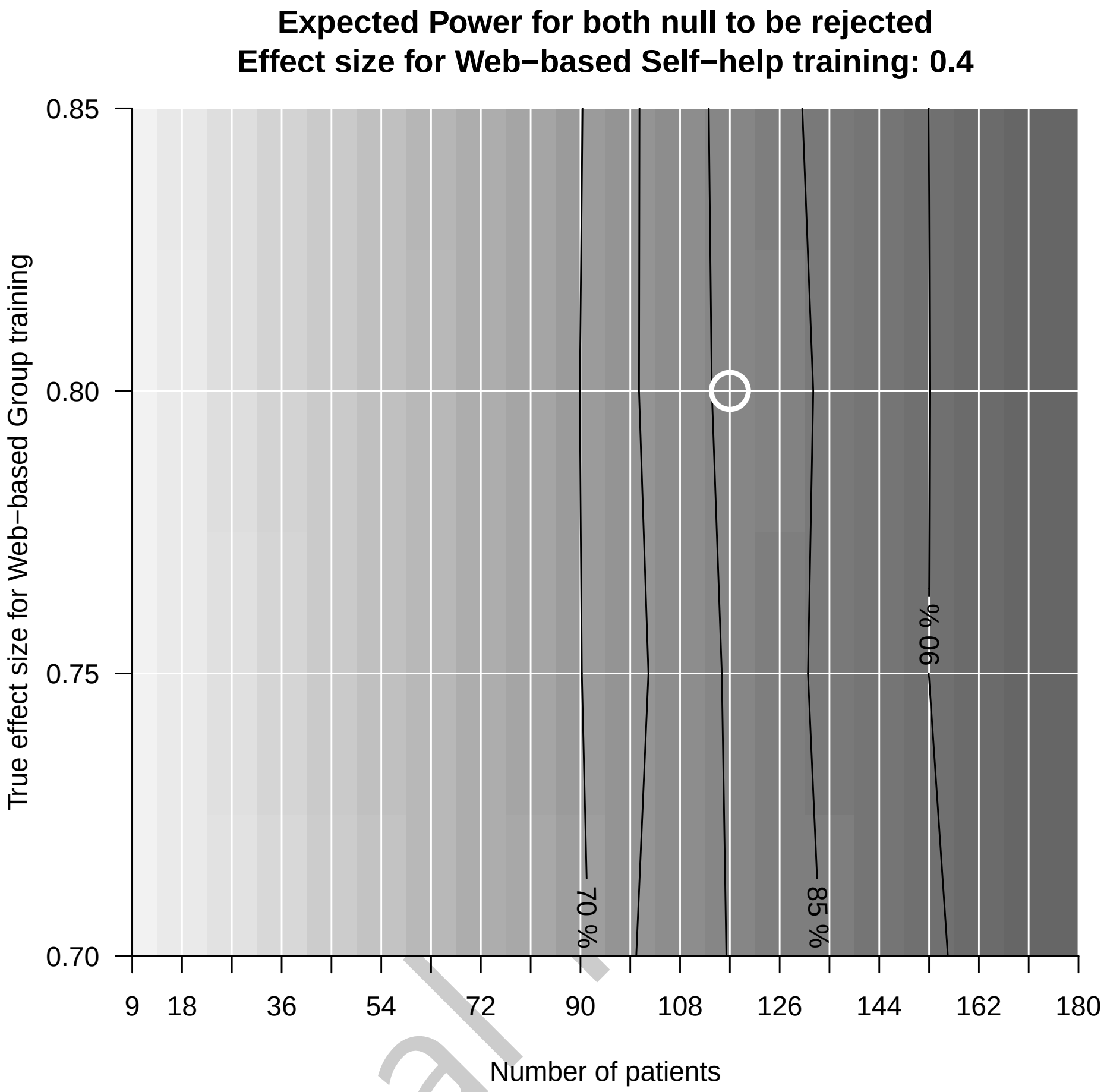

Figure 1: Simulated power at different combinations of effect sizes for the Web-based Group training and number of patients included in the study, assuming an effect size of 0.40 in the Web-based Self-help training arm.

Final

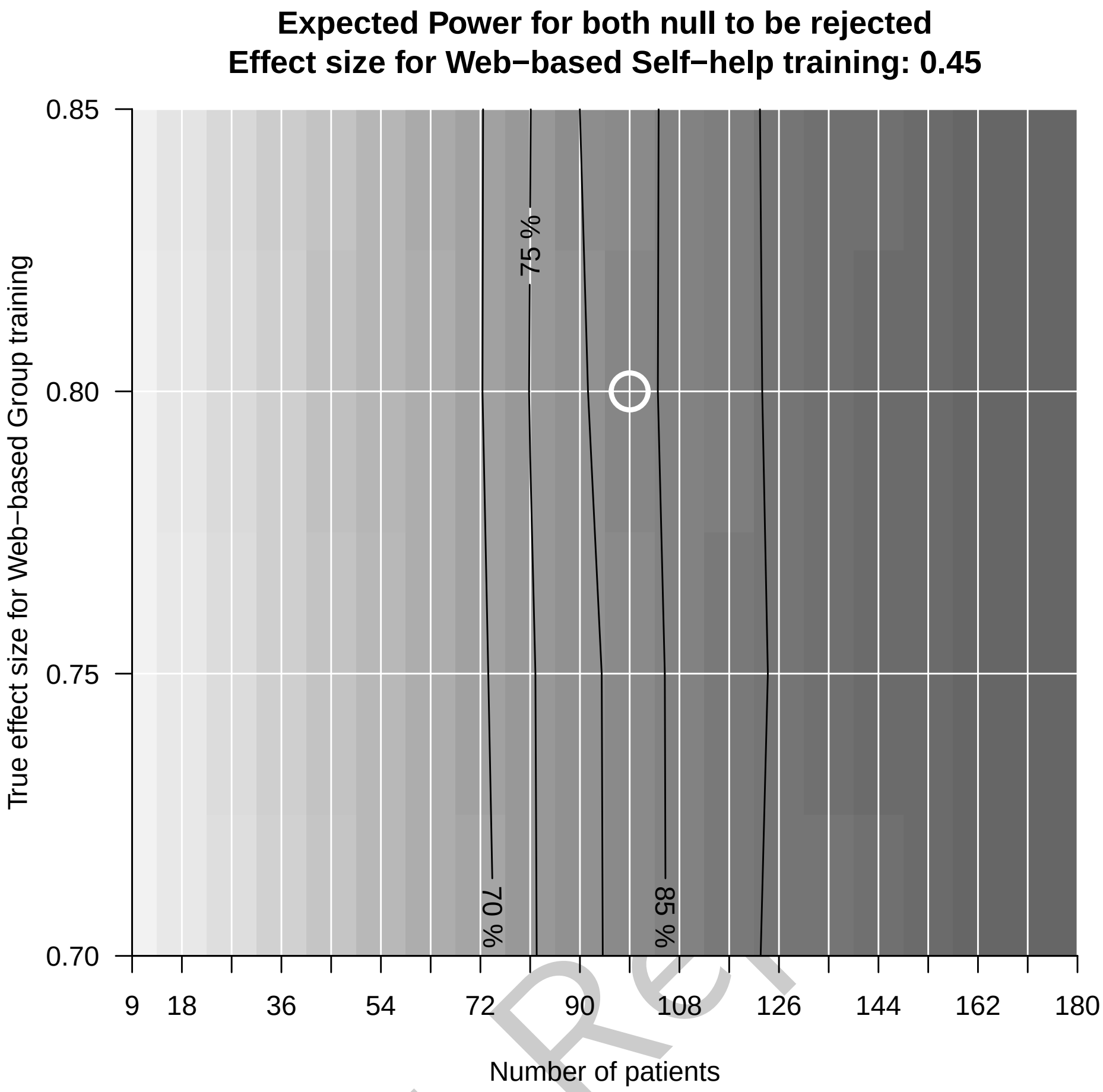

Figure 2: Simulated power at different combinations of effect sizes for the Web-based Group training and number of patients included in the study, assuming an effect size of 0.45 in the Web-based Self-help training arm.

Final Re

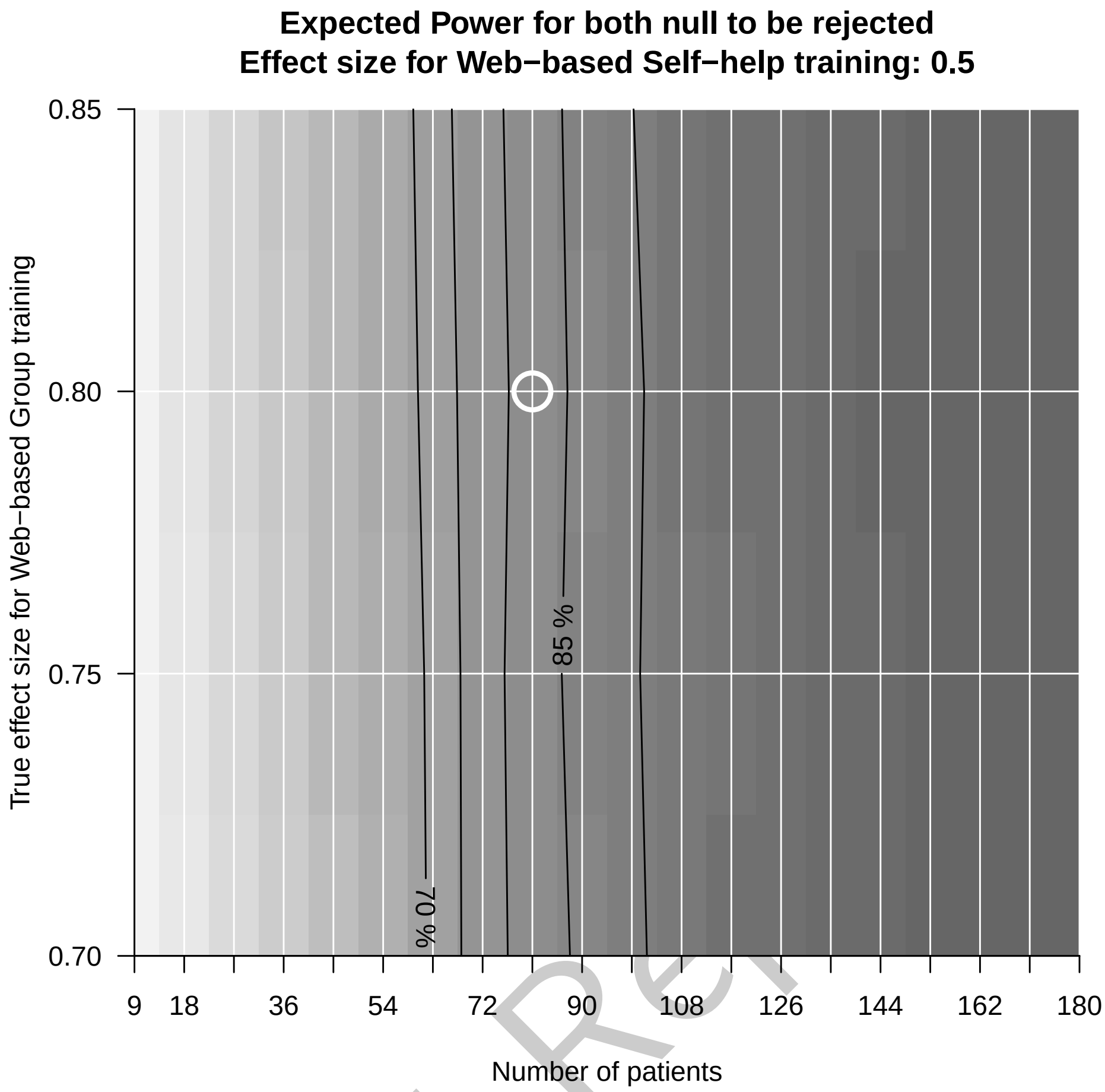

Figure 3: Simulated power at different combinations of effect sizes for the Web-based Group training and number of patients included in the study, assuming an effect size of 0.50 in the Web-based Self-help training arm.

Final Re

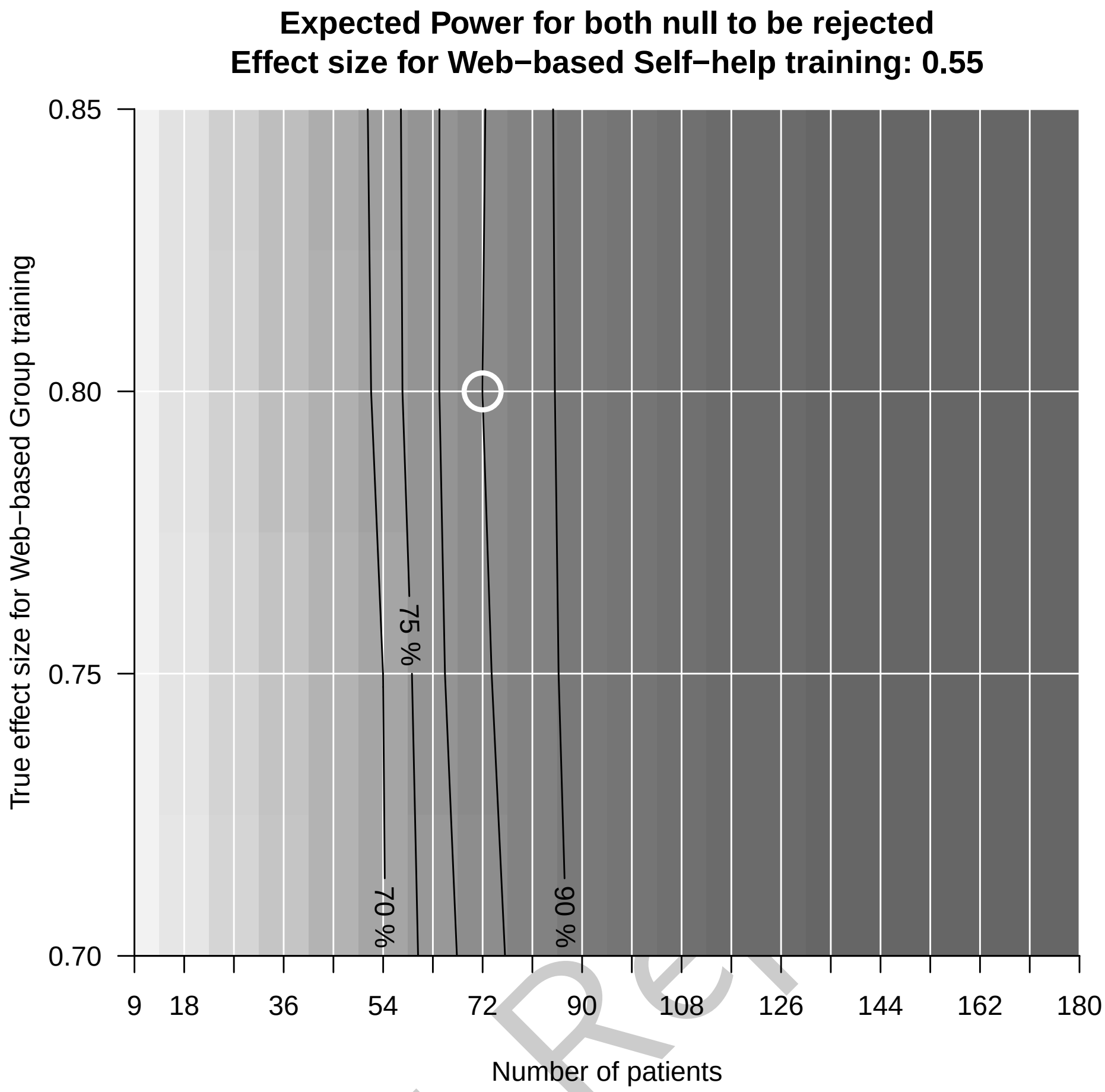

Figure 4: Simulated power at different combinations of effect sizes for the Web-based Group training and number of patients included in the study, assuming an effect size of 0.55 in the Web-based Self-help training arm.

Final Re

Based on the above-mentioned criteria and simulations, the sample size to reject both null hypotheses (web-based self-helped training vs. TAU *and* web-based group training vs. TAU) with 80 % power and 5 % family-wise error rate is $n = 78$ patients overall (i.e 26 patients per arm).

11.2.6 Correcting for the design effect

Since there will be several patients enrolled in each institutions and because the web-based group training guided by a facilitator results in cluster sampling, we need to adjust the sample size to the "design effect". In case of cluster sampling, the "design effect" can be computed as (Kish, 1965):

$D_{eff} = 1 + (n^* - 1)\rho$, where n^* is the size of the clusters and ρ is the intraclass correlation coefficient.

Then, the sample size can be calculated as:

$n = D_{eff} \times n_{eff}$, where n_{eff} is the effective sample size, i.e. the number of independent statistical units.

For the calculation, we assume that each center will enroll an average of 4 patients (realistic group size after discussions with multiple institutions) and the "effective" sample size should be 78 patients. We assume a weak resemblance in the AFQ-Y score of patients within the same group ($\rho = 0.2$). Based on the latter and further assuming a drop-out rate of 20 % based on previous experience, **150 patients in 38 groups** (i.e. 50 patients per arm) should be recruited.

11.3 Statistical criteria of termination of trial

None.

11.4 Planned analyses

11.4.1 Datasets to be analysed, analysis populations

The analysis will be conducted on the **full analysis set (FAS)** including all randomized patients according to intention-to-treat.

In case of deviation from protocol, e.g. patients not treated according to randomization, we will run a sensitivity analysis using the **per protocol set (PP)**.

11.4.2 Primary analysis

Linear mixed model with score at T3 and intervention and baseline score as factor and covariate respectively. The group identity in the group training arm will be included as a random factor to account for the clustering effect of the group training and the social structure of the institutions.

As a first approximation, and based on current knowledge of the social dynamics in residential youth institutions, the institution ID will be considered as negligible and will not be included in the model. However, a sensitivity analysis may be planned to test this assumption by including the institution ID as an additional random factor.

Similarly, the accuracy with which the group random effect is estimated and the accuracy of the estimates for the fixed effects may be tested by bootstrapping individuals within groups.

Statistical analyses will be conducted at the CTU Basel based on a pre-established report and analysis plan (RAP).

11.4.3 Secondary analyses

The secondary hypotheses described above will be analysed using linear mixed model with scores at T3 and intervention and baseline scores as factor and covariate respectively. The group identity in the group training arm will be included as a random factor to account for the clustering effect of the group training and the social structure of the institutions.

The effect of the moderators described above will be analysed using the same model as for the primary analysis, further including the moderator and its interaction with the treatment.

Final Report

11.4.4 Interim analyses

No interim analysis is planned.

11.4.5 Safety analysis

No safety analyses are planned.

11.4.6 Deviation(s) from the original statistical plan

If substantial deviations of the analysis as outlined in these sections are needed for whatever reason, the protocol will be amended. All deviations of the analysis from the protocol or from the detailed analysis plan will be listed and justified in a separate section of the final statistical report.

11.5 Handling of missing data and drop-outs

Will be determined at a later stage, when preparing the Report and Analysis Plan (RAP).

Final Report

References

Greco, L. A.; Lambert, W. & Baer, R. A. 2008: Psychological inflexibility in childhood and adolescence: development and evaluation of the avoidance and fusion questionnaire for youth. Psychological assessment 20(2):93.

Van der Gucht, K.; Griffith, J. W.; Hellemans, R.; Bockstaele, M.; Pascal-Claes, F. & Raes, F. 2017: Acceptance and commitment therapy (act) for adolescents: Outcomes of a large-sample, school-based, cluster-randomized controlled trial. Mindfulness 8(2):408–416.

Kish, L. 1965: Survey Sampling. John Wesley & Sons, New York.
