# Supplementary material for: Implementation and effectiveness of the web‑based skills training START NOW in adolescents in residential youth care: a cluster randomized controlled trial
Source: Child Adolesc Psychiatry Ment Health. 2026 Jun 29;20:131. doi: 10.1186/s13034-026-01127-z (PMC13591857; doi:10.1186/s13034-026-01127-z)
Supplement: Supplementary file 2 [file 13034_2026_1127_MOESM2_ESM.pdf]

Universität Basel

Departement Klinische Forschung

Universitätsspital Basel

Confirmed Final Report

Universität Basel, Departement Klinische Forschung, c/o Universitätsspital Basel, Spitalstrasse 12, 4031 Basel

Prof. Dr. Dr. Christina Stadler
Klinik für Kinder und Jugendliche der
Universitären Psychiatrischen Kliniken
Schanzenstrasse 13
4056 Basel

Basel, March 27, 2024

Report and analysis plan (RAP)

Study Title	Implementierung einer e-Version des Skillstrainings START NOW zur Förderung der Emotionsregulation und Resilienz in Institutionen des Massnahmenvollzugs
Internal DKF ID	pk19Stadler
Document Status	Confirmed Final Report

Contents

1 Background **3**

2 Objectives of the analysis **3**

2.1 Primary objective 3

2.2 Secondary objectives 4

2.3 Supplementary objectives 4

3 Study design **4**

4 Population and sample **5**

4.1 Inclusion criteria (population) 5

4.2 Exclusion criteria (sample) 5

Universität Basel
Departement Klinische Forschung
c/o Universitätsspital Basel
Spitalstrasse 12
4031 Basel, Switzerland

Nikki Rommers, PhD
Statistician, Department of Clinical Research
Telefon +41 328 77 14
nikki.rommers@usb.ch
dkf.unibas.ch

Confirmed Final Report

5 Estimands **6**

5.1 Target population 6

5.2 Potential intercurrent events 6

5.3 Strategies to account for intercurrent events 6

5.3.1 Primary estimand: Treatment policy strategy 6

5.3.2 Secondary estimand: While on treatment strategy 7

5.4 Analysis sets 7

6 Handling missing data **8**

7 General approach to the analysis **8**

8 Outcomes/endpoints **8**

8.1 Primary outcome and endpoint 8

8.2 Statistical hypothesis 9

8.3 Secondary outcomes 9

8.4 Safety outcomes 10

8.5 Additional variable definitions 10

9 Specific methods **10**

9.1 Analysis of the primary estimand: comparison of AFQ-Y in group training vs. TAU and self-helped training vs. TAU 10

9.2 Secondary Analyses 11

9.2.1 Primary estimand: comparison of AFQ-Y in group training vs. self-helped training 11

9.2.2 Primary estimand: secondary endpoints 11

9.2.3 Secondary estimand: AFQ-Y and secondary endpoints 11

9.2.4 Description of primary and secondary outcomes at other time points . . 12

9.2.5 Analysis of the caretakers 12

9.2.6 Supplementary analysis 12

9.3 Methods used to check assumptions of the main methods 12

9.4 Safety outcomes 12

9.5 Alternative methods 13

10 Sensitivity analyses **13**

References **13**

Confirmed Final Report

1 Background

Resilience is the ability to positively adapt to different life-situations. Resilient people are solution-oriented and show a high level of adaptability when faced with stress. They remain more confident about their future and find it easier to accept crises. Such competencies are valuable resources for psychological well-being that come with lower levels of anxiety and depression and are linked to successful life outcomes. Resilience can also mitigate the effects of early trauma on depressive symptoms.

A variety of resilience-oriented training programs have been developed to enhance resilience. One such approach is START NOW. START NOW is an evidence-based, integrative skills training program, which includes a manual with detailed instructions for group facilitation. The program combines aspects of cognitive behavioral therapy (CBT), motivational interviewing (MI), dialectical behavior therapy (DBT), acceptance and commitment Therapy (ACT) and trauma-sensitive care to improve general resilience by enhancing emotion and stress regulation skills.

Although START NOW is highly suited for youth displaying low levels of resilience placed in restrictive settings, some obstacles arise when implementing the program in residential youth care. Such obstacles are hard to overcome and limit a program's effectiveness.

A promising solution to the aforementioned implementation difficulties are web-based e-mental health approaches. E-mental health approaches are defined as "a primarily self-guided intervention programme that is executed by means of a prescriptive online programme operated through a website and used by consumers seeking health- and mental-health related assistance".

Pure web-based self-help training has been shown to be moderately effective. Web-based trainings including social support from an e-coach (guided e-learning) show greater effects and better adherence. With regard to dropout, dropout rates for e-mental health programs are lower when the intervention is provided with support as compared to when it is provided without support, suggesting that e-mental health programs with support are superior to those without.

2 Objectives of the analysis

This project aims to develop and evaluate a guided e-mental health intervention that could bridge the gap between the high prevalence of mental health problems in adolescents at risk and limited access to mental health programs and/or limited personal resources in youth welfare institutions. The study will investigate the efficacy of an e-START NOW approach combining a web-based application of START NOW and support by a trainer (online or face-to-face) on psychological flexibility.

2.1 Primary objective

The primary objective of this study is to investigate, using a randomized controlled trial, whether in a population of youth and young adults (aged 14-24) with AFQ-Y score ≥ 3.3 placed in residential youth institutions, web-based START NOW guided group training and web-based START NOW self-help training are more efficient than treatment as usual (TAU) in decreasing psychological *in*flexibility (primary endpoint) as measured by scores on the AFQ-Y questionnaire (primary outcome) 12 weeks $\pm$ 2 weeks (T3) after the end of the intervention.

2.2 Secondary objectives

Secondary objectives are:

1. Assess whether the web-based START NOW *guided group* training performs better than the web-based START NOW *self-helped* training in reducing psychological inflexibility as assessed by the AFQ-Y score.

2. Assess whether the web-based START NOW *guided group* training and web-based START NOW *self-helped* training results in enhanced general resilience, self-efficacy and well-being and in reduced general impairment, perceived stress, irritability, psychopathology, conduct and emotional problems compared to TAU.

3. Assess whether the web-based START NOW *guided group* training result in a greater enhancement of general resilience, self-efficacy and well-being, and in a greater reduction of general impairment, perceived stress, anger and irritability, psychopathology, conduct and emotional problems compared to web-based START NOW *self-helped* training.

4. Assess whether START NOW training courses for caretakers lead to enhanced resilience in caretakers compared to TAU.

5. Greater severity/comorbidity in terms of alcohol/drug use, anger-irritability and depressive-anxious symptoms (moderator, both subscales of the the MAYSI-2 score assessed at baseline) is expected to buffer the intervention's positive effect on resilience. We expect a potential effect on all outcomes.

2.3 Supplementary objectives

A supplementary objective is to explore the effect of caretaker variable on the treatment effect within each of the three interventions.

3 Study design

The study is a prospective multi-center 3-arm randomized controlled trial, where START NOW in the format of (1) guided web-based group training with a coach will be compared to (2) web-based self-help training and (3) a waitlist treatment as usual (TAU) control group.

After **baseline assessment (T1)** was done, 2 weeks before the start of the intervention, eligible participating centers were randomly assigned (n = 4-10 per group/institution) to one of the three conditions. The **intervention phase** lasted for 9 weeks.

A **post-treatment assessment (T2)** was conducted within 2 weeks after the end of the intervention.

A **first follow-up assessment (T3)** was done 12 weeks (± 2 weeks) after the end of the intervention.

A **second follow-up assessment (T4)** was done 24 weeks (± 2 weeks) after the end of the intervention.

Confirmed Final Report

Confirmed Final Report

The treatment (guided web-based vs. self-help training vs. TAU) was applied at the level of the institution so that all study participants in a given institution received the same treatment. The reason for this is that staff members (caretakers) of the institutions/centers subjected to condition (1) will receive a two-day training in START NOW, with potential "spillover" effects since such training may modify their every-day practice.

The design produces two sources of clustering: 1) the center/institution; 2) the training group for study participants in the "guided web-based" arm. In centers/institutions assigned to the "self-helped" training, the center is the relevant clustering level. In small institution assigned to the "group-training" arm, with only 1 "group-training" group, the center and the group are confounded and either the center or the group, or any labelling combining the two, becomes the relevant clustering level. In larger institution assigned to the "group-training" arm with capacities to organize >1 "group-training" groups, the group level could potentially add on top of the center level. However, in large institutions groups can be considered independent because they are cared for by different staff members, because members of different groups never meet and because groups have their own social dynamics. Consequently, in such large institutions the groups within the center are the relevant clustering levels. To sum up, for "TAU" and "self-helped" arms the institution is the clustering factor; for the "guided/group training" arm the group is the clustering factor.

4 Population and sample

Participants will include study participants who give their consent to participate and fulfil the following inclusion/exclusion criteria:

4.1 Inclusion criteria (population)

- Youth and young adults in RYC or correctional institutions;
- Age of 14 to 24 years;
- Need for improvement: AFQ-Y equal or higher than 34.05, or MAYSI-2 subscale Angry-Irritable equal or higher than 5, or MAYSI-2 subscale Depressed-Anxious equal or higher than 3;
- Sufficient speaking, writing and reading skill in German or French (written) informed consent;
- No planned discharge from institution within the 12-week intervention period.

4.2 Exclusion criteria (sample)

- Acute psychotic symptoms or mania;
- Suicidality;
- Concurrent group based psychotherapeutic treatment similar to START NOW.

Confirmed Final Report

5 Estimands

The estimand describes what is to be estimated based on the question of interest. In this study estimands are defined based on pre-defined potential intercurrent events and aim at addressing the question of the effect of web-based group training and web-based self-helped training vs. TAU on psychological flexibility in different relevant populations and according to different treatment strategies. To this end, we identify the target population and the potential intercurrent events below.

5.1 Target population

Youth and young adults in RYC or correctional institutions aged 14 to 24 years with need for improvement of resilience, with no acute psychotic symptoms or mania, or suicidality.

5.2 Potential intercurrent events

An intercurrent event is as an event that occurs after the study intervention has been initiated and which either precludes the observation of the outcome variable or affects its measurement or interpretation.

- Post-treatment (T3) measure of the primary outcome is missing because the participant left the institution or was placed in another institution;
- Post-treatment (T3) measure of the primary outcome is missing because the participant withdrew consent - study takes too much time/effort, subject unwilling to engage with diagnostic interview/questionnaires;
- Participants complied to the treatment for some weeks, but then stopped (e.g. followed a web-based training (self-helped or group) in the first 7 sessions and did not do any training afterwards);
- Participants irregularly complied with the treatment (e.g. followed a web-based training (self-helped or group) every second week only).

5.3 Strategies to account for intercurrent events

5.3.1 Primary estimand: Treatment policy strategy

In the target population, non-adherence or intermittent adherence to the protocol may be expected due to the very nature of the psychological difficulties suffered by the target study participants. Moreover, self-help web-based e-mental programmes are known to show moderate effect, which can at least be partly due to lower compliance with the treatment (e.g. the training is done, but not seriously and thoroughly enough to trigger an effect). In such context, the professionals seeking to provide care to young adults as well as the justice institutions may be interested in assessing the effect of the web-based guided-group and self-helped START NOW programme under conditions as close to that encountered in the "real life" as possible.

Confirmed Final Report

Consequently, we chose to assess the primary and secondary objectives using a treatment policy strategy that considers intercurrent events as part of the treatment and thus estimates the effect of the treatment regardless of treatment discontinuation. To this end, and although the effects estimates of the guided-group and self-helped START NOW may be lower than if assessed on a population of "compliers", all primary and secondary objectives will be assessed using the full analysis set as defined according to intention-to-treat and comprising all participants with baseline values for the focal primary or secondary endpoint. Missing values post-treatment (T3) will be imputed using multiple imputation according to Rubin (Rubin, 1987) (see below).

5.3.2 Secondary estimand: While on treatment strategy

Although the "treatment policy strategy" likely allows a realistic estimation of the treatment effectiveness in real life accounting for the fact that motivation may vary substantially across participants thus generating various levels of compliance with the treatment, it may be interesting to assess the effect of the treatment in a population of more motivated, more compliant participants. Indeed, if treatment estimates are higher in "compliers" compared to those obtained using the "treatment policy strategy" it may be wise to implement incentives to increase compliance, such as rewards when adherence reaches a certain threshold. In this study, participants in the group-training and the self-helped arms were scheduled to follow at least one training session every week over 9 weeks. Based on such schedule, we chose to define **compliance with the treatment as attendance to at least 50 % of the group-training sessions or completion of at least 50 % of the self-helped web-based programme** Compliance is met when at least 5 out of 9 sessions are completed or attended. Participants in the group training arm or the self-helped training arm failing to fulfil these requirements will be considered as non compliers.

The effects of the treatment (web-based group training & web-based self-helped training vs. TAU) in the population of more motivated participants will be estimated using a data set including all eligible participants with baseline value for the focal endpoint and considered compliers according to the definition above.

Missing values post-treatment (T3) will be imputed using multiple imputation according to Rubin (Rubin, 1987) (see below).

5.4 Analysis sets

To asses the "treatment policy strategy" estimand we will use the full analysis set (FAS) comprising all eligible study participants with baseline measure of the focal endpoint and according to the intention to treat.

To asses the "While on treatment strategy" estimand we will use a set of data, hereafter referred to as the While-on-treatment-strategy set (WoTSS), comprising all eligible study participants with baseline measure of the focal endpoint and considered compliers according to the definition above.

The safety analysis set will consist of all study participants who received at least one treatment session, meaning that the patients were exposed to one of the interventions.

Additionally, we have the caretaker data set including information on the caretakers. This data set will be used for the objectives that are related to the caretakers.

Confirmed Final Report

6 Handling missing data

One potential reason why the primary or the secondary outcomes (at T3 post-treatment) may be missing is the possibility that participants with lower levels of resilience at follow-up withdraw their consent and refuse to be further evaluated. In such a case the value is missing not at random, because the probability of missingness is linked to the hypothetical value. However, missing at random condition may be restored if baseline values and values measured at T2 and T4 are predictors of the outcome at T3 and/or if other non-missing auxiliary variables are good predictors of post-treatment T2 values (National Research Council, 2010).

We will use multiple imputation to address the issue of missing outcome values for both primary and secondary endpoints. Missing values will be imputed using Multivariate Imputation by Chained Equations (MICE) with relevant predictor variables and baseline values and potentially values at T3 and T4 of the focal endpoint. The imputation method will account for the clustering of the data - data are clustered by institutions/groups. One hundred (100) datasets will be imputed and used to model the effect of web-based group training and web-based self-helped training vs. TAU on the focal endpoint. The results of the 100 models will be pooled using Rubin's formula (Rubin, 1987). The procedure will be applied in the same way for all primary and secondary endpoints.

7 General approach to the analysis

We will use linear mixed-effect models to analyse all primary and secondary endpoints. The identifier of the institution-group (for institutions with more >1 treated group) and of the institution (for those with only 1 treated group) will be included as a random intercept. The treatment (web-based group training, web-based self-helped training or TAU) will be included as a fixed effect. Baseline measures for the focal endpoint will be included as a covariate.

All analyses will be performed using `R version 4.3.1 (2023-06-16 ucrt)` or higher (R Core Team, 2023).

8 Outcomes/endpoints

8.1 Primary outcome and endpoint

The primary outcome is psychological *in*flexibility as measured by the Avoidance and Fusion Questionnaire for Youth (AFQ-Y). The AFQ-Y is a validated self-rating questionnaire for assessing psychological inflexibility in youth and young adults. Participants answer 17 items on a 5-point Likert scale. Total score ranges from 0 to 68. *Higher* total scores indicate *lower* psychological flexibility.

The primary outcome is measured at baseline 2 weeks before the start of the intervention (T1), 2 weeks after the end of the intervention (T2), 12 weeks after the end of intervention (T3) and 24 weeks after the end of intervention (T4).

The primary endpoint is the change in psychological inflexibility from baseline (T1) to post-treatment (T3). It is assessed as the post-treatment score in the AFQ-Y accounting for baseline score.

Confirmed Final Report

8.2 Statistical hypothesis

The primary objective leads to formulating the following null and alternative hypotheses.

Web-based *self-helped* training vs. TAU

- H_0: 12 weeks after the end of the intervention (at T3), mean inflexibility, as assessed by the Avoidance and Fusion Questionnaire for Youth (AFQ-Y), is the **same** in participants receiving treatment as usual (TAU) and in participants receiving a web-based START NOW self-helped training.
- H_1: 12 weeks after the end of the intervention (at T3), mean inflexibility, as assessed by the AFQ-Y, is **not the same** in participants receiving treatment as usual (TAU) and in in participants receiving a web-based START NOW self-helped training.

Web-based *group* training vs. TAU

- H_0: 12 weeks after the end of the intervention (at T3), mean inflexibility, as assessed by the AFQ-Y, is the **same** in participants receiving treatment as usual (TAU) and in participants receiving a web-based START NOW group training .
- H_1: 12 weeks after the end of the intervention (at T3), mean inflexibility, as assessed by the AFQ-Y, is **not the same** in participants receiving treatment as usual (TAU) and in participants receiving a web-based START NOW group training.

8.3 Secondary outcomes

All secondary outcomes will be assessed at baseline 2 weeks before the start of the intervention (T1), 2 weeks after the end of the intervention (T2), 12 weeks after the end of intervention (T3) and 24 weeks after the end of intervention (T4).

1. General resilience: Total score on the German version of the Connor-Davidson Resilience Scale for both participant and caretaker (CD-RISC). 10-item version with 5-point Likert-scales (0-4); total score ranges from 0 to 40. *Higher* scores indicate *greater* resilience.
2. Psychological well-being: Total score on the World Health Organization Well-Being Index (WHO-5). 5 items on 6-point Likert-scales (0-5); total score ranges from 0 to 25. *Higher* scores indicate *higher* well-being.
3. Self-efficacy: Total score on the German "General Self-Efficacy Scale" (Skala zur Allgemeinen Selbstwirksamkeitserwartung, SWE). 10 items on 4-point Likert-scales (1-4). Total score ranges from 10 to 40. *Higher* scores indicate *higher* convictions on subjective controllability or competence expectations.
4. General impairment: All sub scores and total score on Columbia Impairment Scale (CIS), both in self-rating and external rating. 13 items on 5-point Likert-scales (0-4). Score ranges from 0 to 52. *Higher* scores indicate *greater* impairment.
5. Perceived stress: Total score on Perceived Stress Scale (PSS-4). 4 items on 5-point Likert-scales. Score ranges from 0 to 16. *Higher* scores indicate *higher* stress.

Confirmed Final Report

6. Anger-irritability: Total score on Affective Reactivity Scale. 7 items on 3-point Likert scales (0-2). Only the 6 first items are summed up into a total score that ranges from 0 to 12. The 7th item is analyzed separately. *Higher* scores indicate *higher* irritability.
7. Emotional and behavioral symptoms: All sub scores and total score on the Strength and Difficulties Questionnaire (SDQ). 25 items on 3-point Likert-scales (0-2). Total score ranges from 0 to 50. *Higher* scores indicate *greater* emotional and behavioural difficulties.
8. Participants within the two intervention conditions will rate their satisfaction with the training using the Client Satisfaction Questionnaire (CSQ), specifically developed to assess a participant's experience and satisfaction with START NOW. 8 items on 4-point Likert-Scales (0-3); response options are provided with smileys; two open questions about what participants liked or would want to be changed within the training.

8.4 Safety outcomes

All possible adverse and serious adverse events will be recorded during and after the intervention.

8.5 Additional variable definitions

The moderators of interest, angry-irritable symptoms and depressed-anxious symptoms are assessed via the Massachusetts Youth Screening Instrument-2 (MAYSI-2) questionnaires. These are both continuous scores.

The following covariates will be included in all models:

- Age
- Sex
- MAYSI-2 score (psychopathology score)
- Experience of the caretaker with Start Now programmes

9 Specific methods

9.1 Analysis of the primary estimand: comparison of AFQ-Y in group training vs. TAU and self-helped training vs. TAU

This analysis will use the FAS. We will use a linear mixed-effect model assuming normal distribution of the error with the post-treatment AFQ-Y as the dependent variable and the treatment as the fixed factor, the institution-group identifier as the random intercept and baseline AFQ-Y values as a covariate, in addition to the covariates listed above. The TAU arm will be set as the reference level to allow direct comparison of the effectiveness of the group training method and the self-helped training with the TAU. The effectiveness of the intervention groups will be assessed based on their estimates and the associated 95 % confidence intervals and p-values.

Confirmed Final Report

9.2 Secondary Analyses

9.2.1 Primary estimand: comparison of AFQ-Y in group training vs. self-helped training

This analysis will use the FAS. The same model as above will be used, but the self-helped training arm will be defined as the reference level. Any difference between the self-helped training arm and the group training arm will be examined 2-sided using the corresponding estimate and the associated 95 % confidence interval and p-value.

It must be noted that secondary analyses are exploratory in nature and that results have thus to be interpreted as hypothesis generating and not as confirmatory. No correction for multiple testing is planned. Therefore, P-values should be interpreted as a continuous measure of evidence against the corresponding null-hypothesis (i.e. no association with predictor) and not as confirmatory ("significant" vs. "non-significant").

9.2.2 Primary estimand: secondary endpoints

These analyses will use the FAS. For each of the secondary endpoints, we will fit a linear mixed-effect model assuming normal distribution of the error with the T3 score of the focal endpoint as the dependent variable and the treatment as the fixed factor, the institution-group identifier as the random factor and baseline endpoint values as a covariate. All analyses will additionally be adjusted for the same pre-defined set of covariates. The TAU arm will be set as the reference level to allow direct comparison of the effectiveness of the group training method and the self-helped training with the TAU. The comparison between the self-helped training and the group training will be made by defining the self-helped training arm as the reference level. We will analyse all secondary endpoint for the short term effect (T1 to T3), and the AFQ-Y for the long term effect (24 weeks). The effectiveness of the intervention groups will be assessed based on their estimates and the associated 95 % confidence intervals and p-values.

In the secondary endpoints that involve a moderator, this particular moderator will be added to the model as an interaction term with the effect of interest (i.e. the group effect). Each model only included one moderator.

9.2.3 Secondary estimand: AFQ-Y and secondary endpoints

These analyses will use the WoTSS. The T3 scores of all endpoints will be compared across treatment arms using linear mixed-effect models assuming normal distribution of the error with the treatment as a fixed factor, the institution-group as a random intercept and the baseline endpoint value as a covariate. All analyses will additionally be adjusted for the same pre-defined set of covariates. We will present the estimates and the associated 95 % confidence intervals and p-values for the fixed effects.

The comparison between TAU and the self-helped training and the group training will be made by defining the TAU arm as the reference level. The comparison between the self-helped training and the group training will be made by defining the self-helped training arm as the reference level.

Confirmed Final Report

9.2.4 Description of primary and secondary outcomes at other time points

The primary and secondary analysis only included the change from baseline to T3. We will describe and visualise the primary and secondary outcomes at each of the time points, stratified by treatment group.

9.2.5 Analysis of the caretakers

We will compare the resilience level of the caretakers between the ones who received the START NOW training and the ones who did not with an independent samples t-test in case of normally distributed resilience scores, and with the Wilcoxon signed-rank test in case the scores are not normally distributed. This analysis will be performed in the caretaker data set.

9.2.6 Supplementary analysis

We will assess the association between the caretaker variables, such as previous experience with a START NOW program, working experience, and characteristics of the working circumstances in the centre on the treatment effect regarding the primary outcome within each of the treatment groups (i.e. START NOW guided group training, START NOW self-helped training, and TAU).

As a secondary supplementary analysis, we will assess whether the treatment effect depends on the following baseline participant characteristics:

- Duration of institutionalization
- Age
- Diagnosis of psychological disease
- Previous stay in institution or care family

We will assess the association between these covariable and the primary endpoint, as well as the potential dependency on treatment, using mixed effects linear regression models. These models will be similar to the primary analysis with the AFQ-Y score at T3 as the outcome, baseline AFQ-Y score as covariate, and the intervention by characteristic interaction effect as fixed effect of interest. A separate model will be fit for each of the baseline characteristics.

9.3 Methods used to check assumptions of the main methods

Assumptions of linearity, normal distribution of the error and normal distribution of the random effect will be assessed using standard visual tools (e.g. residuals vs. predicted values, QQ-plots, etc.).

9.4 Safety outcomes

Safety outcomes will be described in the safety data set.

Confirmed Final Report

9.5 Alternative methods

If the assumptions of a normal distribution of the error does not hold, potentially due to a floor and/or ceiling effect of the outcome questionnaires, we will fit a tobit model instead of the planned linear regression models. These tobit models will have the same predictor(s), outcomes and random effects included as the above-described models. The general idea of tobit regression is that it models both the probability of reaching either the floor or ceiling of the questionnaire, and the development over time between the floor and ceiling.

10 Sensitivity analyses

A sensitivity analysis for the treatment policy strategy will be done by using a complete case set (no imputation) comprising study participants with non-missing baseline and follow-up (T3) values for the AFQ-Y score and fitting a linear mixed-effect model with the AFQ-Y score as the dependent variable, the treatment as a fixed effect, an identifier of the institution-group as a random effect and the baseline AFQ-Y scores as a covariate.

References

National Research Council. 2010: The Prevention and Treatment of Missing Data in Clinical Trials. Washington, DC: The National Academies Press. https://doi.org/10.17226/12955. The National Academies Press, Washington DC.

R Core Team. 2023: R: A Language and Environment for Statistical Computing. R Foundation for Statistical Computing.

Rubin, D. B. 1987: Multiple Imputation for Nonresponse in Surveys. John Wiley & Sons.
