# Supplementary material for: Implementation and effectiveness of the web‑based skills training START NOW in adolescents in residential youth care: a cluster randomized controlled trial
Source: Child Adolesc Psychiatry Ment Health. 2026 Jun 29;20:131. doi: 10.1186/s13034-026-01127-z (PMC13591857; doi:10.1186/s13034-026-01127-z)
Supplement: Supplementary file 3 [file 13034_2026_1127_MOESM3_ESM.pdf]

Universität Basel
Departement
Klinische Forschung

Universitätsspital Basel

Universität Basel, Departement Klinische Forschung, c/o Universitätsspital Basel, Spitalstrasse 12, 4031 Basel

Prof. Dr. Dr. Christina Stadler
Klinik für Kinder und Jugendliche der
Universitären Psychiatrischen Kliniken
Schanzenstrasse 13
4056 Basel

Final Report

Basel, November 21, 2024

Methods & Statistics

Study Title	Implementierung einer e-Version des Skillstrainings START NOW zur Förderung der Emotionsregulation und Resilienz in Institutionen des Massnahmenvollzugs
Internal DKF ID	pk19Stadler
Document Status	Final Report

Summary

This study aimed to compare a group-based and self-guided (i.e., e-learning) START NOW program and treatment as usual (TAU) in institutionalized youngsters across different centres in Switzerland. The main outcome was the AFQ questionnaire, assessing the resilience level, 12 weeks after the end of the intervention.

Although dropout rates did not differ largely between the three groups, a first important finding of the study is that the number of compliers is much larger in the group-based training group compared to the self-guided training group with a median of 10 (IQR 8-12) sessions attended in the group-based training group, compared to a median of 0 (IQR 0-6) sessions attended in the self-guided training group. This means that the group guidance is necessary to make the youngsters attend the training sessions. This imbalance in number of sessions attend also had consequences for the per protocol population, including only participants who fulfilled the complier criteria. In the self-guided training group, 26.7% of participants were considered compliers, compared to 81.0% of participant in the group-based group.

No differences between the groups after the intervention were found in the full analysis set, or in the per protocol data set. The secondary outcomes, consisting of questionnaires assessing different constructs, did not show any between-group differences. Based on these

Universität Basel
Departement Klinische Forschung
c/o Universitätsspital Basel
Spitalstrasse 12
4031 Basel, Switzerland

Nikki Rommers, PhD
Statistician, Department of Clinical Research
Telefon +41 328 77 14
nikki.rommers@usb.ch
dkf.unibas.ch

findings, we can conclude that in this setting with very low compliance rates of self-guided training, START NOW did not result in any differences in resiliance of any of the secondary endpoints compared to TAU, and no differences were seen between the group-based and self-guided START NOW training.

Final Report

Contents

1 Background **5**

2 Objectives of the analysis **5**

2.1 Primary objective . . . 5

2.2 Secondary objectives . . . 6

2.3 Supplementary objectives . . . 6

3 Data source and analysis sets **6**

3.1 Population and analysis set . . . 6

4 Methods **9**

4.1 Imputations . . . 9

4.1.1 Specific methods . . . 10

4.1.2 Answer option "do not want to disclose" . . . 11

4.2 Primary and secondary analyses . . . 11

4.3 Caretaker analysis . . . 11

4.4 Supplementary analysis . . . 12

5 Results **13**

5.1 Descriptive statistics . . . 13

5.1.1 Participant demographics . . . 14

5.1.2 Caretakers . . . 18

5.1.3 Questionnaire data over time . . . 19

5.2 Primary analysis . . . 22

5.3 Secondary analyses . . . 23

5.3.1 Secondary endpoints . . . 23

5.3.2 Long term effect AFQ . . . 25

5.4 Secondary estimand: per protocol analysis . . . 26

5.5 Supplementary analysis . . . 28

5.6 Safety analysis . . . 29

5.7 Sensitivity analysis . . . 30

5.7.1 Complete cases . . . 30

5.7.2 Dropout . . . 30

5.7.3 Compliers . . . 33

Final Report

6 Supplementary tables 35
6.1 Full tables of the secondary endpoints 35
6.1.1 TAU as reference 35
6.1.2 Self-guided training as reference 39
6.2 Full tables of the secondary estimand: per protocol analysis 39
6.2.1 TAU as reference 39
6.2.2 Self-guided training as reference 47
6.2.3 Supplementary analysis: interactions with baseline particpant characteristics 52
7 Deviations from report and analysis plan (RAP) 54
References 54

Final Report

Final Report

1 Background

Resilience is the ability to positively adapt to different life situations. Resilient people are solution-oriented and show a high level of adaptability when faced with stress. They remain more confident about their future and find it easier to accept crises. Such competencies are valuable resources for psychological well-being that come with lower levels of anxiety and depression and are linked to successful life outcomes. Resilience can also mitigate the effects of early trauma on depressive symptoms.

A variety of resilience-oriented training programs have been developed to enhance resilience. One such approach is START NOW. START NOW is an evidence-based, integrative skills training program, which includes a manual with detailed instructions for group facilitation. The program combines aspects of cognitive behavioural therapy (CBT), motivational interviewing (MI), dialectical behaviour therapy (DBT), acceptance and commitment Therapy (ACT) and trauma-sensitive care to improve general resilience by enhancing emotion and stress regulation skills.

Although START NOW is highly suited for youth displaying low levels of resilience placed in restrictive settings, some obstacles arise when implementing the program in residential youth care. Such obstacles are hard to overcome and limit a program's effectiveness.

A promising solution to the aforementioned implementation difficulties are web-based e-mental health approaches. E-mental health approaches are defined as "a primarily self-guided intervention programme that is executed by means of a prescriptive online programme operated through a website and used by consumers seeking health- and mental-health related assistance".

Pure web-based self-help training has been shown to be moderately effective. Web-based trainings including social support from an e-coach (guided e-learning) show greater effects and better adherence. With regard to dropout, dropout rates for e-mental health programs are lower when the intervention is provided with support as compared to when it is provided without support, suggesting that e-mental health programs with support are superior to those without.

2 Objectives of the analysis

The main aim of the analysis was to assess whether there is a different effect of a group-based START NOW programme compared to a self-guided START NOW programme and treatment as usual (TAU) on resilience in youth. Resilience is evaluated with the AFQ-questionnaire 12 $\pm$ 2 weeks after the end of the intervention.

2.1 Primary objective

The primary objective of this study is to investigate, using a randomized controlled trial, whether in a population of youth and young adults (aged 14-24) with AFQ-Y score $\geq$ 3.3 placed in residential youth institutions, web-based START NOW guided group training and web-based START NOW self-guided training are more efficient than treatment as usual (TAU) in decreasing psychological *in*flexibility (primary endpoint) as measured by scores on the AFQ-Y questionnaire (primary outcome) 12 weeks $\pm$ 2 weeks (T3) after the end of the intervention.

Final Report

2.2 Secondary objectives

Secondary objectives are:

1. Assess whether the web-based START NOW *guided group* training performs better than the web-based START NOW *self-helped* training in reducing psychological inflexibility as assessed by the AFQ-Y score.
2. Assess whether the web-based START NOW *guided group* training and web-based START NOW *self-helped* training results in enhanced general resilience, self-efficacy and well-being and in reduced general impairment, perceived stress, irritability, psychopathology, conduct and emotional problems compared to TAU.
3. Assess whether the web-based START NOW *guided group* training result in a greater enhancement of general resilience, self-efficacy and well-being, and in a greater reduction of general impairment, perceived stress, anger and irritability, psychopathology, conduct and emotional problems compared to web-based START NOW *self-helped* training.
4. Assess whether START NOW training courses for caretakers lead to enhanced resilience in caretakers compared to TAU.
5. Greater severity/comorbidity in terms of alcohol/drug use, anger-irritability and depressive-anxious symptoms (moderator, both subscales of the the MAYSI-2 score assessed at baseline) is expected to buffer the intervention's positive effect on resilience. We expect a potential effect on all outcomes.

2.3 Supplementary objectives

A supplementary objective is to explore the effect of caretaker variables on the treatment effect within each of the three interventions. Unfortunately, the available data did not allow us to perform detailed caretaker analyses.[1]

3 Data source and analysis sets

The data were prepared in a separate data management (DM) file: `pk19StadlerDM001_-main.pdf`.

3.1 Population and analysis set

For the primary analysis following the treatment policy strategy estimand, in which we analyse the data according to the intention to treat principles, the full analysis set (FAS) was used. This analysis set consists of all eligible participants with a baseline (screening) measure of the AFQ questionnaire as well as valid data for the baseline covariates for which the analysis was adjusted. Using these criteria, a total of 164 participants were included in this analysis. The median number of sessions participated in per group, out of the total of 12 sessions, is displayed in Table 1.

Group	Median [Q1, Q3]
group	10 [8, 12]
self	0 [0, 6]

Table 1: Overview of the median [Q1, Q3] number of sessions in each of the training groups

None of the participants had missing data in the baseline covariates (screening visit), and all eligible and randomized participants who provided informed consent were included in the analyses. During the course of the study, some participants dropped out. An overview of the number of drop-outs per group is provided in Table 2.

	No dropout	Dropout
group	53 (35.33 %)	5 (35.71 %)
self	53 (35.33 %)	7 (50 %)
TAU	44 (29.33 %)	2 (14.29 %)

Table 2: Overview of the total number of dropouts (with percentage) by training group

For the **per protocol data set**, we included all participants who fulfilled the complier criteria as described in the protocol. We first assessed the total number of sessions attended by the patients in each of the intervention groups. The numbers are shown in Table 3. For TAU, no sessions were defined; thus, the concept of compliance is not applicable to this group and therefore all participants are considered compliers. An overview of the number and percentage of compliers in each of the groups is presented in Table 4. It must be noted that the number of compliers is much larger in the group-based training group compared to the self-guided training group. This leads to unequal group sizes in the per protocol analysis, and might be an important consideration in this study by itself. Looking at the number of sessions attended, we immediately see that in the self-guided training group, more than half of the participants did not attend a single session. This is in sharp contrast to the group-based training group in which more than half of the participants attended at least 10 sessions.

	group	self
0	0 (0 %)	27 (50.94 %)
1	1 (1.85 %)	4 (7.55 %)
2	0 (0 %)	1 (1.89 %)
3	2 (3.7 %)	2 (3.77 %)
4	3 (5.56 %)	3 (5.66 %)
5	1 (1.85 %)	0 (0 %)
6	5 (9.26 %)	3 (5.66 %)
7	0 (0 %)	2 (3.77 %)
8	10 (18.52 %)	2 (3.77 %)
9	4 (7.41 %)	1 (1.89 %)
10	4 (7.41 %)	0 (0 %)
11	4 (7.41 %)	0 (0 %)
12	20 (37.04 %)	8 (15.09 %)

Table 3: Overview of the total number of sessions attended by the participant in the group- and self-guided training groups. The rows represent the total number of attended sessions, with for each number of sessions the number of participants (with percentage)

Final Report

	Non-complier	Complier
group	11 (20 %)	47 (43.12 %)
self	44 (80 %)	16 (14.68 %)
TAU	0 (0 %)	46 (42.2 %)

Table 4: Overview of the total number of compliers (with percentage) by training group.

Final Report

4 Methods

The main methods are explained in the report and analysis plan of this project and will not be repeated here. Only changes or more detailed information is reported here.

All analyses were performed in R version 4.4.1 (R Core Team, 2023).

In this report we show uncorrected p-values for each of the analyses. Given the large number of analyses performed, it must be noted that only the p-values of the primary analysis, for which the study was powered, should be interpreted as confirmatory. All other p-values are explorative and should be interpreted as continuous and with caution due to the large number of tests performed.

Final Report

4.1 Imputations

The main analysis plan states that multiple imputations will be performed under the assumption that the data are missing at random. These methods were used, however, since we are mainly dealing with questionnaire scores, consisting of multiple items, specific considerations were made.

Missing data were a main issue in this analysis, with the proportion of missing data at T3, the time point of the outcome measures, being as over 50% for all questionnaires (see Table 5. assessed. This leads to a high variance when imputing the data and requires sensitivity analyses to get an idea of the profile of the participants who did not fill in the questionnaires. With this proportion of missing data, it is questionable what the imputations bring, apart from increasing the sample size of the analysis allowing for more covariates to be included.

Variable	t1	t2	t3	t4
AFQ total	17.28	62.75	56.94	69.81
CD-RISC total	33.33	70.59	65.97	74.21
CD-RISC total caretaker	14.81	38.56	59.72	71.07
WHO5 total	26.54	68.63	65.28	74.21
SWE total	31.48	71.24	65.28	73.58
PHQ4 total	30.25	69.93	68.06	74.84
PHQ4 anxiety	28.40	69.93	67.36	74.21
PHQ4 depression	29.01	69.28	65.28	73.58
ARI total	32.10	69.93	66.67	75.47
ARI total caretaker	14.20	35.29	57.64	66.67
CIS total	41.98	73.20	73.61	77.36
CIS total caretaker	38.89	56.21	65.97	71.70
EssenCES ZP	35.80	73.20	70.83	74.84
EssenCES SE	40.12	74.51	68.75	75.47
EssenCES TH	41.98	72.55	69.44	76.10

Table 5: Overview of percentage of missing data per questionnaire and time point. The table provides an overview of the cases in which the total score could not be calculated for ease of interpretation.

4.1.1 Specific methods

According to the work of Eekhout et al., it increases statistical power to impute the data on an item-level, instead of questionnaire level (Eekhout et al., 2014). We followed the method of parcel summaries to impute missing questionnaire data (Eekhout et al., 2018). This is a method to impute data of questionnaires that were collected multiple times, meaning that there are several observations for the same participant for the same questionnaire. Using these methods, the following data were included in the imputation models:

- Baseline covariates: age, sex, MAYSI-2 alcohol and drugs, MAYSI-2 anger irritability
- Group variables: randomization group, group within institution
- Drop out (yes/no)
- Other questionnaire items at the same time point
- Same item at other time points
- Mean score across all items at other time points. All questionnaires use Likert scale answer options, with a total score being the sum of all item scores. For this parcel method, we calculated the mean answer score across all items of the questionnaire at the particular time point that were filled in. Missing items, or items for which the participant responded that he/she did not want to disclose the answer, were not taken into account in the calculation of the mean score per item.

With this method, we use the important information from the other visits of the same questionnaire, but avoid the potential issue of incomplete or missing questionnaires at other time points, resulting in a missing total score. Furthermore, the mean scores allow us to include all questionnaire items without increasing the number of variables in the regression model used for the imputations to a very large number, making it unfeasible to perform reasonable imputations in the data set of 164 participants. In this case, it would not be possible to include every single item at each of the other three time points, which could result in 30 extra variables on a Likert scale (that are dummy coded) as predictors in the imputation model.

For the primary outcome AFQ, we imputed all missing items both at T1 and T3. For the secondary outcomes that were imputed on a subscale level if applicable, we followed the strategy of a previous START NOW study, that only imputed the items in case of limited number of missing items across the questionnaire according to the following schedule:

- Scale with < 4 items: no imputation
- Scale with 4-9 items: imputation if maximum 1 item was missing
- Scale with 10-15 items: imputation if maximum 2 items were missing
- Scale with 16-25 items: imputation if maximum 3 items were missing

In general, there were not a lot of single items missing, but rather complete questionnaires or complete visits that were missing. In the short PHQ4 questionnaire wit 4 items, this meant that the number of valid cases was reduced to 40, because we did not perform any imputations.

Final Report

Also for the other questionnaires we discarded many participants from the analysis due to a too high number of missing items in the T3 questionnaires especially.

A total of 50 imputed data sets were generated, and the results were combined follwing Rubin's rule (Rubin, 1987).

4.1.2 Answer option "do not want to disclose"

Not only missing items, but also the answer option "do not want to disclose" results in an impossibility to calculate the total questionnaire score (or subscale score) of a questionnaire. This was an answer option for most of the items of the secondary endpoints. An overview of the number of participants answering at least one question of the questionnaire with "do not want to disclose" is presented per time point in Table 6.

Variable	t1	t2	t3	t4
AFQ	0	0	0	0
CD RISC	17	7	6	3
CD RISC caretaker	8	7	5	11
WHO5	5	3	4	2
SWE	11	6	3	1
CIS	28	10	15	7
CIS caretaker	49	36	15	12
PHQ4	9	4	7	3
ARI	10	3	4	3
ARI caretaker	9	4	3	4
CSQ	0	20	0	0
EssenCES	33	13	17	6

Table 6: Overview of the number of participants with at least one question answered with 'do not wish to disclose' by questionnaire and time point.

Some participants just did not want to disclose some information, whereas some others completed entire questionnaires with this answer option. In a first stage, we treated this answer option as missing, because the result is the same, without a valid answer option, we cannot calculate the questionnaire score. Since this answer option is in most cases directly related to the variable value (i.e., the answer to the respective question), the data are missing not at random, because of which the main assumption for multiple imputations is not fulfilled.

4.2 Primary and secondary analyses

The analyses were conducted as described in the analysis plan, however, it was not possible to include the caregiver's previous experience with START NOW as a covariate, since only a few participants in the group-based training group had a caregiver with experience with the programme.[2]

4.3 Caretaker analysis

The caretaker analyses defined in the analysis plan were integrated in the secondary endpoints, as these are all questionnaires at a participant-level that were filled in by the participant's caretaker.[3]

Final Report

Additional analyses evaluating the influence of some caretaker characteristics on the primary analysis could unfortunately not be executed. The effect of the caregiver's previous experience with START NOW could not be assessed, as only caregivers in the group-based therapy had experience with START NOW. Working experience was not collected in a consistent way in the database (it was a free text field and the different caretakers did not provide the information in a uniform way) and was therefore not evaluable. Finally, the characteristics of the working circumstances were not collected. This information is indirectly included in the random effect for study centre that was included in all analyses.[4]

4.4 Supplementary analysis

The supplementary analyses described interaction tests between treatment group and some baseline participant characteristics. The multicategorical variables here were dichotomized as follows:[5]

- Psychological disease: yes (now) vs. no
- Duration of institutionalization: less than one year vs. one year or longer

Final Report

5 Results

5.1 Descriptive statistics

The baseline demographic characteristics of all participants are described by group in Tables 7 and 8. An overview of of the questionnaire results at screening (i.e., AFQ and MAYSI-2) is presented in Table 9. Finally, the caretaker characteristics are described in Table 10.

A between-group difference is seen for living in the institution, which is driven by the TAU group, in which all participants are living in the institution. We also see a much higher number of participants with a criminal offence in the TAU-group compared to the other three groups. These differences are most likely driven by one or a few specific centres being randomized to the TAU group and are the result of cluster randomization. We also see a difference in school between the groups, that can most likely be explained by centres as well.

No between-group differences are found for the screening questionnaires.

The caretakers in the different groups differ in terms of gender, profession, experience with START NOW, and motivation to participate. Given that these data were collected on a participant-level, the results are likely biased and driven by the number of participants one caretaker is responsible for.

Final Report

Final Report

5.1.1 Participant demographics

Table 7: Overview

	group (n=58)	self (n=58)	tau (n=46)	p
Age (y) (mean (SD))	16.6 (1.4)	16.3 (1.2)	16.9 (1.6)	0.090
Sex (%)				0.584
Male	32 (55.2)	34 (58.6)	24 (52.2)	
Female	26 (44.8)	24 (41.4)	21 (45.7)	
Diverse	0 (0.0)	0 (0.0)	1 (2.2)	
Country of birth (%)				0.651
Schweiz	36 (78.3)	35 (79.5)	30 (75.0)	
andere	10 (21.7)	8 (18.2)	10 (25.0)	
unbekannt	0 (0.0)	1 (2.3)	0 (0.0)	
Mother tongue = andere (%)	31 (67.4)	32 (72.7)	26 (65.0)	0.734
Living in the institution (%)				0.048
ja	37 (80.4)	37 (84.1)	40 (100.0)	
nein	8 (17.4)	7 (15.9)	0 (0.0)	
andere	1 (2.2)	0 (0.0)	0 (0.0)	
Type of living group (%)				NaN
offen	34 (91.9)	25 (67.6)	31 (77.5)	
halb-offen	1 (2.7)	8 (21.6)	7 (17.5)	
geschlossen	0 (0.0)	0 (0.0)	0 (0.0)	
andere	0 (0.0)	0 (0.0)	0 (0.0)	
unbekannt	2 (5.4)	4 (10.8)	2 (5.0)	
Tim in this institution (%)				0.376
6 Monate oder kürzer	17 (37.0)	16 (36.4)	8 (20.0)	
6 Monate bis 1 Jahr	10 (21.7)	8 (18.2)	14 (35.0)	
1 bis 2 Jahre	11 (23.9)	8 (18.2)	9 (22.5)	
mehr als 2 Jahre	8 (17.4)	12 (27.3)	9 (22.5)	
Previous institution (%)				0.138
nein	16 (34.8)	14 (31.8)	17 (42.5)	
in 1 anderen Institution/Pflegefamilie	12 (26.1)	16 (36.4)	5 (12.5)	
in 2 anderen Institutionen/Pflegefamilien	9 (19.6)	8 (18.2)	5 (12.5)	
in 3 oder mehr als 3 anderen Institutionen/Pflegefamilien	9 (19.6)	6 (13.6)	13 (32.5)	
Criminal offence = nein (%)	42 (91.3)	36 (81.8)	25 (62.5)	0.004
Release date within 6 months = nein (%)	36 (78.3)	33 (75.0)	29 (72.5)	0.824

Final Report

Table 8: Overview

	**group (n=58)**	**self (n=58)**	**tau (n=46)**	**p**
Previous living situation (%)				0.625
allein	0 (0.0)	0 (0.0)	1 (2.5)	
Herkunftsfamilie	29 (63.0)	25 (56.8)	24 (60.0)	
Verwandte/Bekannte	5 (10.9)	3 (6.8)	5 (12.5)	
Pflegefamilie	1 (2.2)	0 (0.0)	1 (2.5)	
Institution	9 (19.6)	13 (29.5)	9 (22.5)	
ohne festen Wohnsitz	1 (2.2)	0 (0.0)	0 (0.0)	
Untersuchungshaft	0 (0.0)	2 (4.5)	0 (0.0)	
andere	1 (2.2)	1 (2.3)	0 (0.0)	
School (%)				0.033
Schule in Institution	21 (45.7)	26 (59.1)	9 (22.5)	
Schule ausserhalb von Institution	6 (13.0)	8 (18.2)	13 (32.5)	
Ich gehe zurzeit nicht in die Schule	6 (13.0)	3 (6.8)	4 (10.0)	
Schule abgeschlossen. Ich befinde mich in einer Berufslehre.	9 (19.6)	3 (6.8)	11 (27.5)	
Ich besuche die Fachhochschule/Universität	0 (0.0)	0 (0.0)	1 (2.5)	
Ich bin arbeitslos.	4 (8.7)	4 (9.1)	2 (5.0)	
Education level (%)				NaN
(noch), keinen Abschluss	23 (50.0)	19 (43.2)	12 (30.0)	
Obligatorische Schulzeit abgeschlossen (Sekundar I, Volksschule)	17 (37.0)	21 (47.7)	23 (57.5)	
Berufslehre mit Eidgen. Fach-Zertifikat (EFZ)	1 (2.2)	1 (2.3)	0 (0.0)	
Berufslehre mit Berufsmatur (BM)	0 (0.0)	0 (0.0)	0 (0.0)	
Diplommittelschule (z.B. WMS, FMS, IMS)	0 (0.0)	0 (0.0)	1 (2.5)	
Matura/Gymnasium (Sekundar II)	3 (6.5)	0 (0.0)	2 (5.0)	
Fachhochschule / Universitätsabschluss	0 (0.0)	0 (0.0)	0 (0.0)	
Andere	2 (4.3)	3 (6.8)	2 (5.0)	
Chronic disease (%)				0.590
ja	12 (26.1)	11 (25.0)	11 (27.5)	
nein	31 (67.4)	30 (68.2)	29 (72.5)	
unbekannt	3 (6.5)	3 (6.8)	0 (0.0)	
Psychiatric disease (%)				0.861
ja	23 (50.0)	20 (45.5)	18 (45.0)	
nein	19 (41.3)	17 (38.6)	14 (35.0)	
früher	3 (6.5)	6 (13.6)	7 (17.5)	
unbekannt	1 (2.2)	1 (2.3)	1 (2.5)	

Table 9: Overview

	group (n=58)	self (n=58)	tau (n=46)	p
AFQ total (mean (SD))	36.8 (12.1)	31.8 (11.3)	35.0 (12.6)	0.078
MAYSI-2 alcohol-drugs (median [IQR])	2.0 [0.0, 4.0]	1.0 [0.0, 3.0]	2.0 [0.0, 5.0]	0.403
MAYSI-2 angry-irritable (median [IQR])	6.0 [5.0, 7.8]	5.5 [3.0, 7.0]	6.5 [5.0, 8.0]	0.149
MAYSI-2 depresses-anxious (median [IQR])	5.0 [3.0, 7.0]	5.0 [2.0, 6.0]	4.5 [2.0, 7.0]	0.572
MAYSI-2 somatic complaints (median [IQR])	2.5 [1.2, 4.0]	2.0 [1.0, 3.0]	3.0 [1.0, 4.0]	0.362
MAYSI-2 suicide idiation (median [IQR])	3.0 [1.0, 5.0]	1.0 [0.0, 4.0]	3.0 [0.0, 5.0]	0.096
MAYSI-2 thought disturbance (boys) (median [IQR])	1.0 [0.0, 3.0]	1.0 [0.2, 3.0]	1.0 [0.8, 2.0]	0.943

Final Report

5.1.2 Caretakers

Table 10: Overview of the caretaker characteristics

	group (n=25)	self (n=32)	tau (n=24)	p
Sex (%)				0.258
divers	0 (0.0)	0 (0.0)	2 (8.3)	
männlich	9 (36.0)	13 (40.6)	10 (41.7)	
weiblich	16 (64.0)	19 (59.4)	12 (50.0)	
Profession (%)				0.660
Anderer Beruf	1 (4.0)	1 (3.1)	1 (4.2)	
ErzieherIn	8 (32.0)	7 (21.9)	8 (33.3)	
PsychologIn	3 (12.0)	4 (12.5)	0 (0.0)	
SozialpädagogIn/SozialarbeiterIn	13 (52.0)	20 (62.5)	15 (62.5)	
START NOW training = M (%)	22 (88.0)	29 (90.6)	21 (87.5)	0.921
Previous experience with START NOW = nein (%)	19 (76.0)	32 (100.0)	24 (100.0)	0.001
Self motivated to participate = nein (%)	3 (12.0)	8 (25.0)	3 (12.5)	0.332
Trauma or resiliance training in last year = nein (%)	18 (72.0)	23 (71.9)	21 (87.5)	0.320
Experience with supervision = nein (%)	10 (40.0)	8 (25.0)	6 (25.0)	0.394

Final Report

5.1.3 Questionnaire data over time

Table 11 and Figures 1 and 2 present the time course of the mean questionnaire or subscale score for each of the three groups over the different study visits for the participants included in the FAS. Be aware that the scale of the Y-axis of each of the sub-figures is related to the questionnaire score, and differs between the different scores and subscales.

It must be noticed that the number of participants decreases over time and then increases at T4, which was explained by the incentive the participants received for study participation when the T4 questionnaires were filled in. The numbers represent the total number of participants with available data at the study visit and not the number of participants with a complete questionnaire. Unfortunately, the last number is significantly lower. As is seen in the figures, only few questionnaires show a clear pattern over time. The time course for the different groups did differ for some questionnaires, but looking at the scores on the Y-axis, no large differences were seen.

Table 11: Overview of the different questionnaire scores across time points.

	**t1 (n=162)**	**t2 (n=153)**	**t3 (n=144)**	**t4 (n=159)**
AFQ total (mean (SD))	34.5 (12.3)	34.2 (11.5)	33.6 (12.1)	33.1 (11.7)
CD-RISC total (mean (SD))	21.9 (7.7)	23.2 (6.8)	20.5 (6.1)	21.8 (7.6)
CD-RISC total caretaker (mean (SD))	25.4 (9.2)	23.9 (8.3)	26.8 (7.6)	26.3 (9.2)
WHO5 total (mean (SD))	12.8 (6.1)	11.1 (5.9)	11.1 (5.8)	11.3 (6.0)
SWE total (mean (SD))	27.5 (6.0)	28.3 (5.7)	27.1 (6.1)	27.2 (5.6)
PHQ4 total (mean (SD))	4.9 (3.1)	5.2 (3.0)	5.8 (3.4)	5.6 (2.6)
PHQ4 anxiety (mean (SD))	2.4 (1.9)	2.7 (1.8)	2.9 (1.9)	2.7 (1.5)
PHQ4 depression (mean (SD))	2.4 (1.6)	2.6 (1.6)	2.9 (1.7)	2.8 (1.4)
ARI total (mean (SD))	3.9 (3.2)	5.0 (3.3)	3.9 (3.1)	3.7 (2.6)
ARI total caretaker (mean (SD))	3.7 (3.0)	3.5 (3.2)	3.5 (3.0)	2.7 (1.8)
CIS total (mean (SD))	17.8 (9.3)	20.0 (8.7)	20.8 (10.8)	18.1 (9.4)
CIS total caretaker (mean (SD))	24.1 (9.2)	24.6 (8.6)	22.8 (7.2)	19.0 (7.9)
EssenCES ZP (mean (SD))	11.0 (5.1)	13.1 (3.6)	10.5 (4.2)	10.6 (5.2)
EssenCES SE (mean (SD))	14.1 (4.4)	13.9 (4.4)	14.2 (4.2)	14.1 (4.5)
EssenCES TH (mean (SD))	13.2 (4.4)	13.3 (4.4)	13.2 (3.9)	13.1 (4.5)

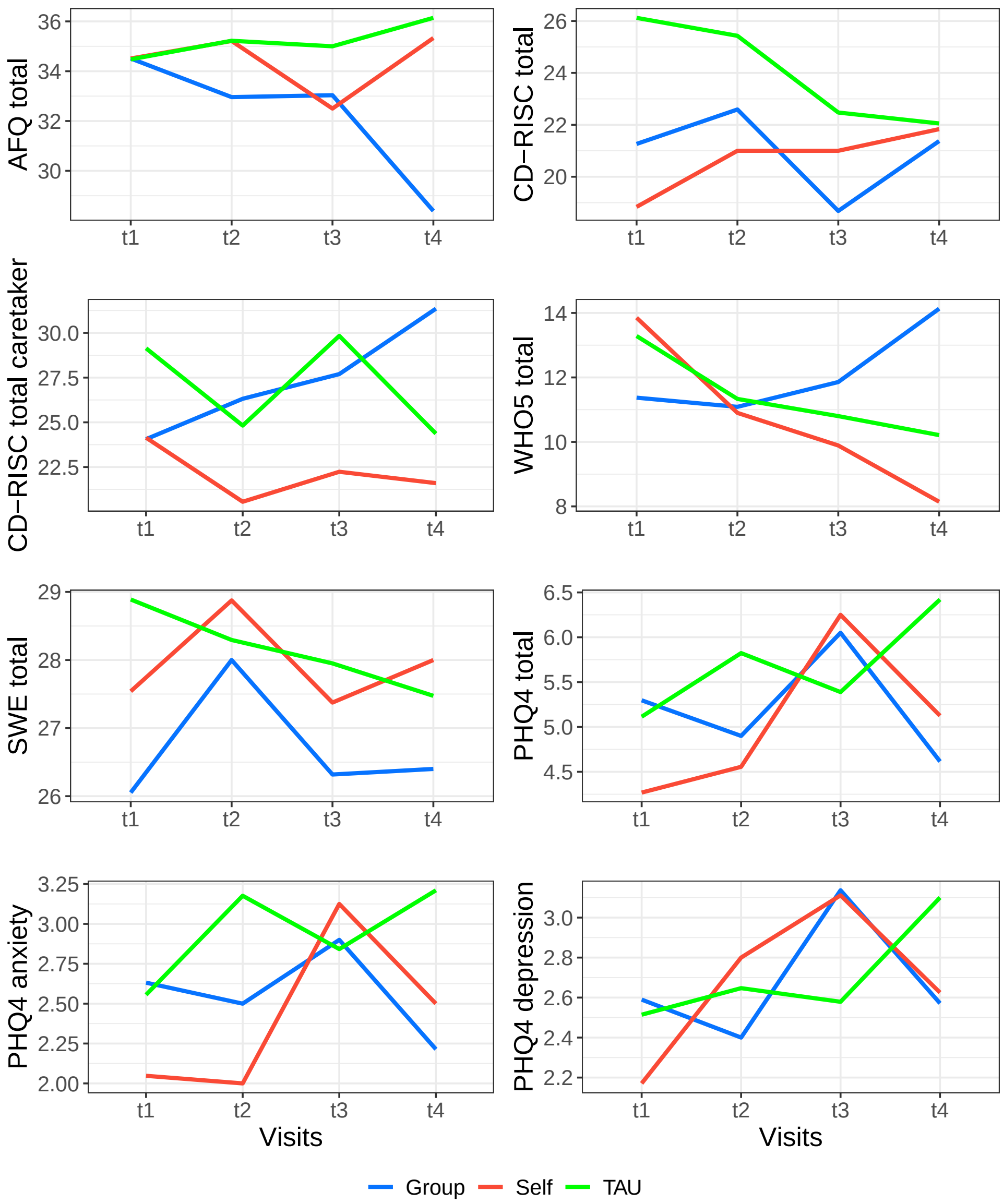

Figure 1: Timecourse of the different questionnaire scores (subplots) per group (colors). Part 1.

Final Report

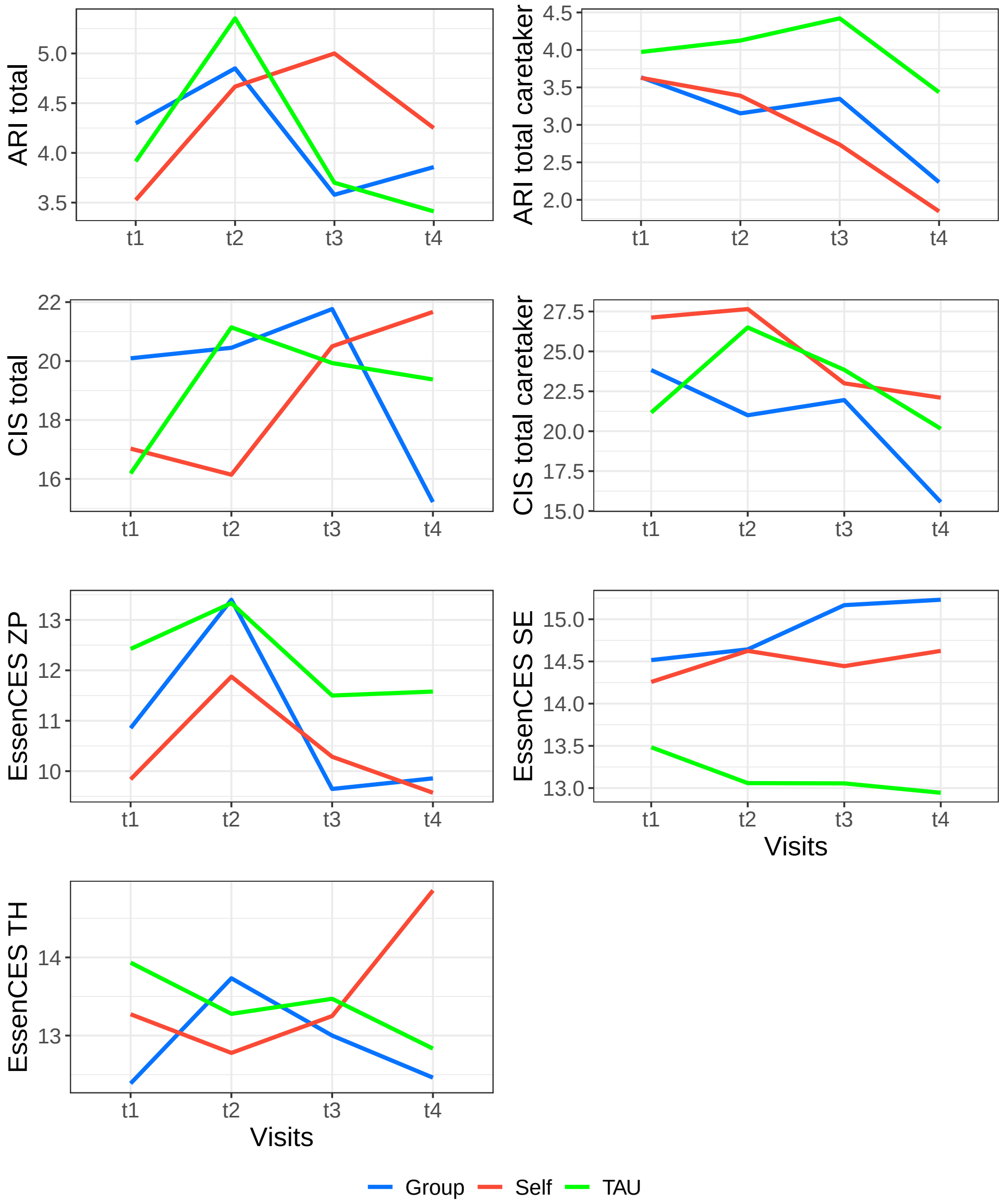

Figure 2: Timecourse of the different questionnaire scores (subplots) per group (colors). Part 2.

Final Report

5.2 Primary analysis

The primary analysis aimed to assess the effect of the START NOW training programmes on the AFQ total score at T3. In the first analysis, we set TAU as a reference level to compare both treatments to the "control" condition. The results are presented in Table 12. The table includes the estimates with 95% confidence interval (CI) and p-value for the treatment groups as well as all covariates included in the model. We tested a possible interaction between treatment and both MAYSI-II subscales, but both did not have a significant effect (p-values > 0.7) and were therefore left out. The table shows that there is no difference in AFQ score at T3 between TAU and any of the two treatment groups. The strongest association was found for the AFQ score at T1.

	Estimate	95% CI	p-vaue
Treatment (group over TAU)	-1.222	[-7.057, 4.613]	0.678
Treatment (self over TAU)	-1.124	[-7.083, 4.834]	0.708
AFQ total at T1	0.296	[0.09, 0.503]	0.005
Age (years)	0.359	[-1.281, 2]	0.664
Gender (female over male)	1.528	[-3.48, 6.536]	0.545
Gender (diverse over male)	1.243	[-32.176, 34.662]	0.941
MAYSI II AI	-1.073	[-2.293, 0.147]	0.084
MAYSI II DA	-0.025	[-1.246, 1.196]	0.968

Table 12: Results of the primary analysis. The results represent the pooled estimates with corresponding 95% confidence interval and p-value of the linear mixed effects model. The analysis was performed in the imputed FAS.

When we change the reference group to self-guided therapy (Table 13), we see that also here there is no difference between the group-based and self-guided therapy. The results including the other covariates.

	Estimate	95% CI	p-vaue
Treatment (group over self)	-0.097	[-5.558, 5.363]	0.972
Treatment (TAU over self)	1.124	[-4.834, 7.083]	0.708
AFQ total at T1	0.296	[0.09, 0.503]	0.005
Age (years)	0.359	[-1.281, 2]	0.664
Gender (female over male)	1.528	[-3.48, 6.536]	0.545
Gender (diverse over male)	1.243	[-32.176, 34.662]	0.941
MAYSI II AI	-1.073	[-2.293, 0.147]	0.084
MAYSI II DA	-0.025	[-1.246, 1.196]	0.968

Table 13: Results of the primary analysis with self-helped training as reference level. The results represent the pooled estimates with corresponding 95% confidence interval and p-value of the linear mixed effects model. The analysis was performed in the imputed FAS.

Final Report

5.3 Secondary analyses

5.3.1 Secondary endpoints

Table 14 shows the estimates with corresponding confidence interval and p-value for the comparisons between treatment groups for each of the secondary endpoints. All analyses were performed in the imputed FAS, in which only imputations were performed in accordance with the methods described above. The consequence of this imputation strategy, is that the number of participants included in the respective analyses lies between 40 and 164. This means that the sample was highly reduced for the secondary analyses, and that results should be interpreted with more caution.

The results show that there are no signs of difference between groups for any of the secondary endpoints. The detailed results tables, including the estimates for all covariates, are found under Supplementary tables (Tables 26 to 53).

Final Report

Final Report

Outcome	Group	Estimate	95% CI	p-value
CD-RISC total	group over TAU	-1.625	[-4.482, 1.233]	0.261
	self over TAU	-0.691	[-3.915, 2.533]	0.670
	group over self	-0.933	[-3.819, 1.953]	0.520
CD-RISC total caretaker	group over TAU	-1.516	[-7.622, 4.59]	0.621
	self over TAU	-7.586	[-13.766, -1.406]	0.017
	group over self	6.070	[0.077, 12.063]	0.047
WHO5 total	group over TAU	1.144	[-1.98, 4.268]	0.465
	self over TAU	-2.546	[-6.608, 1.516]	0.213
	group over self	3.690	[-0.108, 7.488]	0.057
SWE total	group over TAU	-0.625	[-2.725, 1.475]	0.557
	self over TAU	-0.865	[-3.018, 1.287]	0.427
	group over self	0.241	[-1.737, 2.218]	0.810
PHQ4 total	group over TAU	0.659	[-2.05, 3.367]	0.622
	self over TAU	-0.871	[-3.714, 1.972]	0.535
	group over self	1.529	[-1.04, 4.098]	0.233
PHQ4 anxiety	group over TAU	0.683	[-0.825, 2.192]	0.362
	self over TAU	-0.214	[-1.819, 1.392]	0.788
	group over self	0.897	[-0.561, 2.355]	0.218
PHQ4 depression	group over TAU	0.308	[-1.016, 1.632]	0.638
	self over TAU	-0.319	[-1.716, 1.079]	0.645
	group over self	0.627	[-0.631, 1.885]	0.317
ARI total	group over TAU	-0.205	[-2.341, 1.93]	0.847
	self over TAU	1.436	[-1.199, 4.072]	0.278
	group over self	-1.642	[-4.14, 0.856]	0.192
ARI total caretaker	group over TAU	-0.780	[-2.474, 0.914]	0.361
	self over TAU	-0.923	[-2.613, 0.767]	0.279
	group over self	0.143	[-1.442, 1.728]	0.858
CIS total	group over TAU	0.187	[-5.281, 5.655]	0.945
	self over TAU	4.839	[-1.965, 11.643]	0.159
	group over self	-4.652	[-11.056, 1.753]	0.150
CIS total caretaker	group over TAU	-1.906	[-6.262, 2.45]	0.385
	self over TAU	-1.498	[-5.827, 2.832]	0.492
	group over self	-0.409	[-4.584, 3.766]	0.845
EssenCES ZP	group over TAU	-0.422	[-2.76, 1.917]	0.718
	self over TAU	1.181	[-1.798, 4.161]	0.427
	group over self	-1.603	[-4.409, 1.204]	0.254
EssenCES SE	group over TAU	0.413	[-2.16, 2.986]	0.747
	self over TAU	-1.292	[-4.514, 1.931]	0.422
	group over self	1.705	[-1.451, 4.861]	0.280
EssenCES TH	group over TAU	1.227	[-0.951, 3.405]	0.262
	self over TAU	-0.178	[-2.994, 2.639]	0.899
	group over self	1.405	[-1.436, 4.246]	0.321

Table 14: Overview table of the results for treatment group for all secondary outcomes. The results represent the pooled, adjusted estimates with corresponding 95% confidence interval and p-value of the linear mixed effects model. The analysis was performed in the imputed FAS.

5.3.2 Long term effect AFQ

An additional secondary analysis was the assessment of the long-term effect of the interventions on the AFQ-score. The results of this analysis with TAU as a reference level are displayed in Table 15. No difference between the treatment groups was found.

	Estimate	95% CI	p-vaue
Treatment (group over TAU)	1.227	[-0.951, 3.405]	0.262
Treatment (self over TAU)	-0.178	[-2.994, 2.639]	0.899
EssenCES TH at T1	0.455	[0.237, 0.673]	0.000
Age (years)	0.192	[-0.478, 0.862]	0.566
Gender (female over male)	0.694	[-1.333, 2.721]	0.493
MAYSI II AI	0.515	[-0.073, 1.102]	0.084
MAYSI II DA	-0.223	[-0.709, 0.262]	0.358

Table 15: Results of the analysis for the long term effect on AFQ (T4). The results represent the pooled estimates with corresponding 95% confidence interval and p-value of the linear mixed effects model. The analysis was performed in the imputed FAS.

Final Report

5.4 Secondary estimand: per protocol analysis

Table 16 shows the estimates with corresponding confidence interval and p-value for the comparisons between treatment groups for each of the endpoints assessed in the per protocol population. Also here, it must be noted that due to the maximum number of missing questionnaire items allowed for imputation of the secondary endpoints, the number of observations in each of the models is highly reduced.

The results show that there are no signs of difference between groups for any of the endpoints. Due to the reduced number of participants used for this analysis, the statistical power is lower compared to the analysis in the FAS.

The detailed results tables, including the estimates for all covariates, are found under Supplementary tables (Tables 54 to 83).

Final Report

Final Report

Outcome	Group	Estimate	95% CI	p-value
AFQ total	group over TAU	-0.378	[-8.701, 7.946]	0.928
	self over TAU	1.557	[-4.927, 8.042]	0.631
	group over self	0.378	[-7.946, 8.701]	0.928
CD-RISC total	group over TAU	1.399	[-2.568, 5.365]	0.482
	self over TAU	1.661	[-1.606, 4.928]	0.312
	group over self	-1.399	[-5.365, 2.568]	0.482
CD-RISC total caretaker	group over TAU	-5.060	[-13.077, 2.957]	0.209
	self over TAU	-0.099	[-6.764, 6.567]	0.976
	group over self	5.060	[-2.957, 13.077]	0.209
WHO5 total	group over TAU	-3.648	[-8.433, 1.137]	0.131
	self over TAU	-1.613	[-5.444, 2.218]	0.399
	group over self	3.648	[-1.137, 8.433]	0.131
SWE total	group over TAU	-0.046	[-3.255, 3.163]	0.977
	self over TAU	0.154	[-2.245, 2.553]	0.899
	group over self	0.046	[-3.163, 3.255]	0.977
PHQ4 total	group over TAU	0.981	[-3.441, 5.403]	0.640
	self over TAU	-0.995	[-4.011, 2.02]	0.489
	group over self	-0.981	[-5.403, 3.441]	0.640
PHQ4 anxiety	group over TAU	0.816	[-1.765, 3.397]	0.511
	self over TAU	-0.825	[-2.435, 0.785]	0.292
	group over self	-0.816	[-3.397, 1.765]	0.511
PHQ4 depression	group over TAU	0.421	[-2.155, 2.997]	0.732
	self over TAU	-0.453	[-2.055, 1.15]	0.555
	group over self	-0.421	[-2.997, 2.155]	0.732
ARI total	group over TAU	1.191	[-1.771, 4.153]	0.420
	self over TAU	0.411	[-1.946, 2.769]	0.726
	group over self	-1.191	[-4.153, 1.771]	0.420
ARI total caretaker	group over TAU	0.050	[-2.599, 2.7]	0.970
	self over TAU	0.499	[-1.486, 2.484]	0.615
	group over self	-0.050	[-2.7, 2.599]	0.970
CIS total	group over TAU	2.486	[-5.157, 10.129]	0.514
	self over TAU	0.426	[-5.513, 6.364]	0.885
	group over self	-2.486	[-10.129, 5.157]	0.514
CIS total caretaker	group over TAU	-0.598	[-7.172, 5.976]	0.855
	self over TAU	1.198	[-3.913, 6.308]	0.639
	group over self	0.598	[-5.976, 7.172]	0.855
EssenCES ZP	group over TAU	1.020	[-2.137, 4.177]	0.515
	self over TAU	0.389	[-2.154, 2.932]	0.757
	group over self	-1.020	[-4.177, 2.137]	0.515
EssenCES SE	group over TAU	0.395	[-2.837, 3.628]	0.805
	self over TAU	-0.474	[-3.198, 2.25]	0.724
	group over self	-0.395	[-3.628, 2.837]	0.805
EssenCES TH	group over TAU	-1.259	[-4.349, 1.831]	0.413
	self over TAU	-1.216	[-3.506, 1.073]	0.287
	group over self	1.259	[-1.831, 4.349]	0.413

Table 16: Overview table of the results for treatment group for all secondary outcomes. The results represent the pooled, adjusted estimates with corresponding 95% confidence interval and p-value of the linear mixed effects model. The analysis was performed in the imputed PP analysis set.

5.5 Supplementary analysis

For this analysis, all categorical variables were dichotomized. For psychological disease we made this a yes/no variable, and duration of institutionalization was categorized as less than one year, compared to more than one year. As a supplementary analyses, we wanted to investigate the influence of certain patient characteristics on the treatment effect in the different groups in the FAS. These analyses were performed for the comparison between TAU and each of the training groups. Table 17 shows the results of the interaction effects between treatment and the participant characteristic of interest (listed in the column "Variable in interaction").

For none of the variables we found a significant interaction effect, meaning that it is not likely that these patient characteristics influence the effect of the treatment groups on the AFQ score at T3.

Detailed result tables can be found under Supplementary tables (Tables 84 to 87).

Variable in interaction	Group	Estimate	95% CI	p-value
duration of institutionalization	Interaction with group over TAU	-1.343	[-13.781, 11.096]	0.830
	Interaction with self over TAU			
age	Interaction with group over TAU	1.682	[-12.3, 15.665]	0.810
	Interaction with self over TAU			
diagnosis of psychological disease	Interaction with group over TAU			
	Interaction with self over TAU			
previous stay in institution or care family	Interaction with group over TAU	0.424	[-12.293, 13.141]	0.947
	Interaction with self over TAU			

Table 17: Overview table of the results for treatment group for all secondary outcomes. The results represent the pooled, adjusted estimates with corresponding 95% confidence interval and p-value of the linear mixed effects model. The analysis was performed in the imputed PP analysis set.

Final Report

Final Report

5.6 Safety analysis

The (S)AEs that occurred during the study are described in Table 18. It is not likely that any of the AEs that occurred are related to the intervention. Please note that we did not add the description of the AEs to save space.

Table 18: Overview

	**group (n=6)**	**self (n=9)**	**tau (n=15)**
Type = Serious Adverse Event (%)	0 (0.0)	3 (37.5)	6 (40.0)
Evaluation = moderat (%)	1 (16.7)	2 (40.0)	4 (44.4)
Causality (%)			
Definitiv	0 (0.0)	0 (0.0)	0 (0.0)
Wahrscheinlich	0 (0.0)	0 (0.0)	0 (0.0)
Möglich	0 (0.0)	0 (0.0)	0 (0.0)
Unwahrscheinlich	0 (0.0)	1 (11.1)	0 (0.0)
Kein Zusammenhang	6 (100.0)	8 (88.9)	15 (100.0)
Nicht abschätzbar	0 (0.0)	0 (0.0)	0 (0.0)
Action (%)			
Keine	5 (83.3)	1 (11.1)	1 (6.7)
Hospitalisierung	0 (0.0)	2 (22.2)	4 (26.7)
Medikamente	0 (0.0)	0 (0.0)	0 (0.0)
Gespräche	0 (0.0)	3 (33.3)	8 (53.3)
Sonstiges	1 (16.7)	3 (33.3)	2 (13.3)
Result (%)			
Erholt/Problem gelöst	4 (66.7)	3 (33.3)	2 (13.3)
In Erholung	1 (16.7)	1 (11.1)	2 (13.3)
Nicht erholt/gelöst	1 (16.7)	4 (44.4)	11 (73.3)
Tödlich	0 (0.0)	0 (0.0)	0 (0.0)
Unbekannt	0 (0.0)	0 (0.0)	0 (0.0)
Andauernd (am Ende der Studie)	0 (0.0)	1 (11.1)	0 (0.0)

Final Report

5.7 Sensitivity analysis

5.7.1 Complete cases

As a sensitivity analysis, we repeated the primary analysis in the complete cases data set, which means we had to discard the participants with missing data for any of the variables in the primary analysis (either outcome, AFQ score at T1, or any of the baseline covariates). In total, only 59 participants were included in this analysis, due to missing data for mainly the AFQ at T3. None of the baseline covariates showed missing data. The results of the sensitivity analysis show no difference to the primary analysis. We do not see a difference between the training groups, and only AFQ at T1 is an important predictor of the questionnaire score at T3.

	estimate	95% CI	p-value
Treatment (group over TAU)	-1.172	[-6.730, 4.254]	0.698
Treatment (self over TAU)	-2.783	[-9.541, 4.078]	0.468
AFQ total at T1	0.593	[0.362, 0.814]	<0.001
Age (years)	-0.022	[-1.555, 1.593]	0.979
Gender (female over male)	2.081	[-3.080, 7.326]	0.465
MAYSI II AI	-0.707	[-2.156, 0.755]	0.372
MAYSI II DA	0.653	[-0.637, 1.867]	0.337

Table 19: Results of the sensitivity analysis for the effect of the training on AFQ (T3) considering only the complete cases. The results represent the estimates with corresponding 95% confidence interval and p-value of the linear mixed effects model.

5.7.2 Dropout

In the tables below (Tables 20 to 22) we compare the baseline characteristics of the participants who dropped out compared to the other patients. No differences were seen for any of the demographic or questionnaire variables except for criminal offence and previous living situation.

Final Report

Table 20: Overview

	0 (n=150)	1 (n=12)	p
Age (y) (mean (SD))	16.6 (1.5)	16.4 (1.4)	0.662
Sex (%)			0.876
Male	84 (56.0)	6 (50.0)	
Female	65 (43.3)	6 (50.0)	
Diverse	1 (0.7)	0 (0.0)	
Country of birth (%)			0.976
Schweiz	97 (77.6)	4 (80.0)	
andere	27 (21.6)	1 (20.0)	
unbekannt	1 (0.8)	0 (0.0)	
Mother tongue = andere (%)	84 (67.2)	5 (100.0)	0.291
Living in the institution (%)			0.126
ja	111 (88.8)	3 (60.0)	
nein	13 (10.4)	2 (40.0)	
andere	1 (0.8)	0 (0.0)	
Type of living group (%)			NaN
offen	87 (78.4)	3 (100.0)	
halb-offen	16 (14.4)	0 (0.0)	
geschlossen	0 (0.0)	0 (0.0)	
andere	0 (0.0)	0 (0.0)	
unbekannt	8 (7.2)	0 (0.0)	
Tim in this institution (%)			0.473
6 Monate oder kürzer	38 (30.4)	3 (60.0)	
6 Monate bis 1 Jahr	31 (24.8)	1 (20.0)	
1 bis 2 Jahre	28 (22.4)	0 (0.0)	
mehr als 2 Jahre	28 (22.4)	1 (20.0)	
Previous institution (%)			0.557
nein	46 (36.8)	1 (20.0)	
in 1 anderen Institution/Pflegefamilie	32 (25.6)	1 (20.0)	
in 2 anderen Institutionen/Pflegefamilien	20 (16.0)	2 (40.0)	
in 3 oder mehr als 3 anderen Institutionen/Pflegefamilien	27 (21.6)	1 (20.0)	
Criminal offence = nein (%)	101 (80.8)	2 (40.0)	0.100
Release date within 6 months = nein (%)	94 (75.2)	4 (80.0)	1.000

Final Report

Table 21: Overview

	0 (n=150)	1 (n=12)	p
Previous living situation (%)			0.047
allein	1 (0.8)	0 (0.0)	
Herkunftsfamilie	77 (61.6)	1 (20.0)	
Verwandte/Bekannte	12 (9.6)	1 (20.0)	
Pflegefamilie	2 (1.6)	0 (0.0)	
Institution	29 (23.2)	2 (40.0)	
ohne festen Wohnsitz	1 (0.8)	0 (0.0)	
Untersuchungshaft	2 (1.6)	0 (0.0)	
andere	1 (0.8)	1 (20.0)	
School (%)			0.817
Schule in Institution	53 (42.4)	3 (60.0)	
Schule ausserhalb von Institution	26 (20.8)	1 (20.0)	
Ich gehe zurzeit nicht in die Schule	12 (9.6)	1 (20.0)	
Schule abgeschlossen. Ich befinde mich in einer Berufslehre.	23 (18.4)	0 (0.0)	
Ich besuche die Fachhochschule/Universität	1 (0.8)	0 (0.0)	
Ich bin arbeitslos.	10 (8.0)	0 (0.0)	
Education level (%)			NaN
(noch), keinen Abschluss	51 (40.8)	3 (60.0)	
Obligatorische Schulzeit abgeschlossen (Sekundar I, Volksschule)	59 (47.2)	2 (40.0)	
Berufslehre mit Eidgen. Fach-Zertifikat (EFZ)	2 (1.6)	0 (0.0)	
Berufslehre mit Berufsmatur (BM)	0 (0.0)	0 (0.0)	
Diplommittelschule (z.B. WMS, FMS, IMS)	1 (0.8)	0 (0.0)	
Matura/Gymnasium (Sekundar II)	5 (4.0)	0 (0.0)	
Fachhochschule / Universitätsabschluss	0 (0.0)	0 (0.0)	
Andere	7 (5.6)	0 (0.0)	
Chronic disease (%)			0.710
ja	32 (25.6)	2 (40.0)	
nein	87 (69.6)	3 (60.0)	
unbekannt	6 (4.8)	0 (0.0)	
Psychiatric disease (%)			0.813
ja	58 (46.4)	3 (60.0)	
nein	48 (38.4)	2 (40.0)	
früher	16 (12.8)	0 (0.0)	
unbekannt	3 (2.4)	0 (0.0)	

Table 22: Overview

	0 (n=150)	1 (n=12)	p
AFQ total (mean (SD))	34.6 (12.1)	33.2 (11.6)	0.696
MAYSI-2 alcohol-drugs (median [IQR])	1.0 [0.0, 4.0]	2.5 [1.8, 4.8]	0.152
MAYSI-2 angry-irritable (median [IQR])	6.0 [4.0, 8.0]	5.0 [2.8, 6.5]	0.379
MAYSI-2 depresses-anxious (median [IQR])	5.0 [2.0, 7.0]	4.0 [1.8, 5.5]	0.405
MAYSI-2 somatic complaints (median [IQR])	2.0 [1.0, 4.0]	2.5 [1.8, 3.0]	0.680
MAYSI-2 suicide idiation (median [IQR])	3.0 [0.0, 4.0]	0.5 [0.0, 4.2]	0.520
MAYSI-2 thought disturbance (boys) (median [IQR])	1.0 [0.0, 3.0]	1.0 [1.0, 1.8]	0.913

5.7.3 Compliers

In the tables below (Tables 23 to 25) we compare the baseline characteristics of the participants who were considered compliers compared to the other patients. No differences were seen for any of the demographic or questionnaire variables.

Table 23: Overview

	0 (n=55)	1 (n=109)	p
Age (y) (mean (SD))	16.4 (1.3)	16.7 (1.5)	0.276
Sex (%)			0.567
Male	32 (60.4)	58 (53.2)	
Female	21 (39.6)	50 (45.9)	
Diverse	0 (0.0)	1 (0.9)	
Country of birth (%)			0.227
Schweiz	25 (75.8)	76 (78.4)	
andere	7 (21.2)	21 (21.6)	
unbekannt	1 (3.0)	0 (0.0)	
Mother tongue = andere (%)	24 (72.7)	65 (67.0)	0.694
Living in the institution (%)			0.115
ja	26 (78.8)	88 (90.7)	
nein	7 (21.2)	8 (8.2)	
andere	0 (0.0)	1 (1.0)	
Type of living group (%)			NaN
offen	19 (73.1)	71 (80.7)	
halb-offen	6 (23.1)	10 (11.4)	
geschlossen	0 (0.0)	0 (0.0)	
andere	0 (0.0)	0 (0.0)	
unbekannt	1 (3.8)	7 (8.0)	
Tim in this institution (%)			0.631
6 Monate oder kürzer	10 (30.3)	31 (32.0)	
6 Monate bis 1 Jahr	7 (21.2)	25 (25.8)	
1 bis 2 Jahre	6 (18.2)	22 (22.7)	
mehr als 2 Jahre	10 (30.3)	19 (19.6)	
Previous institution (%)			0.091
nein	8 (24.2)	39 (40.2)	
in 1 anderen Institution/Pflegefamilie	11 (33.3)	22 (22.7)	
in 2 anderen Institutionen/Pflegefamilien	9 (27.3)	13 (13.4)	
in 3 oder mehr als 3 anderen Institutionen/Pflegefamilien	5 (15.2)	23 (23.7)	
Criminal offence = nein (%)	26 (78.8)	77 (79.4)	1.000
Release date within 6 months = nein (%)	25 (75.8)	73 (75.3)	1.000

Final Report

Table 24: Overview

	0 (n=55)	1 (n=109)	p
Previous living situation (%)			0.830
allein	0 (0.0)	1 (1.0)	
Herkunftsfamilie	20 (60.6)	58 (59.8)	
Verwandte/Bekannte	2 (6.1)	11 (11.3)	
Pflegefamilie	0 (0.0)	2 (2.1)	
Institution	9 (27.3)	22 (22.7)	
ohne festen Wohnsitz	0 (0.0)	1 (1.0)	
Untersuchungshaft	1 (3.0)	1 (1.0)	
andere	1 (3.0)	1 (1.0)	
School (%)			0.382
Schule in Institution	19 (57.6)	37 (38.1)	
Schule ausserhalb von Institution	6 (18.2)	21 (21.6)	
Ich gehe zurzeit nicht in die Schule	2 (6.1)	11 (11.3)	
Schule abgeschlossen. Ich befinde mich in einer Berufslehre.	3 (9.1)	20 (20.6)	
Ich besuche die Fachhochschule/Universität	0 (0.0)	1 (1.0)	
Ich bin arbeitslos.	3 (9.1)	7 (7.2)	
Education level (%)			NaN
(noch), keinen Abschluss	18 (54.5)	36 (37.1)	
Obligatorische Schulzeit abgeschlossen (Sekundar I, Volksschule)	13 (39.4)	48 (49.5)	
Berufslehre mit Eidgen. Fach-Zertifikat (EFZ)	1 (3.0)	1 (1.0)	
Berufslehre mit Berufsmatur (BM)	0 (0.0)	0 (0.0)	
Diplommittelschule (z.B. WMS, FMS, IMS)	0 (0.0)	1 (1.0)	
Matura/Gymnasium (Sekundar II)	0 (0.0)	5 (5.2)	
Fachhochschule / Universitätsabschluss	0 (0.0)	0 (0.0)	
Andere	1 (3.0)	6 (6.2)	
Chronic disease (%)			0.877
ja	9 (27.3)	25 (25.8)	
nein	23 (69.7)	67 (69.1)	
unbekannt	1 (3.0)	5 (5.2)	
Psychiatric disease (%)			0.918
ja	16 (48.5)	45 (46.4)	
nein	13 (39.4)	37 (38.1)	
früher	3 (9.1)	13 (13.4)	
unbekannt	1 (3.0)	2 (2.1)	

Final Report

Table 25: Overview

	0 (n=55)	1 (n=109)	p
AFQ total (mean (SD))	32.9 (12.2)	35.3 (11.9)	0.236
MAYSI-2 alcohol-drugs (median [IQR])	2.0 [0.0, 4.0]	2.0 [0.0, 4.0]	0.847
MAYSI-2 angry-irritable (median [IQR])	6.0 [3.0, 7.0]	6.0 [5.0, 8.0]	0.404
MAYSI-2 depresses-anxious (median [IQR])	5.0 [2.0, 6.0]	5.0 [2.8, 7.0]	0.217
MAYSI-2 somatic complaints (median [IQR])	2.0 [1.0, 3.0]	3.0 [1.0, 4.0]	0.275
MAYSI-2 suicide idiation (median [IQR])	1.0 [0.0, 4.0]	3.0 [0.0, 5.0]	0.134
MAYSI-2 thought disturbance (boys) (median [IQR])	1.0 [0.0, 3.0]	1.0 [0.0, 3.0]	0.727

6 Supplementary tables

6.1 Full tables of the secondary endpoints

The tables below present the results of the secondary analyses, including the estimates for all covariates in the models.

6.1.1 TAU as reference

	Estimate	95% CI	p-vaue
Treatment (group over TAU)	-1.625	[-4.482, 1.233]	0.261
Treatment (self over TAU)	-0.691	[-3.915, 2.533]	0.670
CD-RISC total at T1	0.038	[-0.133, 0.209]	0.658
Age (years)	0.280	[-0.501, 1.062]	0.475
Gender (female over male)	0.825	[-1.692, 3.342]	0.514
Gender (diverse over male)	-1.486	[-13.164, 10.192]	0.800
MAYSI II AI	0.248	[-0.346, 0.841]	0.406
MAYSI II DA	-0.141	[-0.767, 0.486]	0.654

Table 26: Results of the analysis for outcome CD-RISC total with TAU as reference group. The results represent the pooled estimates with corresponding 95% confidence interval and p-value of the linear mixed effects model. The analysis was performed in the imputed FAS (imputations in accordance with specified methods).

	Estimate	95% CI	p-vaue
Treatment (group over TAU)	1.144	[-1.98, 4.268]	0.465
Treatment (self over TAU)	-2.546	[-6.608, 1.516]	0.213
WHO5 total at T1	0.294	[0.056, 0.531]	0.017
Age (years)	-0.489	[-1.459, 0.481]	0.316
Gender (female over male)	-1.944	[-4.778, 0.891]	0.174
MAYSI II AI	-0.295	[-1.104, 0.514]	0.466
MAYSI II DA	-0.519	[-1.221, 0.183]	0.143

Table 27: Results of the analysis for outcome WHO5 total with TAU as reference group. The results represent the pooled estimates with corresponding 95% confidence interval and p-value of the linear mixed effects model. The analysis was performed in the imputed FAS (imputations in accordance with specified methods).

	Estimate	95% CI	p-vaue
Treatment (group over TAU)	0.187	[-5.281, 5.655]	0.945
Treatment (self over TAU)	4.839	[-1.965, 11.643]	0.159
CIS total at T1	0.278	[-0.009, 0.564]	0.057
Age (years)	1.226	[-0.39, 2.841]	0.134
Gender (female over male)	2.610	[-2.203, 7.422]	0.281
MAYSI II AI	-1.124	[-2.455, 0.207]	0.096
MAYSI II DA	0.845	[-0.339, 2.028]	0.157

Table 28: Results of the analysis for outcome CIS total with TAU as reference group. The results represent the pooled estimates with corresponding 95% confidence interval and p-value of the linear mixed effects model. The analysis was performed in the imputed FAS (imputations in accordance with specified methods).

Final Report

	Estimate	95% CI	p-vaue
Treatment (group over TAU)	0.659	[-2.05, 3.367]	0.622
Treatment (self over TAU)	-0.871	[-3.714, 1.972]	0.535
PHQ4 total at T1	0.666	[0.332, 0.999]	0.000
Age (years)	-0.189	[-0.897, 0.518]	0.588
Gender (female over male)	-0.539	[-2.793, 1.715]	0.628
Gender (diverse over male)	4.990	[-1.141, 11.121]	0.107
MAYSI II AI	-0.357	[-0.835, 0.121]	0.137
MAYSI II DA	0.107	[-0.426, 0.64]	0.682

Table 29: Results of the analysis for outcome PHQ4 total with TAU as reference group. The results represent the pooled estimates with corresponding 95% confidence interval and p-value of the linear mixed effects model. The analysis was performed in the imputed FAS (imputations in accordance with specified methods).

Final Report

	Estimate	95% CI	p-vaue
Treatment (group over TAU)	0.683	[-0.825, 2.192]	0.362
Treatment (self over TAU)	-0.214	[-1.819, 1.392]	0.788
PHQ4 anxiety at T1	0.452	[0.121, 0.783]	0.009
Age (years)	-0.037	[-0.457, 0.383]	0.859
Gender (female over male)	-0.436	[-1.759, 0.887]	0.506
Gender (diverse over male)	2.152	[-1.48, 5.784]	0.235
MAYSI II AI	-0.220	[-0.503, 0.062]	0.122
MAYSI II DA	0.160	[-0.152, 0.473]	0.303

Table 30: Results of the analysis for outcome PHQ4 anxiety with TAU as reference group. The results represent the pooled estimates with corresponding 95% confidence interval and p-value of the linear mixed effects model. The analysis was performed in the imputed FAS (imputations in accordance with specified methods).

	Estimate	95% CI	p-vaue
Treatment (group over TAU)	0.308	[-1.016, 1.632]	0.638
Treatment (self over TAU)	-0.319	[-1.716, 1.079]	0.645
PHQ4 depression at T1	0.664	[0.32, 1.009]	0.000
Age (years)	-0.098	[-0.465, 0.268]	0.588
Gender (female over male)	0.111	[-1.087, 1.308]	0.852
Gender (diverse over male)	3.229	[0.018, 6.44]	0.049
MAYSI II AI	-0.143	[-0.38, 0.095]	0.229
MAYSI II DA	-0.041	[-0.316, 0.233]	0.761

Table 31: Results of the analysis for outcome PHQ4 depression with TAU as reference group. The results represent the pooled estimates with corresponding 95% confidence interval and p-value of the linear mixed effects model. The analysis was performed in the imputed FAS (imputations in accordance with specified methods).

	Estimate	95% CI	p-vaue
Treatment (group over TAU)	-0.625	[-2.725, 1.475]	0.557
Treatment (self over TAU)	-0.865	[-3.018, 1.287]	0.427
SWE total at T1	0.120	[-0.038, 0.279]	0.135
Age (years)	-0.050	[-0.676, 0.576]	0.875
Gender (female over male)	1.223	[-0.715, 3.16]	0.213
Gender (diverse over male)	-0.607	[-11.446, 10.231]	0.912
MAYSI II AI	0.118	[-0.318, 0.554]	0.592
MAYSI II DA	0.069	[-0.38, 0.517]	0.762

Table 32: Results of the analysis for outcome SWE total with TAU as reference group. The results represent the pooled estimates with corresponding 95% confidence interval and p-value of the linear mixed effects model. The analysis was performed in the imputed FAS (imputations in accordance with specified methods).

	Estimate	95% CI	p-vaue
Treatment (group over TAU)	-0.205	[-2.341, 1.93]	0.847
Treatment (self over TAU)	1.436	[-1.199, 4.072]	0.278
ARI total at T1	0.468	[0.191, 0.745]	0.001
Age (years)	0.088	[-0.523, 0.699]	0.773
Gender (female over male)	0.769	[-1.062, 2.601]	0.402
MAYSI II AI	-0.187	[-0.72, 0.347]	0.483
MAYSI II DA	-0.116	[-0.531, 0.3]	0.577

Table 33: Results of the analysis for outcome ARI total with TAU as reference group. The results represent the pooled estimates with corresponding 95% confidence interval and p-value of the linear mixed effects model. The analysis was performed in the imputed FAS (imputations in accordance with specified methods).

	Estimate	95% CI	p-vaue
Treatment (group over TAU)	0.413	[-2.16, 2.986]	0.747
Treatment (self over TAU)	-1.292	[-4.514, 1.931]	0.422
EssenCES SE at T1	0.533	[0.291, 0.774]	0.000
Age (years)	-0.365	[-1.148, 0.418]	0.352
Gender (female over male)	0.366	[-1.892, 2.624]	0.745
MAYSI II AI	0.154	[-0.549, 0.857]	0.659
MAYSI II DA	-0.034	[-0.588, 0.521]	0.902

Table 34: Results of the analysis for outcome EssenCES SE with TAU as reference group. The results represent the pooled estimates with corresponding 95% confidence interval and p-value of the linear mixed effects model. The analysis was performed in the imputed FAS (imputations in accordance with specified methods).

	Estimate	95% CI	p-vaue
Treatment (group over TAU)	-0.422	[-2.76, 1.917]	0.718
Treatment (self over TAU)	1.181	[-1.798, 4.161]	0.427
EssenCES ZP at T1	0.429	[0.242, 0.617]	0.000
Age (years)	-0.130	[-0.819, 0.558]	0.704
Gender (female over male)	-1.066	[-3.182, 1.05]	0.314
MAYSI II AI	0.044	[-0.569, 0.656]	0.886
MAYSI II DA	0.237	[-0.251, 0.726]	0.332

Table 35: Results of the analysis for outcome EssenCES ZP with TAU as reference group. The results represent the pooled estimates with corresponding 95% confidence interval and p-value of the linear mixed effects model. The analysis was performed in the imputed FAS (imputations in accordance with specified methods).

Final Report

	Estimate	95% CI	p-vaue
Treatment (group over TAU)	1.227	[-0.951, 3.405]	0.262
Treatment (self over TAU)	-0.178	[-2.994, 2.639]	0.899
EssenCES TH at T1	0.455	[0.237, 0.673]	0.000
Age (years)	0.192	[-0.478, 0.862]	0.566
Gender (female over male)	0.694	[-1.333, 2.721]	0.493
MAYSI II AI	0.515	[-0.073, 1.102]	0.084
MAYSI II DA	-0.223	[-0.709, 0.262]	0.358

Table 36: Results of the analysis for outcome EssenCES TH with TAU as reference group. The results represent the pooled estimates with corresponding 95% confidence interval and p-value of the linear mixed effects model. The analysis was performed in the imputed FAS (imputations in accordance with specified methods).

	Estimate	95% CI	p-vaue
Treatment (group over TAU)	-1.516	[-7.622, 4.59]	0.621
Treatment (self over TAU)	-7.586	[-13.766, -1.406]	0.017
CD-RISC caretaker total at T1	0.220	[0.042, 0.398]	0.016
Age (years)	-0.625	[-1.895, 0.646]	0.329
Gender (female over male)	1.708	[-2.131, 5.547]	0.377
MAYSI II AI	-0.673	[-1.368, 0.021]	0.057
MAYSI II DA	0.793	[0.129, 1.457]	0.020

Table 37: Results of the analysis for outcome CD-RISC total caretaker with TAU as reference group. The results represent the pooled estimates with corresponding 95% confidence interval and p-value of the linear mixed effects model. The analysis was performed in the imputed FAS (imputations in accordance with specified methods).

	Estimate	95% CI	p-vaue
Treatment (group over TAU)	0.187	[-5.281, 5.655]	0.945
Treatment (self over TAU)	4.839	[-1.965, 11.643]	0.159
CIS total at T1	0.278	[-0.009, 0.564]	0.057
Age (years)	1.226	[-0.39, 2.841]	0.134
Gender (female over male)	2.610	[-2.203, 7.422]	0.281
MAYSI II AI	-1.124	[-2.455, 0.207]	0.096
MAYSI II DA	0.845	[-0.339, 2.028]	0.157

Table 38: Results of the analysis for outcome CIS total with TAU as reference group. The results represent the pooled estimates with corresponding 95% confidence interval and p-value of the linear mixed effects model. The analysis was performed in the imputed FAS (imputations in accordance with specified methods).

	Estimate	95% CI	p-vaue
Treatment (group over TAU)	-0.780	[-2.474, 0.914]	0.361
Treatment (self over TAU)	-0.923	[-2.613, 0.767]	0.279
ARI caretaker total at T1	0.480	[0.231, 0.73]	0.000
Age (years)	-0.346	[-0.928, 0.235]	0.239
Gender (female over male)	0.614	[-0.948, 2.176]	0.435
MAYSI II AI	-0.183	[-0.548, 0.182]	0.320
MAYSI II DA	0.036	[-0.313, 0.384]	0.839

Table 39: Results of the analysis for outcome ARI total caretaker with TAU as reference group. The results represent the pooled estimates with corresponding 95% confidence interval and p-value of the linear mixed effects model. The analysis was performed in the imputed FAS (imputations in accordance with specified methods).

Final Report

Final Report

6.1.2 Self-guided training as reference

	Estimate	95% CI	p-vaue
Treatment (group over self)	-0.933	[-3.819, 1.953]	0.520
Treatment (TAU over self)	0.691	[-2.533, 3.915]	0.670
CD-RISC total at T1	0.038	[-0.133, 0.209]	0.658
Age (years)	0.280	[-0.501, 1.062]	0.475
Gender (female over male)	0.825	[-1.692, 3.342]	0.514
Gender (diverse over male)	-1.486	[-13.164, 10.192]	0.800
MAYSI II AI	0.248	[-0.346, 0.841]	0.406
MAYSI II DA	-0.141	[-0.767, 0.486]	0.654

Table 40: Results of the analysis for outcome CD-RISC total with self-helped training as reference group. The results represent the pooled estimates with corresponding 95% confidence interval and p-value of the linear mixed effects model. The analysis was performed in the imputed FAS (imputations in accordance with specified methods).

	Estimate	95% CI	p-vaue
Treatment (group over self)	3.690	[-0.108, 7.488]	0.057
Treatment (TAU over self)	2.546	[-1.516, 6.608]	0.213
WHO5 total at T1	0.294	[0.056, 0.531]	0.017
Age (years)	-0.489	[-1.459, 0.481]	0.316
Gender (female over male)	-1.944	[-4.778, 0.891]	0.174
MAYSI II AI	-0.295	[-1.104, 0.514]	0.466
MAYSI II DA	-0.519	[-1.221, 0.183]	0.143

Table 41: Results of the analysis for outcome WHO5 total with self-helped training as reference group. The results represent the pooled estimates with corresponding 95% confidence interval and p-value of the linear mixed effects model. The analysis was performed in the imputed FAS (imputations in accordance with specified methods).

	Estimate	95% CI	p-vaue
Treatment (group over self)	-4.652	[-11.056, 1.753]	0.150
Treatment (TAU over self)	-4.839	[-11.643, 1.965]	0.159
CIS total at T1	0.278	[-0.009, 0.564]	0.057
Age (years)	1.226	[-0.39, 2.841]	0.134
Gender (female over male)	2.610	[-2.203, 7.422]	0.281
MAYSI II AI	-1.124	[-2.455, 0.207]	0.096
MAYSI II DA	0.845	[-0.339, 2.028]	0.157

Table 42: Results of the analysis for outcome CIS total with self-helped training as reference group. The results represent the pooled estimates with corresponding 95% confidence interval and p-value of the linear mixed effects model. The analysis was performed in the imputed FAS (imputations in accordance with specified methods).

6.2 Full tables of the secondary estimand: per protocol analysis

The tables below present the results of the secondary analyses performed in the per protocol analysis set, including the estimates for all covariates in the models.

6.2.1 TAU as reference

	Estimate	95% CI	p-vaue
Treatment (group over self)	1.529	[-1.04, 4.098]	0.233
Treatment (TAU over self)	0.871	[-1.972, 3.714]	0.535
PHQ4 total at T1	0.666	[0.332, 0.999]	0.000
Age (years)	-0.189	[-0.897, 0.518]	0.588
Gender (female over male)	-0.539	[-2.793, 1.715]	0.628
Gender (diverse over male)	4.990	[-1.141, 11.121]	0.107
MAYSI II AI	-0.357	[-0.835, 0.121]	0.137
MAYSI II DA	0.107	[-0.426, 0.64]	0.682

Table 43: Results of the analysis for outcome PHQ4 total with self-helped training as reference group. The results represent the pooled estimates with corresponding 95% confidence interval and p-value of the linear mixed effects model. The analysis was performed in the imputed FAS (imputations in accordance with specified methods).

	Estimate	95% CI	p-vaue
Treatment (group over self)	0.897	[-0.561, 2.355]	0.218
Treatment (TAU over self)	0.214	[-1.392, 1.819]	0.788
PHQ4 anxiety at T1	0.452	[0.121, 0.783]	0.009
Age (years)	-0.037	[-0.457, 0.383]	0.859
Gender (female over male)	-0.436	[-1.759, 0.887]	0.506
Gender (diverse over male)	2.152	[-1.48, 5.784]	0.235
MAYSI II AI	-0.220	[-0.503, 0.062]	0.122
MAYSI II DA	0.160	[-0.152, 0.473]	0.303

Table 44: Results of the analysis for outcome PHQ4 anxiety with self-helped training as reference group. The results represent the pooled estimates with corresponding 95% confidence interval and p-value of the linear mixed effects model. The analysis was performed in the imputed FAS (imputations in accordance with specified methods).

	Estimate	95% CI	p-vaue
Treatment (group over self)	0.627	[-0.631, 1.885]	0.317
Treatment (TAU over self)	0.319	[-1.079, 1.716]	0.645
PHQ4 depression at T1	0.664	[0.32, 1.009]	0.000
Age (years)	-0.098	[-0.465, 0.268]	0.588
Gender (female over male)	0.111	[-1.087, 1.308]	0.852
Gender (diverse over male)	3.229	[0.018, 6.44]	0.049
MAYSI II AI	-0.143	[-0.38, 0.095]	0.229
MAYSI II DA	-0.041	[-0.316, 0.233]	0.761

Table 45: Results of the analysis for outcome PHQ4 depression with self-helped training as reference group. The results represent the pooled estimates with corresponding 95% confidence interval and p-value of the linear mixed effects model. The analysis was performed in the imputed FAS (imputations in accordance with specified methods).

Final Report

	Estimate	95% CI	p-vaue
Treatment (group over self)	0.241	[-1.737, 2.218]	0.810
Treatment (TAU over self)	0.865	[-1.287, 3.018]	0.427
SWE total at T1	0.120	[-0.038, 0.279]	0.135
Age (years)	-0.050	[-0.676, 0.576]	0.875
Gender (female over male)	1.223	[-0.715, 3.16]	0.213
Gender (diverse over male)	-0.607	[-11.446, 10.231]	0.912
MAYSI II AI	0.118	[-0.318, 0.554]	0.592
MAYSI II DA	0.069	[-0.38, 0.517]	0.762

Table 46: Results of the analysis for outcome SWE total with self-helped training as reference group. The results represent the pooled estimates with corresponding 95% confidence interval and p-value of the linear mixed effects model. The analysis was performed in the imputed FAS (imputations in accordance with specified methods).

	Estimate	95% CI	p-vaue
Treatment (group over self)	-1.642	[-4.14, 0.856]	0.192
Treatment (TAU over self)	-1.436	[-4.072, 1.199]	0.278
ARI total at T1	0.468	[0.191, 0.745]	0.001
Age (years)	0.088	[-0.523, 0.699]	0.773
Gender (female over male)	0.769	[-1.062, 2.601]	0.402
MAYSI II AI	-0.187	[-0.72, 0.347]	0.483
MAYSI II DA	-0.116	[-0.531, 0.3]	0.577

Table 47: Results of the analysis for outcome ARI total with self-helped training as reference group. The results represent the pooled estimates with corresponding 95% confidence interval and p-value of the linear mixed effects model. The analysis was performed in the imputed FAS (imputations in accordance with specified methods).

	Estimate	95% CI	p-vaue
Treatment (group over self)	1.705	[-1.451, 4.861]	0.280
Treatment (TAU over self)	1.292	[-1.931, 4.514]	0.422
EssenCES SE at T1	0.533	[0.291, 0.774]	0.000
Age (years)	-0.365	[-1.148, 0.418]	0.352
Gender (female over male)	0.366	[-1.892, 2.624]	0.745
MAYSI II AI	0.154	[-0.549, 0.857]	0.659
MAYSI II DA	-0.034	[-0.588, 0.521]	0.902

Table 48: Results of the analysis for outcome EssenCES SE with self-helped training as reference group. The results represent the pooled estimates with corresponding 95% confidence interval and p-value of the linear mixed effects model. The analysis was performed in the imputed FAS (imputations in accordance with specified methods).

	Estimate	95% CI	p-vaue
Treatment (group over self)	-1.603	[-4.409, 1.204]	0.254
Treatment (TAU over self)	-1.181	[-4.161, 1.798]	0.427
EssenCES ZP at T1	0.429	[0.242, 0.617]	0.000
Age (years)	-0.130	[-0.819, 0.558]	0.704
Gender (female over male)	-1.066	[-3.182, 1.05]	0.314
MAYSI II AI	0.044	[-0.569, 0.656]	0.886
MAYSI II DA	0.237	[-0.251, 0.726]	0.332

Table 49: Results of the analysis for outcome EssenCES ZP with self-helped training as reference group. The results represent the pooled estimates with corresponding 95% confidence interval and p-value of the linear mixed effects model. The analysis was performed in the imputed FAS (imputations in accordance with specified methods).

Final Report

	Estimate	95% CI	p-vaue
Treatment (group over self)	1.405	[-1.436, 4.246]	0.321
Treatment (TAU over self)	0.178	[-2.639, 2.994]	0.899
EssenCES TH at T1	0.455	[0.237, 0.673]	0.000
Age (years)	0.192	[-0.478, 0.862]	0.566
Gender (female over male)	0.694	[-1.333, 2.721]	0.493
MAYSI II AI	0.515	[-0.073, 1.102]	0.084
MAYSI II DA	-0.223	[-0.709, 0.262]	0.358

Table 50: Results of the analysis for outcome EssenCES TH with self-helped training as reference group. The results represent the pooled estimates with corresponding 95% confidence interval and p-value of the linear mixed effects model. The analysis was performed in the imputed FAS (imputations in accordance with specified methods).

	Estimate	95% CI	p-vaue
Treatment (group over self)	6.070	[0.077, 12.063]	0.047
Treatment (TAU over self)	7.586	[1.406, 13.766]	0.017
CD-RISC caretaker total at T1	0.220	[0.042, 0.398]	0.016
Age (years)	-0.625	[-1.895, 0.646]	0.329
Gender (female over male)	1.708	[-2.131, 5.547]	0.377
MAYSI II AI	-0.673	[-1.368, 0.021]	0.057
MAYSI II DA	0.793	[0.129, 1.457]	0.020

Table 51: Results of the analysis for outcome CD-RISC total caretaker with self-helped training as reference group. The results represent the pooled estimates with corresponding 95% confidence interval and p-value of the linear mixed effects model. The analysis was performed in the imputed FAS (imputations in accordance with specified methods).

	Estimate	95% CI	p-vaue
Treatment (group over self)	-4.652	[-11.056, 1.753]	0.150
Treatment (TAU over self)	-4.839	[-11.643, 1.965]	0.159
CIS total at T1	0.278	[-0.009, 0.564]	0.057
Age (years)	1.226	[-0.39, 2.841]	0.134
Gender (female over male)	2.610	[-2.203, 7.422]	0.281
MAYSI II AI	-1.124	[-2.455, 0.207]	0.096
MAYSI II DA	0.845	[-0.339, 2.028]	0.157

Table 52: Results of the analysis for outcome CIS total with self-helped training as reference group. The results represent the pooled estimates with corresponding 95% confidence interval and p-value of the linear mixed effects model. The analysis was performed in the imputed FAS (imputations in accordance with specified methods).

	Estimate	95% CI	p-vaue
Treatment (group over self)	0.143	[-1.442, 1.728]	0.858
Treatment (TAU over self)	0.923	[-0.767, 2.613]	0.279
ARI caretaker total at T1	0.480	[0.231, 0.73]	0.000
Age (years)	-0.346	[-0.928, 0.235]	0.239
Gender (female over male)	0.614	[-0.948, 2.176]	0.435
MAYSI II AI	-0.183	[-0.548, 0.182]	0.320
MAYSI II DA	0.036	[-0.313, 0.384]	0.839

Table 53: Results of the analysis for outcome ARI total caretaker with self-helped training as reference group. The results represent the pooled estimates with corresponding 95% confidence interval and p-value of the linear mixed effects model. The analysis was performed in the imputed FAS (imputations in accordance with specified methods).

Final Report

Final Report

	Estimate	95% CI	p-vaue
Treatment (self over TAU)	-0.378	[-8.701, 7.946]	0.928
randotau	1.557	[-4.927, 8.042]	0.631
AFQ total at T1	0.394	[0.138, 0.651]	0.003
Age (years)	0.197	[-1.7, 2.093]	0.836
Gender (female over male)	1.393	[-4.844, 7.631]	0.656
Gender (diverse over male)	2.108	[-31.531, 35.748]	0.900
MAYSI II AI	-1.363	[-2.808, 0.082]	0.064
MAYSI II DA	0.067	[-1.4, 1.533]	0.928

Table 54: Results of the analysis for outcome AFQ total with TAU as reference group. The results represent the pooled estimates with corresponding 95% confidence interval and p-value of the linear mixed effects model. The analysis was performed in the imputed PP analysis set (imputations in accordance with specified methods).

	Estimate	95% CI	p-vaue
Treatment (self over TAU)	1.399	[-2.568, 5.365]	0.482
randotau	1.661	[-1.606, 4.928]	0.312
CD-RISC total at T1	0.060	[-0.156, 0.276]	0.576
Age (years)	0.324	[-0.548, 1.196]	0.458
Gender (female over male)	1.011	[-1.945, 3.967]	0.495
Gender (diverse over male)	-1.407	[-13.713, 10.898]	0.819
MAYSI II AI	0.287	[-0.388, 0.961]	0.396
MAYSI II DA	-0.182	[-0.891, 0.527]	0.609

Table 55: Results of the analysis for outcome CD-RISC total with TAU as reference group. The results represent the pooled estimates with corresponding 95% confidence interval and p-value of the linear mixed effects model. The analysis was performed in the imputed PP analysis set (imputations in accordance with specified methods).

	Estimate	95% CI	p-vaue
Treatment (self over TAU)	-3.648	[-8.433, 1.137]	0.131
randotau	-1.613	[-5.444, 2.218]	0.399
WHO5 total at T1	0.387	[0.118, 0.656]	0.006
Age (years)	-0.398	[-1.465, 0.669]	0.455
Gender (female over male)	-1.445	[-4.614, 1.724]	0.362
MAYSI II AI	-0.217	[-1.138, 0.704]	0.635
MAYSI II DA	-0.394	[-1.146, 0.358]	0.295

Table 56: Results of the analysis for outcome WHO5 total with TAU as reference group. The results represent the pooled estimates with corresponding 95% confidence interval and p-value of the linear mixed effects model. The analysis was performed in the imputed PP analysis set (imputations in accordance with specified methods).

	Estimate	95% CI	p-vaue
Treatment (self over TAU)	2.486	[-5.157, 10.129]	0.514
randotau	0.426	[-5.513, 6.364]	0.885
CIS total at T1	0.335	[0.003, 0.667]	0.048
Age (years)	1.111	[-0.629, 2.851]	0.204
Gender (female over male)	2.018	[-3.276, 7.313]	0.445
MAYSI II AI	-1.306	[-2.86, 0.247]	0.097
MAYSI II DA	1.031	[-0.258, 2.32]	0.114

Table 57: Results of the analysis for outcome CIS total with TAU as reference group. The results represent the pooled estimates with corresponding 95% confidence interval and p-value of the linear mixed effects model. The analysis was performed in the imputed PP analysis set (imputations in accordance with specified methods).

Final Report

	Estimate	95% CI	p-vaue
Treatment (self over TAU)	0.981	[-3.441, 5.403]	0.640
randotau	-0.995	[-4.011, 2.02]	0.489
PHQ4 total at T1	0.662	[0.246, 1.077]	0.004
Age (years)	-0.068	[-0.984, 0.848]	0.875
Gender (female over male)	-0.117	[-2.945, 2.711]	0.930
Gender (diverse over male)	4.454	[-1.638, 10.546]	0.139
MAYSI II AI	0.039	[-0.684, 0.762]	0.909
MAYSI II DA	-0.229	[-0.891, 0.433]	0.469

Table 58: Results of the analysis for outcome PHQ4 total with TAU as reference group. The results represent the pooled estimates with corresponding 95% confidence interval and p-value of the linear mixed effects model. The analysis was performed in the imputed PP analysis set (imputations in accordance with specified methods).

	Estimate	95% CI	p-vaue
Treatment (self over TAU)	0.816	[-1.765, 3.397]	0.511
randotau	-0.825	[-2.435, 0.785]	0.292
PHQ4 anxiety at T1	0.433	[0.023, 0.843]	0.040
Age (years)	-0.076	[-0.643, 0.49]	0.779
Gender (female over male)	-0.365	[-2.006, 1.276]	0.643
Gender (diverse over male)	1.900	[-1.806, 5.605]	0.292
MAYSI II AI	0.043	[-0.399, 0.485]	0.837
MAYSI II DA	-0.047	[-0.441, 0.346]	0.802

Table 59: Results of the analysis for outcome PHQ4 anxiety with TAU as reference group. The results represent the pooled estimates with corresponding 95% confidence interval and p-value of the linear mixed effects model. The analysis was performed in the imputed PP analysis set (imputations in accordance with specified methods).

	Estimate	95% CI	p-vaue
Treatment (self over TAU)	0.421	[-2.155, 2.997]	0.732
randotau	-0.453	[-2.055, 1.15]	0.555
PHQ4 depression at T1	0.678	[0.16, 1.197]	0.014
Age (years)	-0.032	[-0.584, 0.52]	0.902
Gender (female over male)	0.455	[-1.185, 2.096]	0.562
Gender (diverse over male)	3.089	[-0.468, 6.646]	0.084
MAYSI II AI	-0.005	[-0.439, 0.428]	0.979
MAYSI II DA	-0.110	[-0.485, 0.264]	0.538

Table 60: Results of the analysis for outcome PHQ4 depression with TAU as reference group. The results represent the pooled estimates with corresponding 95% confidence interval and p-value of the linear mixed effects model. The analysis was performed in the imputed PP analysis set (imputations in accordance with specified methods).

	Estimate	95% CI	p-vaue
Treatment (self over TAU)	-0.046	[-3.255, 3.163]	0.977
randotau	0.154	[-2.245, 2.553]	0.899
SWE total at T1	0.180	[-0.029, 0.389]	0.090
Age (years)	-0.046	[-0.783, 0.691]	0.901
Gender (female over male)	1.486	[-0.936, 3.907]	0.225
Gender (diverse over male)	-0.805	[-12.538, 10.929]	0.891
MAYSI II AI	0.116	[-0.464, 0.697]	0.689
MAYSI II DA	0.100	[-0.47, 0.671]	0.727

Table 61: Results of the analysis for outcome SWE total with TAU as reference group. The results represent the pooled estimates with corresponding 95% confidence interval and p-value of the linear mixed effects model. The analysis was performed in the imputed PP analysis set (imputations in accordance with specified methods).

	Estimate	95% CI	p-vaue
Treatment (self over TAU)	1.191	[-1.771, 4.153]	0.420
randotau	0.411	[-1.946, 2.769]	0.726
ARI total at T1	0.434	[0.125, 0.742]	0.007
Age (years)	0.072	[-0.575, 0.72]	0.822
Gender (female over male)	0.404	[-1.588, 2.396]	0.683
MAYSI II AI	-0.231	[-0.843, 0.382]	0.449
MAYSI II DA	-0.103	[-0.572, 0.367]	0.660

Table 62: Results of the analysis for outcome ARI total with TAU as reference group. The results represent the pooled estimates with corresponding 95% confidence interval and p-value of the linear mixed effects model. The analysis was performed in the imputed PP analysis set (imputations in accordance with specified methods).

	Estimate	95% CI	p-vaue
Treatment (self over TAU)	0.395	[-2.837, 3.628]	0.805
randotau	-0.474	[-3.198, 2.25]	0.724
EssenCES SE at T1	0.532	[0.268, 0.795]	0.000
Age (years)	-0.341	[-1.075, 0.394]	0.352
Gender (female over male)	1.167	[-1.031, 3.365]	0.288
MAYSI II AI	0.330	[-0.369, 1.028]	0.341
MAYSI II DA	-0.200	[-0.748, 0.348]	0.463

Table 63: Results of the analysis for outcome EssenCES SE with TAU as reference group. The results represent the pooled estimates with corresponding 95% confidence interval and p-value of the linear mixed effects model. The analysis was performed in the imputed PP analysis set (imputations in accordance with specified methods).

	Estimate	95% CI	p-vaue
Treatment (self over TAU)	1.020	[-2.137, 4.177]	0.515
randotau	0.389	[-2.154, 2.932]	0.757
EssenCES ZP at T1	0.489	[0.288, 0.69]	0.000
Age (years)	-0.104	[-0.82, 0.613]	0.771
Gender (female over male)	-1.260	[-3.518, 0.999]	0.264
MAYSI II AI	-0.030	[-0.715, 0.655]	0.929
MAYSI II DA	0.315	[-0.232, 0.862]	0.250

Table 64: Results of the analysis for outcome EssenCES ZP with TAU as reference group. The results represent the pooled estimates with corresponding 95% confidence interval and p-value of the linear mixed effects model. The analysis was performed in the imputed PP analysis set (imputations in accordance with specified methods).

Final Report

	Estimate	95% CI	p-vaue
Treatment (self over TAU)	-1.259	[-4.349, 1.831]	0.413
randotau	-1.216	[-3.506, 1.073]	0.287
EssenCES TH at T1	0.550	[0.317, 0.782]	0.000
Age (years)	0.124	[-0.543, 0.791]	0.709
Gender (female over male)	0.669	[-1.409, 2.748]	0.516
MAYSI II AI	0.551	[-0.069, 1.171]	0.080
MAYSI II DA	-0.249	[-0.749, 0.251]	0.319

Table 65: Results of the analysis for outcome EssenCES TH with TAU as reference group. The results represent the pooled estimates with corresponding 95% confidence interval and p-value of the linear mixed effects model. The analysis was performed in the imputed PP analysis set (imputations in accordance with specified methods).

	Estimate	95% CI	p-vaue
Treatment (self over TAU)	-5.060	[-13.077, 2.957]	0.209
randotau	-0.099	[-6.764, 6.567]	0.976
CD-RISC caretaker total at T1	0.230	[0.02, 0.44]	0.033
Age (years)	-0.342	[-1.715, 1.03]	0.617
Gender (female over male)	1.366	[-2.548, 5.28]	0.485
MAYSI II AI	-0.113	[-0.945, 0.719]	0.785
MAYSI II DA	0.494	[-0.207, 1.196]	0.162

Table 66: Results of the analysis for outcome CD-RISC total caretaker with TAU as reference group. The results represent the pooled estimates with corresponding 95% confidence interval and p-value of the linear mixed effects model. The analysis was performed in the imputed PP analysis set (imputations in accordance with specified methods).

	Estimate	95% CI	p-vaue
Treatment (self over TAU)	2.486	[-5.157, 10.129]	0.514
randotau	0.426	[-5.513, 6.364]	0.885
CIS total at T1	0.335	[0.003, 0.667]	0.048
Age (years)	1.111	[-0.629, 2.851]	0.204
Gender (female over male)	2.018	[-3.276, 7.313]	0.445
MAYSI II AI	-1.306	[-2.86, 0.247]	0.097
MAYSI II DA	1.031	[-0.258, 2.32]	0.114

Table 67: Results of the analysis for outcome CIS total with TAU as reference group. The results represent the pooled estimates with corresponding 95% confidence interval and p-value of the linear mixed effects model. The analysis was performed in the imputed PP analysis set (imputations in accordance with specified methods).

	Estimate	95% CI	p-vaue
Treatment (self over TAU)	0.050	[-2.599, 2.7]	0.970
randotau	0.499	[-1.486, 2.484]	0.615
ARI caretaker total at T1	0.455	[0.111, 0.799]	0.011
Age (years)	-0.260	[-1.018, 0.497]	0.492
Gender (female over male)	1.132	[-0.833, 3.098]	0.252
MAYSI II AI	-0.050	[-0.569, 0.468]	0.845
MAYSI II DA	0.061	[-0.372, 0.494]	0.777

Table 68: Results of the analysis for outcome ARI total caretaker with TAU as reference group. The results represent the pooled estimates with corresponding 95% confidence interval and p-value of the linear mixed effects model. The analysis was performed in the imputed PP analysis set (imputations in accordance with specified methods).

Final Report

6.2.2 Self-guided training as reference

	Estimate	95% CI	p-vaue
Treatment (group over self)	0.378	[-7.946, 8.701]	0.928
Treatment (TAU over self)	1.935	[-6.428, 10.299]	0.645
AFQ total at T1	0.394	[0.138, 0.651]	0.003
Age (years)	0.197	[-1.7, 2.093]	0.836
Gender (female over male)	1.393	[-4.844, 7.631]	0.656
Gender (diverse over male)	2.108	[-31.531, 35.748]	0.900
MAYSI II AI	-1.363	[-2.808, 0.082]	0.064
MAYSI II DA	0.067	[-1.4, 1.533]	0.928

Table 69: Results of the analysis for outcome AFQ total with self-helped training as reference group. The results represent the pooled estimates with corresponding 95% confidence interval and p-value of the linear mixed effects model. The analysis was performed in the imputed PP analysis set (imputations in accordance with specified methods).

	Estimate	95% CI	p-vaue
Treatment (group over self)	-1.399	[-5.365, 2.568]	0.482
Treatment (TAU over self)	0.263	[-3.742, 4.267]	0.896
CD-RISC total at T1	0.060	[-0.156, 0.276]	0.576
Age (years)	0.324	[-0.548, 1.196]	0.458
Gender (female over male)	1.011	[-1.945, 3.967]	0.495
Gender (diverse over male)	-1.407	[-13.713, 10.898]	0.819
MAYSI II AI	0.287	[-0.388, 0.961]	0.396
MAYSI II DA	-0.182	[-0.891, 0.527]	0.609

Table 70: Results of the analysis for outcome CD-RISC total with self-helped training as reference group. The results represent the pooled estimates with corresponding 95% confidence interval and p-value of the linear mixed effects model. The analysis was performed in the imputed PP analysis set (imputations in accordance with specified methods).

Final Report

Final Report

	Estimate	95% CI	p-vaue
Treatment (group over self)	3.648	[-1.137, 8.433]	0.131
Treatment (TAU over self)	2.034	[-3.116, 7.185]	0.429
WHO5 total at T1	0.387	[0.118, 0.656]	0.006
Age (years)	-0.398	[-1.465, 0.669]	0.455
Gender (female over male)	-1.445	[-4.614, 1.724]	0.362
MAYSI II AI	-0.217	[-1.138, 0.704]	0.635
MAYSI II DA	-0.394	[-1.146, 0.358]	0.295

Table 71: Results of the analysis for outcome WHO5 total with self-helped training as reference group. The results represent the pooled estimates with corresponding 95% confidence interval and p-value of the linear mixed effects model. The analysis was performed in the imputed PP analysis set (imputations in accordance with specified methods).

	Estimate	95% CI	p-vaue
Treatment (group over self)	-2.486	[-10.129, 5.157]	0.514
Treatment (TAU over self)	-2.061	[-10.298, 6.177]	0.615
CIS total at T1	0.335	[0.003, 0.667]	0.048
Age (years)	1.111	[-0.629, 2.851]	0.204
Gender (female over male)	2.018	[-3.276, 7.313]	0.445
MAYSI II AI	-1.306	[-2.86, 0.247]	0.097
MAYSI II DA	1.031	[-0.258, 2.32]	0.114

Table 72: Results of the analysis for outcome CIS total with self-helped training as reference group. The results represent the pooled estimates with corresponding 95% confidence interval and p-value of the linear mixed effects model. The analysis was performed in the imputed PP analysis set (imputations in accordance with specified methods).

	Estimate	95% CI	p-vaue
Treatment (group over self)	-0.981	[-5.403, 3.441]	0.640
Treatment (TAU over self)	-1.976	[-6.56, 2.607]	0.369
PHQ4 total at T1	0.662	[0.246, 1.077]	0.004
Age (years)	-0.068	[-0.984, 0.848]	0.875
Gender (female over male)	-0.117	[-2.945, 2.711]	0.930
Gender (diverse over male)	4.454	[-1.638, 10.546]	0.139
MAYSI II AI	0.039	[-0.684, 0.762]	0.909
MAYSI II DA	-0.229	[-0.891, 0.433]	0.469

Table 73: Results of the analysis for outcome PHQ4 total with self-helped training as reference group. The results represent the pooled estimates with corresponding 95% confidence interval and p-value of the linear mixed effects model. The analysis was performed in the imputed PP analysis set (imputations in accordance with specified methods).

	Estimate	95% CI	p-vaue
Treatment (group over self)	-0.816	[-3.397, 1.765]	0.511
Treatment (TAU over self)	-1.642	[-4.307, 1.024]	0.209
PHQ4 anxiety at T1	0.433	[0.023, 0.843]	0.040
Age (years)	-0.076	[-0.643, 0.49]	0.779
Gender (female over male)	-0.365	[-2.006, 1.276]	0.643
Gender (diverse over male)	1.900	[-1.806, 5.605]	0.292
MAYSI II AI	0.043	[-0.399, 0.485]	0.837
MAYSI II DA	-0.047	[-0.441, 0.346]	0.802

Table 74: Results of the analysis for outcome PHQ4 anxiety with self-helped training as reference group. The results represent the pooled estimates with corresponding 95% confidence interval and p-value of the linear mixed effects model. The analysis was performed in the imputed PP analysis set (imputations in accordance with specified methods).

	Estimate	95% CI	p-vaue
Treatment (group over self)	-0.421	[-2.997, 2.155]	0.732
Treatment (TAU over self)	-0.873	[-3.562, 1.815]	0.498
PHQ4 depression at T1	0.678	[0.16, 1.197]	0.014
Age (years)	-0.032	[-0.584, 0.52]	0.902
Gender (female over male)	0.455	[-1.185, 2.096]	0.562
Gender (diverse over male)	3.089	[-0.468, 6.646]	0.084
MAYSI II AI	-0.005	[-0.439, 0.428]	0.979
MAYSI II DA	-0.110	[-0.485, 0.264]	0.538

Table 75: Results of the analysis for outcome PHQ4 depression with self-helped training as reference group. The results represent the pooled estimates with corresponding 95% confidence interval and p-value of the linear mixed effects model. The analysis was performed in the imputed PP analysis set (imputations in accordance with specified methods).

	Estimate	95% CI	p-vaue
Treatment (group over self)	0.046	[-3.163, 3.255]	0.977
Treatment (TAU over self)	0.200	[-3.11, 3.51]	0.904
SWE total at T1	0.180	[-0.029, 0.389]	0.090
Age (years)	-0.046	[-0.783, 0.691]	0.901
Gender (female over male)	1.486	[-0.936, 3.907]	0.225
Gender (diverse over male)	-0.805	[-12.538, 10.929]	0.891
MAYSI II AI	0.116	[-0.464, 0.697]	0.689
MAYSI II DA	0.100	[-0.47, 0.671]	0.727

Table 76: Results of the analysis for outcome SWE total with self-helped training as reference group. The results represent the pooled estimates with corresponding 95% confidence interval and p-value of the linear mixed effects model. The analysis was performed in the imputed PP analysis set (imputations in accordance with specified methods).

	Estimate	95% CI	p-vaue
Treatment (group over self)	-1.191	[-4.153, 1.771]	0.420
Treatment (TAU over self)	-0.780	[-3.933, 2.374]	0.619
ARI total at T1	0.434	[0.125, 0.742]	0.007
Age (years)	0.072	[-0.575, 0.72]	0.822
Gender (female over male)	0.404	[-1.588, 2.396]	0.683
MAYSI II AI	-0.231	[-0.843, 0.382]	0.449
MAYSI II DA	-0.103	[-0.572, 0.367]	0.660

Table 77: Results of the analysis for outcome ARI total with self-helped training as reference group. The results represent the pooled estimates with corresponding 95% confidence interval and p-value of the linear mixed effects model. The analysis was performed in the imputed PP analysis set (imputations in accordance with specified methods).

	Estimate	95% CI	p-vaue
Treatment (group over self)	-0.395	[-3.628, 2.837]	0.805
Treatment (TAU over self)	-0.869	[-4.368, 2.63]	0.616
EssenCES SE at T1	0.532	[0.268, 0.795]	0.000
Age (years)	-0.341	[-1.075, 0.394]	0.352
Gender (female over male)	1.167	[-1.031, 3.365]	0.288
MAYSI II AI	0.330	[-0.369, 1.028]	0.341
MAYSI II DA	-0.200	[-0.748, 0.348]	0.463

Table 78: Results of the analysis for outcome EssenCES SE with self-helped training as reference group. The results represent the pooled estimates with corresponding 95% confidence interval and p-value of the linear mixed effects model. The analysis was performed in the imputed PP analysis set (imputations in accordance with specified methods).

Final Report

	Estimate	95% CI	p-vaue
Treatment (group over self)	-1.020	[-4.177, 2.137]	0.515
Treatment (TAU over self)	-0.631	[-3.971, 2.71]	0.704
EssenCES ZP at T1	0.489	[0.288, 0.69]	0.000
Age (years)	-0.104	[-0.82, 0.613]	0.771
Gender (female over male)	-1.260	[-3.518, 0.999]	0.264
MAYSI II AI	-0.030	[-0.715, 0.655]	0.929
MAYSI II DA	0.315	[-0.232, 0.862]	0.250

Table 79: Results of the analysis for outcome EssenCES ZP with self-helped training as reference group. The results represent the pooled estimates with corresponding 95% confidence interval and p-value of the linear mixed effects model. The analysis was performed in the imputed PP analysis set (imputations in accordance with specified methods).

	Estimate	95% CI	p-vaue
Treatment (group over self)	1.259	[-1.831, 4.349]	0.413
Treatment (TAU over self)	0.043	[-3.116, 3.201]	0.978
EssenCES TH at T1	0.550	[0.317, 0.782]	0.000
Age (years)	0.124	[-0.543, 0.791]	0.709
Gender (female over male)	0.669	[-1.409, 2.748]	0.516
MAYSI II AI	0.551	[-0.069, 1.171]	0.080
MAYSI II DA	-0.249	[-0.749, 0.251]	0.319

Table 80: Results of the analysis for outcome EssenCES TH with self-helped training as reference group. The results represent the pooled estimates with corresponding 95% confidence interval and p-value of the linear mixed effects model. The analysis was performed in the imputed PP analysis set (imputations in accordance with specified methods).

	Estimate	95% CI	p-vaue
Treatment (group over self)	5.060	[-2.957, 13.077]	0.209
Treatment (TAU over self)	4.961	[-3.102, 13.025]	0.221
CD-RISC caretaker total at T1	0.230	[0.02, 0.44]	0.033
Age (years)	-0.342	[-1.715, 1.03]	0.617
Gender (female over male)	1.366	[-2.548, 5.28]	0.485
MAYSI II AI	-0.113	[-0.945, 0.719]	0.785
MAYSI II DA	0.494	[-0.207, 1.196]	0.162

Table 81: Results of the analysis for outcome CD-RISC total caretaker with self-helped training as reference group. The results represent the pooled estimates with corresponding 95% confidence interval and p-value of the linear mixed effects model. The analysis was performed in the imputed PP analysis set (imputations in accordance with specified methods).

	Estimate	95% CI	p-vaue
Treatment (group over self)	-2.486	[-10.129, 5.157]	0.514
Treatment (TAU over self)	-2.061	[-10.298, 6.177]	0.615
CIS total at T1	0.335	[0.003, 0.667]	0.048
Age (years)	1.111	[-0.629, 2.851]	0.204
Gender (female over male)	2.018	[-3.276, 7.313]	0.445
MAYSI II AI	-1.306	[-2.86, 0.247]	0.097
MAYSI II DA	1.031	[-0.258, 2.32]	0.114

Table 82: Results of the analysis for outcome CIS total with self-helped training as reference group. The results represent the pooled estimates with corresponding 95% confidence interval and p-value of the linear mixed effects model. The analysis was performed in the imputed PP analysis set (imputations in accordance with specified methods).

Final Report

	Estimate	95% CI	p-vaue
Treatment (group over self)	-0.050	[-2.7, 2.599]	0.970
Treatment (TAU over self)	0.448	[-2.336, 3.233]	0.747
ARI caretaker total at T1	0.455	[0.111, 0.799]	0.011
Age (years)	-0.260	[-1.018, 0.497]	0.492
Gender (female over male)	1.132	[-0.833, 3.098]	0.252
MAYSI II AI	-0.050	[-0.569, 0.468]	0.845
MAYSI II DA	0.061	[-0.372, 0.494]	0.777

Table 83: Results of the analysis for outcome ARI total caretaker with self-helped training as reference group. The results represent the pooled estimates with corresponding 95% confidence interval and p-value of the linear mixed effects model. The analysis was performed in the imputed PP analysis set (imputations in accordance with specified methods).

Final Report

6.2.3 Supplementary analysis: interactions with baseline particpant characteristics

	Estimate	95% CI	p-vaue
Treatment (group over TAU)	-1.329	[-11.311, 8.653]	0.791
Treatment (self over TAU)	-0.716	[-11.71, 10.277]	0.897
Previous institutionalization	-3.164	[-12.244, 5.916]	0.490
AFQ total at T1	0.275	[0.026, 0.524]	0.031
Age (years)	0.312	[-1.654, 2.279]	0.751
Gender (female over male)	1.306	[-4.941, 7.554]	0.677
Gender (diverse over male)	2.697	[-31.328, 36.721]	0.874
MAYSI II AI	-1.036	[-2.44, 0.368]	0.145
MAYSI II DA	-0.060	[-1.474, 1.354]	0.933
Treatment (group over TAU):Previous institutionalization	2.246	[-9.879, 14.372]	0.713
Treatment (self over TAU):Previous institutionalization	0.424	[-12.293, 13.141]	0.947

Table 84: Results of the supplementary analysis testing the interaction between treatment and previous stay in institution or care family with TAU as reference group. The results represent the pooled estimates with corresponding 95% confidence interval and p-value of the linear mixed effects model. The analysis was performed in the imputed PP analysis set (imputations in accordance with specified methods).

	Estimate	95% CI	p-vaue
Treatment (group over TAU)	-2.514	[-63.093, 58.066]	0.934
Treatment (self over TAU)	-8.540	[-79.375, 62.294]	0.811
Age (years)	0.215	[-2.351, 2.781]	0.868
AFQ total at T1	0.297	[0.086, 0.508]	0.006
Gender (female over male)	1.641	[-3.432, 6.715]	0.521
Gender (diverse over male)	1.430	[-32.379, 35.239]	0.933
MAYSI II AI	-1.058	[-2.283, 0.166]	0.089
MAYSI II DA	-0.036	[-1.284, 1.212]	0.955
Treatment (group over TAU):Age (years)	0.075	[-3.534, 3.685]	0.967
Treatment (self over TAU):Age (years)	0.450	[-3.824, 4.723]	0.834

Table 85: Results of the supplementary analysis testing the interaction between treatment and diagnosis of psychological disease with TAU as reference group. The results represent the pooled estimates with corresponding 95% confidence interval and p-value of the linear mixed effects model. The analysis was performed in the imputed PP analysis set (imputations in accordance with specified methods).

Final Report

Final Report

	Estimate	95% CI	p-vaue
Treatment (group over TAU)	-3.120	[-13.106, 6.865]	0.534
Treatment (self over TAU)	-1.699	[-11.387, 7.989]	0.727
Diagnosis of psychological disease	-0.240	[-10.148, 9.668]	0.962
AFQ total at T1	0.286	[0.027, 0.545]	0.031
Age (years)	0.429	[-1.619, 2.478]	0.676
Gender (female over male)	0.775	[-5.454, 7.005]	0.804
Gender (diverse over male)	1.049	[-33.701, 35.798]	0.952
MAYSI II AI	-1.057	[-2.512, 0.398]	0.151
MAYSI II DA	-0.030	[-1.514, 1.454]	0.968
Treatment (group over TAU):Diagnosis of psychological disease	5.877	[-7.757, 19.511]	0.392
Treatment (self over TAU):Diagnosis of psychological disease	1.682	[-12.3, 15.665]	0.810

Table 86: Results of the supplementary analysis testing the interaction between treatment and age with TAU as reference group. The results represent the pooled estimates with corresponding 95% confidence interval and p-value of the linear mixed effects model. The analysis was performed in the imputed PP analysis set (imputations in accordance with specified methods).

	Estimate	95% CI	p-vaue
Treatment (group over TAU)	-0.665	[-8.754, 7.425]	0.871
Treatment (self over TAU)	-0.425	[-8.736, 7.887]	0.919
Duration of institutionalization	1.431	[-8.225, 11.088]	0.768
AFQ total at T1	0.288	[0.036, 0.54]	0.026
Age (years)	0.264	[-1.722, 2.249]	0.791
Gender (female over male)	0.772	[-5.499, 7.043]	0.806
Gender (diverse over male)	1.514	[-33.013, 36.041]	0.930
MAYSI II AI	-0.983	[-2.376, 0.411]	0.163
MAYSI II DA	-0.067	[-1.473, 1.34]	0.925
Treatment (group over TAU):Duration of institutionalization	0.933	[-12.417, 14.283]	0.889
Treatment (self over TAU):Duration of institutionalization	-1.343	[-13.781, 11.096]	0.830

Table 87: Results of the supplementary analysis testing the interaction between treatment and duration of institutionalization with TAU as reference group. The results represent the pooled estimates with corresponding 95% confidence interval and p-value of the linear mixed effects model. The analysis was performed in the imputed PP analysis set (imputations in accordance with specified methods).

Final Report

7 Deviations from report and analysis plan (RAP)

[1] The supplementary objectives regarding caregiver characteristics were not performed since the available data did not allow it.

[2] The effect of the caregiver's previous experience with START NOW could not be assessed, as only caregivers in the group-based therapy had experience with START NOW.

[3] The secondary endpoints were reported by the respective caretakers on a participant-level. No specific caretaker analysis was performed, except for a description of the caretaker characteristics.

[4] Supplementary analyses evaluating the effects of caretaker characteristics could not be performed due to missingness or inconsistency of the necessary variables.

[5] It was not described how to define the variables included in the interaction tests, but in the relatively small dataset with many predefined confounders, dichotomising the variables provides us with an idea if this baseline participant characteristic in question affects the treatment effect.

References

Eekhout, I.; De Vet, H. C.; Twisk, J. W.; Brand, J. P.; de Boer, M. R. & Heymans, M. W. 2014: Missing data in a multi-item instrument were best handled by multiple imputation at the item score level. Journal of clinical epidemiology 67(3):335–342.

Eekhout, I.; de Vet, H. C.; de Boer, M. R.; Twisk, J. W. & Heymans, M. W. 2018: Passive imputation and parcel summaries are both valid to handle missing items in studies with many multi-item scales. Statistical methods in medical research 27(4):1128–1140.

R Core Team. 2023: R: A Language and Environment for Statistical Computing. R Foundation for Statistical Computing.

Rubin, D. B. 1987: Multiple Imputation for Nonresponse in Surveys. John Wiley & Sons.
