# Supplementary material for: Implementation and effectiveness of the web‑based skills training START NOW in adolescents in residential youth care: a cluster randomized controlled trial
Source: Child Adolesc Psychiatry Ment Health. 2026 Jun 29;20:131. doi: 10.1186/s13034-026-01127-z (PMC13591857; doi:10.1186/s13034-026-01127-z)
Supplement: Supplementary file 4 [file 13034_2026_1127_MOESM4_ESM.pdf]

Universität Basel
Departement
Klinische Forschung

Universitätsspital Basel

Universität Basel, Departement Klinische Forschung, c/o Universitätsspital Basel, Spitalstrasse 12, 4031 Basel

Prof. Dr. Dr. Christina Stadler
Klinik für Kinder und Jugendliche der
Universitären Psychiatrischen Kliniken
Schanzenstrasse 13
4056 Basel

Final Report

Basel, June 16, 2025

Methods & Statistics

Study Title	Implementierung einer e-Version des Skillstrainings START NOW zur Förderung der Emotionsregulation und Resilienz in Institutionen des Massnahmenvollzugs
Internal DKF ID	pk19Stadler
Document Status	Final Report

Universität Basel
Departement Klinische Forschung
c/o Universitätsspital Basel
Spitalstrasse 12
4031 Basel, Switzerland

Nikki Rommers, PhD
Statistician, Department of Clinical Research
Telefon +41 328 77 14
nikki.rommers@usb.ch
dkf.unibas.ch

Final Report

1 Introduction

This report contains supplementary analyses of the randomized controlled trial investigating the effectiveness of the group-based and e-Version of the skills training START NOW in institutionalized youth. The primary analysis, as specified in the published protocol and analysis plan did not show any differences between the conditions. However, it was noticed that the compliance to the program was very low in the group receiving the e-version of START NOW, with more than 50% of the participants in this group not even participating in one session. Because of the low compliance rate, this supplementary analysis investigates the effect of receiving the START NOW training (defined as completing at least 5 sessions), either as group-based training or online training on the outcomes of the study.

Additionally, we investigated if there is a potential dose-response relationship for the number of START NOW sessions and the outcomes.

1.1 Population and analysis set

This analysis uses all participants of the trial. Due to the very large proportion of missing data for most outcomes (see main report) and the corresponding low certainty of imputations, this analysis uses the available data to carefully look for signs of potential treatment effects.

For the main analysis, participants are divided in two groups: START NOW vs. no START NOW. The "START NOW" group included participants randomized in the group-based or online training group, who completed at least 5 sessions of the START NOW program. All patients randomized to TAU and participants in the other two groups who did not complete at least 5 sessions, were allocated to the "no START NOW" group. Based on these data, we allocated 64 participants to the START NOW group, and 101 to the no START NOW group.

For the second aim, the dose-response relationship, we only included participants allocated to the group-based and e-version of START NOW. In other words, all participants who had access to the START NOW program and had a number of sessions reported. We included 53 participants in the self-guided group and 54 in the group-based training group.

2 Methods

The fit mixed-effects linear regression models with a fixed effect for group or number of sessions, and a random effect for institution. Additionally, we adjusted each of the models for the baseline score of the respective questionnaire by adding this score as a fixed effect to the model. We did this because we know there is a strong correlation between the score at baseline and the scores at other time points. The low number of participants with available data did not allow adjustment for additional covariates. Model assumptions were inspected visually and accepted for all outcomes. Only in the analysis of the CD-RISC score at time point 2 and 4 with the 2 groups (START NOW vs. no START NOW), we had to remove the random effect due problems with the model fit. For each of the models we report the estimate with corresponding 95% profile confidence interval (CI) for the main effect of group or number of sessions. Additionally, we report the p-value. It must be noted that the p-values and CIs were not corrected for multiple testing. This means that results should be interpreted with caution and seen as exploratory and hypothesis generating rather than confirmatory.

Final Report

3 Results

In the tables below, we present the results of the supplementary analysis by objective and time point. In each of the tables, there is a column "number of observations" representing the number of observations valid to include in the analysis. This number is often much lower than the total population due to missing questionnaire data. The score is missing in case the total score could not be calculated due to one or more missing answers or questions answered with "do not wish to disclose". An overview of the proportion of missing data is available in the report of the main analysis.

3.1 START NOW vs. no START NOW

When comparing the two groups (START NOW vs. no START NOW) at the different time points, we see a trend of potentially higher PHQ4 scores in the START NOW group compared to the no START NOW group. This trend is not seen at T2 or T4. The CIS score by the caretaker might be lower in the START NOW group compared to the no START NOW group. This is a trend seen across all time points.

Variable	N. observations	Estimate	95% CI	p-value
AFQ total	60	1.468	[-3.077, 6.013]	0.529
CD-RISC total	37	-3.295	[-7.324, 0.734]	0.118
CD-RISC total caretaker	54	-1.337	[-6.249, 3.575]	0.597
WHO5 total	45	1.242	[-2.310, 4.793]	0.502
SWE total	45	0.076	[-5.781, 5.934]	0.980
PHQ4 total	41	2.070	[0.317, 3.823]	0.041
PHQ4 anxiety	43	0.164	[-0.800, 1.129]	0.740
PHQ4 depression	44	1.565	[0.675, 2.455]	0.003
ARI total	40	0.302	[-1.470, 2.074]	0.743
ARI total caretaker	59	-0.110	[-1.503, 1.283]	0.879
CIS total	31	1.774	[-3.895, 7.444]	0.545
CIS total caretaker	41	-1.789	[-5.268, 1.690]	0.320
EssenCES ZP	36	-0.491	[-2.918, 1.936]	0.694
EssenCES SE	37	1.163	[-1.144, 3.469]	0.335
EssenCES TH	33	1.178	[-1.129, 3.485]	0.325

Table 1: Overview table of the results for treatment (START NOW vs. no START NOW) for all outcomes at T3. The results represent estimates with corresponding 95% confidence interval and p-value of the linear mixed effects model, adjusted for the questionnair score at T1. The analysis was performed in the available data and we included the number of valid observations (N. observations) included in the analysis.

Variable	N. observations	Estimate	95% CI	p-value
AFQ total	56	-3.330	[-7.509, 0.849]	0.134
CD-RISC total	34	-1.580	[-6.302, 3.141]	0.500
CD-RISC total caretaker	88	3.558	[-0.502, 7.617]	0.093
WHO5 total	42	0.326	[-3.145, 3.797]	0.856
SWE total	39	0.574	[-2.628, 3.776]	0.731
PHQ4 total	40	0.047	[-1.702, 1.795]	0.959
PHQ4 anxiety	41	-0.611	[-1.663, 0.441]	0.269
PHQ4 depression	42	0.255	[-0.716, 1.226]	0.610
ARI total	36	-0.257	[-2.078, 1.564]	0.784
ARI total caretaker	92	-0.831	[-1.820, 0.159]	0.103
CIS total	33	-0.695	[-5.414, 4.024]	0.775
CIS total caretaker	55	-5.485	[-8.955, -2.015]	0.003
EssenCES ZP	33	-0.661	[-3.251, 1.929]	0.624
EssenCES SE	29	1.319	[-2.806, 5.444]	0.549
EssenCES TH	34	-0.122	[-3.136, 2.892]	0.937

Table 2: Overview table of the results for treatment (START NOW vs. no START NOW) for all outcomes at T2. The results represent estimates with corresponding 95% confidence interval and p-value of the linear mixed effects model, adjusted for the questionnair score at T1. The analysis was performed in the available data and we included the number of valid observations (N. observations) included in the analysis.

Final Report

Variable	N. observations	Estimate	95% CI	p-value
AFQ total	48	-4.073	[-11.037, 2.891]	0.261
CD-RISC total	36	0.444	[-5.435, 6.322]	0.879
CD-RISC total caretaker	40	4.290	[-1.497, 10.078]	0.171
WHO5 total	37	1.804	[-2.379, 5.987]	0.422
SWE total	37	-0.011	[-3.630, 3.608]	0.995
PHQ4 total	36	-1.312	[-2.788, 0.164]	0.101
PHQ4 anxiety	39	-0.836	[-1.634, -0.038]	0.055
PHQ4 depression	38	-0.323	[-1.146, 0.500]	0.447
ARI total	34	-0.213	[-1.956, 1.529]	0.815
ARI total caretaker	48	-0.763	[-1.821, 0.294]	0.176
CIS total	31	-1.874	[-11.107, 7.360]	0.697
CIS total caretaker	33	-3.904	[-9.498, 1.690]	0.182
EssenCES ZP	32	-0.110	[-2.752, 2.532]	0.936
EssenCES SE	29	3.392	[-0.692, 7.476]	0.120
EssenCES TH	29	1.622	[-1.728, 4.973]	0.358

Table 3: Overview table of the results for treatment (START NOW vs. no START NOW) for all outcomes at T4. The results represent estimates with corresponding 95% confidence interval and p-value of the linear mixed effects model, adjusted for the questionnair score at T1. The analysis was performed in the available data and we included the number of valid observations (N. observations) included in the analysis.

Final Report

3.2 Dose-response

We assumed a linear relation between the number of sessions and the outcomes. This was tested to be the best fitting model for AFQ total compared to a quadratic function. Across all time points, a higher number of sessions might be associated with a higher EssenCES score, particularly for the SE and TH subdomains. A higher number of sessions could be associated with a lower CIS score by the caretaker. However, this was not as clear across all time points and it must be noted that the number of available data points varied largely.

Variable	N. observations	Estimate	95% CI	p-value
AFQ total	37	0.480	[-0.273, 1.232]	0.223
CD-RISC total	23	0.091	[-0.982, 1.165]	0.870
CD-RISC total caretaker	36	0.227	[-0.261, 0.716]	0.369
WHO5 total	26	0.323	[-0.384, 1.029]	0.380
SWE total	24	-0.811	[-1.780, 0.157]	0.118
PHQ4 total	23	0.088	[-0.487, 0.662]	0.768
PHQ4 anxiety	23	0.105	[-0.221, 0.432]	0.534
PHQ4 depression	25	0.070	[-0.121, 0.261]	0.478
ARI total	21	-0.047	[-0.382, 0.289]	0.789
ARI total caretaker	40	0.105	[-0.051, 0.261]	0.207
CIS total	18	0.297	[-0.990, 1.584]	0.659
CIS total caretaker	28	-0.248	[-0.689, 0.193]	0.281
EssenCES ZP	18	-0.346	[-1.316, 0.623]	0.495
EssenCES SE	20	0.483	[-0.008, 0.973]	0.070
EssenCES TH	18	0.635	[0.091, 1.180]	0.045

Table 4: Overview table of the results for total number of sessions in association with for all outcomes at T3. The results represent estimates with corresponding 95% confidence interval and p-value of the linear mixed effects model, adjusted for the questionnair score at T1. The analysis was performed in the available data and we included the number of valid observations (N. observations) included in the analysis. The estimate represents the effect for one additional session and only includes participants in the START NOW groups who had the possibility to participate in sessions.

Final Report

Variable	N. observations	Estimate	95% CI	p-value
AFQ total	37	-0.226	[-0.858, 0.406]	0.489
CD-RISC total	23	0.453	[-0.366, 1.272]	0.292
CD-RISC total caretaker	65	0.675	[0.237, 1.113]	0.004
WHO5 total	27	0.224	[-0.306, 0.753]	0.418
SWE total	21	-0.204	[-0.733, 0.325]	0.461
PHQ4 total	23	0.123	[-0.102, 0.347]	0.297
PHQ4 anxiety	23	0.057	[-0.097, 0.212]	0.473
PHQ4 depression	25	0.088	[-0.063, 0.239]	0.264
ARI total	20	0.127	[-0.204, 0.458]	0.463
ARI total caretaker	68	-0.069	[-0.181, 0.043]	0.233
CIS total	22	0.197	[-0.515, 0.908]	0.594
CIS total caretaker	40	-0.626	[-1.065, -0.188]	0.011
EssenCES ZP	17	0.186	[-0.191, 0.564]	0.350
EssenCES SE	15	0.568	[0.119, 1.017]	0.031
EssenCES TH	18	0.470	[-0.103, 1.043]	0.129

Table 5: Overview table of the results for total number of sessions in association with for all outcomes at T2. The results represent estimates with corresponding 95% confidence interval and p-value of the linear mixed effects model, adjusted for the questionnair score at T1. The analysis was performed in the available data and we included the number of valid observations (N. observations) included in the analysis. The estimate represents the effect for one additional session and only includes participants in the START NOW groups who had the possibility to participate in sessions.

Variable	N. observations	Estimate	95% CI	p-value
AFQ total	26	-0.834	[-1.824, 0.156]	0.113
CD-RISC total	19	0.627	[-1.017, 2.271]	0.465
CD-RISC total caretaker	24	0.137	[-0.457, 0.730]	0.658
WHO5 total	19	0.863	[-0.537, 2.263]	0.246
SWE total	17	0.481	[-0.976, 1.938]	0.539
PHQ4 total	17	-0.171	[-0.361, 0.019]	0.117
PHQ4 anxiety	19	-0.173	[-0.338, -0.007]	0.061
PHQ4 depression	18	-0.024	[-0.172, 0.123]	0.754
ARI total	16	-0.115	[-0.577, 0.347]	0.634
ARI total caretaker	28	0.039	[-0.061, 0.139]	0.451
CIS total	15	-0.152	[-2.151, 1.847]	0.886
CIS total caretaker	18	-0.597	[-1.364, 0.170]	0.162
EssenCES ZP	14	0.168	[-0.848, 1.184]	0.752
EssenCES SE	13	0.553	[0.022, 1.084]	0.077
EssenCES TH	12	0.964	[0.321, 1.606]	0.016

Table 6: Overview table of the results for total number of sessions in association with for all outcomes at T4. The results represent estimates with corresponding 95% confidence interval and p-value of the linear mixed effects model, adjusted for the questionnair score at T1. The analysis was performed in the available data and we included the number of valid observations (N. observations) included in the analysis. The estimate represents the effect for one additional session and only includes participants in the START NOW groups who had the possibility to participate in sessions.
